# Recognition of HSPB8 as a potential therapeutic target for prostate cancer

**DOI:** 10.3389/fgene.2025.1680674

**Published:** 2025-10-21

**Authors:** Xun Fu, Yutao Wang, Hongjun Li

**Affiliations:** Department of Urology, Peking Union Medical Collage Hospital, Beijing, China

**Keywords:** HSPB8, prostate cancer, WGCNA, survival-related gene, AKT-mTOR signaling

## Abstract

Prostate cancer poses a serious burden on men’s quality of life. Identifying novel biomarkers for therapeutic development and prognostic prediction has long been a focal point in prostate cancer research. HSP family is a group of molecular chaperones that exhibit close relationship with many cancer types. In this study we screened out HSPB8 as a potential biomarker using WGCNA. Then we analyzed its expression patterns, investigated its biological functions, and assessed its prognostic values with a combination of bioinformatic analyses and experimental validation. Our data demonstrated that HSPB8 exhibited lower expression levels in prostate cancer tissues than in normal prostatic tissues. As a tumor suppressor gene, lack of HSPB8 was associated with unfavorable survival outcomes among patients with prostate cancer. In terms of biological function, HSPB8 were predominantly enriched in muscle-related biological processes, such as muscle contraction and muscle cell differentiation. On the molecular and cellular levels, HSPB8 silencing induced cellular proliferation and enhanced invasive and migratory capacities of prostate cancer cell lines. Its tumor-suppressive function was likely mediated through inactivation of PI3K−AKT signaling. Overall, this study offers a new understanding into the pathogenesis of prostate cancer, proposing that targeting HSPB8 might be a promising area in prostate cancer treatment.

## 1 Introduction

With 1,466,680 new diagnoses and 396,792 reported deaths in 2022, prostate cancer (PCa) has become the second most common cancer worldwide (Bray et al., 2024). Incidence exhibits significant geographic variation, with Northern Europe (82.8 per 100,000), Australia-New Zealand (82.8 per 100,000) and Caribbean (73.8 per 100,000) sharing the top three highest rates and several Asian regions (predominantly developing countries) reporting the lowest rates (Bray et al., 2024). In recent years, transitioning countries (e.g., China and some African countries) observe a rapid increase in new cases per year (Culp et al., 2020; Seraphin et al., 2021), indicating that prostate cancer has become a worldwide health problem. A consensus that the pathogenesis of prostate cancer is very complex has been established. Genetic and molecular alterations, chronic unresolved inflammation, persistent epithelial cell injury and many other pathophysiological processes have been all attributed to contributing factors (Kulac et al., 2024; Wilson and Zishiri, 2024). At present, immune properties of prostate cancer become a new focus for cancer research. Due to its low levels of tumor-infiltrating lymphocytes (TILs), reduced inflammatory signaling, low mutational burden and presence of immune checkpoint molecules, prostate cancer is immunologically categorized into “cold” tumor (Stultz and Fong, 2021). This suppressive tumor-immune microenvironment (TIME) leads to limited efficacy of present immunotherapy, such as immune checkpoint inhibitor (ICI), chimeric antigen receptor T-cell (CAR-T) and Sipuleucel-T (Anton et al., 2024). As a result, exploring new biomarkers for immunotherapy and developing novel immune agents have become a hot research topic of prostate cancer.

Heat shock protein (HSP) family is a group of highly-conserved molecular chaperones with cytoprotective properties (Gething and Sambrook, 1992). Under cellular stress conditions induced by various harmful stimulations, such as high temperature, infectious agents and hypoxia, most HSPs serve their cell-protective functions by either refolding or assisting in degrading misfolding proteins (Kriegenburg et al., 2012). Of note, not all HSPs are stress-inducible: some are constitutively expressed with relatively high expression levels in the absence of any stress so as to ensure protein’s correct folding (Gething and Sambrook, 1992; Bukau et al., 2006). These members are often named heat shock cognate. Scientists classified HSPs into six different subfamilies based on their molecular sizes: HSP110, HSP90, HSP70, HSP40, small HSPs, and chaperonin families—HSPD/E (HSP60/HSP10) and CCT (cytosolic chaperonin TCP1 ring complex, TRIC) (Rappa et al., 2012). HSP members in different subfamilies have different molecular structures. Such a large molecular family is involved in a myriad of biological processes apart from their chaperoning roles (i.e., protein folding and assembly), such as cell differentiation, signal transduction, immune regulation, programmed cell death and carcinogenesis (Rappa et al., 2012). A large amount of literature has associated HSPs, both experimentally and clinically, with initiation and progression of prostate cancer (Ratajczak et al., 2022; Fu et al., 2022; Saini and Sharma, 2018) and suggested that effective interventions targeting HSP members with either molecular inhibitors or genetic methods are expected to become a novel therapeutic regimen (Fu et al., 2022; Saini and Sharma, 2018). More recently, the roles of HSPs as immunomodulators have been revealed—they are implicated in antigen processing and presentation, activation of antigen-presenting cells (e.g., macrophages and dendritic cells), induction of immune cell proliferation and regulation of immune checkpoints (Zininga et al., 2018; Hagymasi et al., 2022). These immune functions of HSPs are associated with initiation and progression of many cancer types (Albakova and Mangasarova, 2021). However, little data concern prostate cancer in this regard.

In this article, we screened out a predictive biomarker HSPB8 from the HSP family with Weighted correlation network analysis (WGCNA). Using multiple bioinformatic and experimental methods, we studied HSPB8’s biological functions, explored the relationship between HSPB8 and patients’ survival, and investigated HSPB8’s impacts on TIME of prostate cancer.

## 2 Methods and materials

### 2.1 Data acquisition and processing

Gene expression matrix and clinical information of prostate cancer were downloaded from The Cancer Genome Atlas (TCGA) (http://cancergenome.nih.gov) (Accessed date: 14 May 2025). There were a total of 59,424 genes and 554 samples in the expression matrix with gene expression data normalized by log_2_ (exp+1). Downloaded clinical information matrix included phenotype and survival data. Of all clinical phenotypes, age, TNM stage, Gleason Score and prostate specific antigen (PSA) were the primary focus of this research.

### 2.2 Weighted gene co-expression network analysis

Using the R package “WGNCA”, we established a co-expression network by calculating weighted adjacency between every two genes. Hierarchical clustering is often the logical next step. Genes with similar expression patterns were categorized into the same gene module. In this study, HSP expression matrix that included 29 different HSP members was used as sample trait data since our goal is to find a module where genes have the highest similarity with HSP family in terms of expression patterns. Of all included HSP members, gene with the highest correlation coefficient shown in the heatmap was considered to be the gene of interest. Meanwhile, the correlation of this gene with which module is most significant could also be determined. The rationale and specific steps for WGCNA analysis have been summarized by Peter and Steve (Langfelder and Horvath, 2008).

### 2.3 Functional enrichment

For functional enrichment analysis, genes in the selected module with gene significance (with the gene of interest) > 0.6 were considered to be our targets. Gene Ontology (GO) (http://geneontology.org) (Accessed date: 17 May 2025) and Kyoto Encyclopedia of Genes and Genomes (KEGG, https://www.genome.jp/kegg) (Accessed date: 19 May 2025) were practical tools for investigating potential biological functions of those selected genes (Ashburner et al., 2000; Kanehisa and Goto, 2000). In GO enrichment analysis, three functional types, i.e., biological process (BP), molecular function (MF) and cellular component (CC), were all analyzed in the study. Result visualization was implemented using the R package “ggplot2”.

### 2.4 Single-cell analysis for gene distribution

Single-cell datasets of prostate cancer were accessible on the website Tumor Immune Single-cell Hub 2 (TISCH2) (http://tisch.compbio.cn/home) (Accessed date: 2 June 2025), an online scRNA-seq database focusing on tumor microenvironment (TME). In this study, we included 4 different prostate cancer databases (GSE_137829, GSE_141445, GSE_172301 and GSE_176031) and 13 different cell types including prostatic cells and immune cells to investigate expression patterns of the gene of interest within the prostate gland.

### 2.5 Univariate cox regression and survival analysis

After merging survival-related data (i.e., survival status (fustat) and survival time (futime)) into the gene expression matrix of selected module, we performed univariate cox regression using the R package “survival”. Hazard ratio (HR) of each gene and corresponding p value and KM value were calculated during the analysis process. Top ten genes in the selected module ranked by gene significance with both p value and KM less than preset pFilter (=0.05 in this study) were key genes for survival analysis. The R package “survminer” was used for result visualization.

### 2.6 Immune microenvironment analysis

We first employed the ESTIMATE algorithm to calculate stromal and immune scores of prostatic samples in the downloaded matrix. After merging survival data into the scoring file, we divided included specimens into high-score group and low-score group based on their score median. Then we implemented survival analysis using the R packages “survival” and “survminer” and compared expression levels of the gene of interest between the two groups.

### 2.7 Clinical correlation analysis

First, we extracted expression profile of the gene of interest and clinical data of all samples from the gene expression matrix and clinical information file, respectively. The clinical phenotypes that we focused on in this study included age, TNM stage, Gleason Score and PSA. Then, we divided the samples into different groups based on the clinical significance of each parameter (e.g., age into ≦60 years and >60 years; PSA into <4 ng/mL, 4–10 ng/mL and >10 ng/mL) and investigated whether or not there were statistically significant differences in terms of gene expression between samples from different groups.

### 2.8 Least absolute shrinkage and selection operator regression and prognostic model establishment

Least absolute shrinkage and selection operator (LASSO) regression is a very powerful tool in the medical landscape of outcome prediction. By selectively highlighting certain important parameters (or predictors) and removing non-critical ones, LASSO could construct a more refined model in the multivariate settings compared with other regression methods. In this study, genes in the selected module with gene significance (with the gene of interest) > 0.9 were considered to be the predictors and used to establish prognostic model with LASSO regression. For model preliminary validation, we performed Kaplan-Meier survival analysis, where p-value and hazard ratio (HR) with 95% confidence interval (CI) were calculated using log-rank test and univariate cox proportional hazards regression.

### 2.9 Cell culture

Human prostate cancer cell lines DU145, 22Rv1 were purchased from the National Collection of Authenticated Cell Cultures (Shanghai, China). Two cell lines were cultured in RPMI 1640 medium with 10% fetal bovine serum (FBS) at 37 °C in 5% CO_2_ atmosphere.

### 2.10 Cell transfection

Knocking down the gene of interest was achieved using pre-designed small interfering RNA (siRNA), with negative control siRNA serving as a control. DU145 and 22Rv1 cell lines were seeded in 6 well plates and cultured for at least 24 h before transfection. The transfection reaction mix was prepared with Opti-MEM reduced serum medium and Lipofectamine^®^2000 (Invitrogen, United States) following the manufacturer’s instructions. Western blot and quantitative PCR (qPCR) were performed to assess the efficiency and duration of gene knockdown.

### 2.11 Cell counting Kit-8 (CCK8) assay

Cellular proliferation was assessed using CCK8 assay. DU145 and 22Rv1 cells were seeded in a 96-well plate and cultured for different time intervals upon siRNA transfection. CCK-8 solution (Sangon Biotech, Shanghai, China) was added to each well, and the plate was then incubated in the dark for 1 h. The absorbance reading at 450 nm was measured using a microplate reader (Thermo Labsystems, Vantaa, Finland).

### 2.12 EDU staining

EdU staining was applied for cell proliferation assessment. DU145 and 22Rv1 cell lines were seeded into 24-well plates after siRNA transfection. Cell staining was performed using BeyoClick™ EdU-488 Cell Proliferation Kit (Beyotime Biotechnology, China), cell images were captured using a fluorescent microscope, and cell numbers were counted using ImageJ software (National Institutes of Health, Bethesda, Maryland).

### 2.13 Transwell assay

DU145 and 22Rv1 cell lines were seeded in 24-well plates with transwell chambers (8 μm pore size) (Corning Costar, Corning, NY, United States). Chambers with or without Matrigel (BD, San Diego, CA, United States) were employed to assess cell invasion and migration, respectively. After incubated for 48 h, cells in the plate were stained with 1.0% crystal violet. Cell images were captured using an inverted microscope, and cell counting was implemented using ImageJ.

### 2.14 Colony formation assay

Cells were suspended and plated into a six-well plate. Following a 2-week incubation, colonies were fixed with paraformaldehyde and stained with crystal violet solution, and then photographed and counted.

### 2.15 Total RNA extraction, reverse transcription and quantitative real time PCR (qRT-PCR)

Total RNA extraction was implemented using RaPure Total RNA Micro Kit (Magen, China) and Trizol reagent (Invitrog, Carlsbad, CA, United States). According to manufacturers’ instructions a total of 1 μg extracted RNA was reverse-transcribed into cDNA with ABScript II RT Master Mix (ABclonal, Wuhan, China). Gene amplification was performed by qRT-PCR on Bio-Rad CFX96 system (Hercules, CA, United States) and primers used in experiments were listed here: HSPB8: Forward: 5′-ACCAAAGATGGATACG TGGAGG-3′, Reverse: 5′-TGG​GGA​AAG​TGA​GGC​AAA​TAC​T-3'; β-actin: Forward: 5′-TCC​CTG​GAG​AAG​AGC​TAC​GA-3′, Reverse: 5′-TGA​AGG​TAG​TTT​CGT​GGA​TG C-3'. β-actin was used as control. Relative mRNA expression of a gene was calculated using 2^−ΔΔCT^ method.

### 2.16 Western blot analysis

Total protein was extracted using Radioimmunoassay Buffer (Shanghai Beyotime Biotechnology Co., Ltd., Shanghai, China). Following concentration and purity assessment of extracted protein, a total of 20 μg samples were electrophoretically separated on 10% sodium dodecylsulfate-polyacrylamide (SDS-PAGE) gels (Wuhan Boster Biological Technology Ltd., Wuhan, China) at 80 V. The protein samples were then transferred onto polyvinylidene fluoride (PVDF) membranes (Millipore, Billerica, MA, United States) at a constant current of 274mA, followed by incubation of PVDF membranes with transferred proteins in 5% skimmed milk for 2 h. Washed in PBS for three times, the PVDF membrane was incubated in primary antibody (Table 1) overnight at 4 °C and then in secondary antibody (Table 2) for 2 h at room temperature. Washed again, the signal on bands was detected using enhanced chemiluminescence kit (Thermo Scientific Fisher, Waltham, MA, United States) on Tanon-5200 ECL imager (Tanon, Shanghai, China). Densitometric quantification of protein on each band was performed using ImageJ software.

**TABLE 1:** Primary antibodies for Western blot.

Antigens	Species & type	Dilution	Supplier
HSPB8	Rabbit, polyclonal	1:1,000 (WB)	Abclonal (A2514)
AKT1	Rabbit, monoclonal	1:1,000(WB)	Abclonal (A17909)
pAKT	Rabbit, monoclonal	1:1,000(WB)	Abclonal (AP0637)
mTOR	Rabbit, polyclonal	1:1,000(WB)	Abclonal (A2445)
pmTOR	Rabbit, monoclonal	1:1,000(WB)	Abclonal (AP0115)
β-actin	Rabbit, polyclonal	1:1,000 (WB)	Abclonal (AC006)

**TABLE 2:** Secondary antibodies for Western blot.

Antigens	Species	Dilution	Supplier
Anti-Rabbit-IgG (H + L)-HRP	Goat	1:10,000 (WB)	Sungene Biotech, China, Cat. #LK2001

### 2.17 Statistical analysis

All bioinformatic analyses were implemented on R software (v 4.4.3). R packages used for analysis were accessible online and could be freely downloaded. All experiments were performed at least three times. Data were presented as mean ± standard deviation (SD). Apart from R packages, GraphPad Prism v 5.01 and SPSS v 25.0 were also the statistical tools applied in the study. p < 0.05 was considered to be statistically significant.

## 3 Results

### 3.1 Gene screening through WGCNA

**FIGURE 1:**
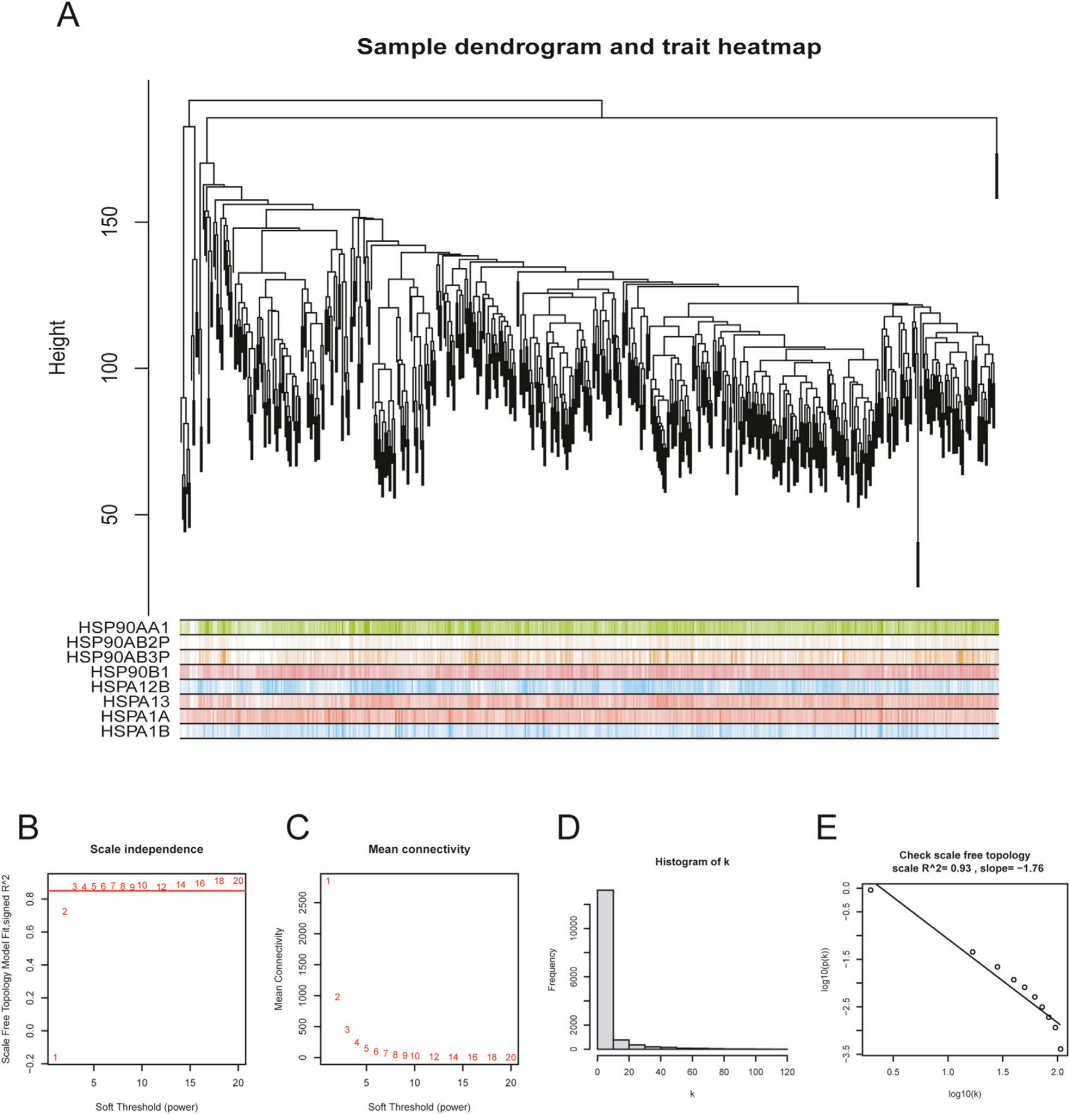
Co-expression network establishment. **(A)** sample dendrogram for preliminary hierarchical clustering; **(B–E)** soft-thresholding power determination and corresponding scale-free topology *R* ^2^ calculation (β = 3, *R* ^2^ = 0.93 in this study).

**FIGURE 2:**
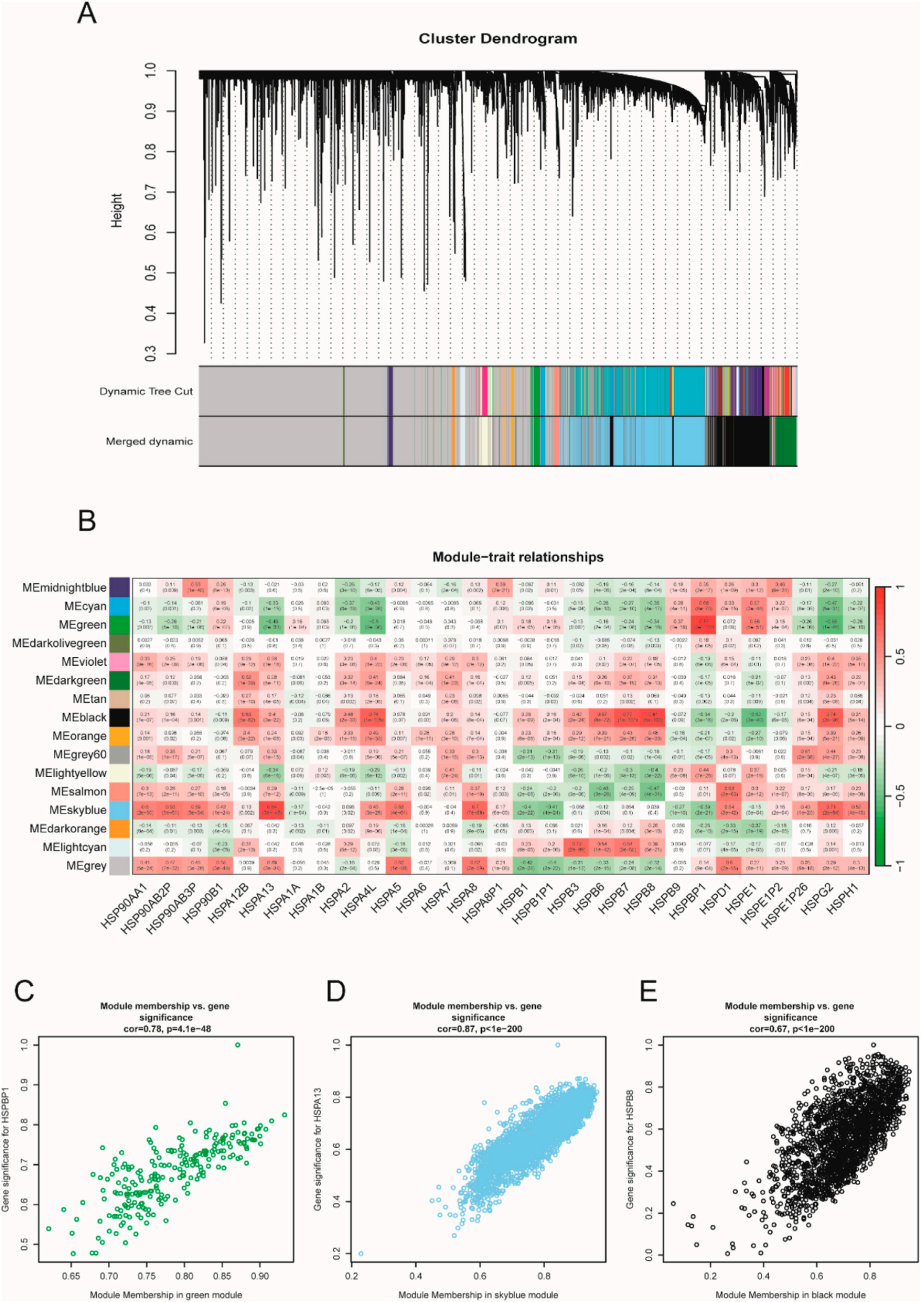
Sample clustering and gene module selection. **(A)** cluster dendrogram; **(B)** module-trait relationship heatmap; **(C–E)** correlation analysis between module membership and gene significance.

Before performing WGCNA analysis, we firstly screened out genes with the top 25% variant across all samples to reduce the computational workload in the following steps. (14,856 genes remaining). After removing 4 outlier samples, we constructed a co-expression network using the remaining 550 samples and 14,856 genes, with the soft-thresholding power beta = 3 and its corresponding scale-free topology *R* ^2^ = 0.93 (Figures 1A–E). Hierarchical clustering is usually the next step. Genes with similar expression patterns were grouped into the same gene module (Figure 2A). Given that this study aimed to identify a module where genes have the highest expression pattern similarity with the HSP family, HSP expression matrix (29 genes in total) was included and used as sample trait data. The module-trait heatmap showed that there were three HSP genes—HSPBP1, HSPA13 and HSPB8 — showing relatively high correlation coefficient (>0.8) with ME green (0.87), ME skyblue (0.84) and ME black (0.81), respectively (Figure 2B). Module membership in ME green, ME skyblue and ME black significantly correlated with gene significance for HSPBP1, HSPA13 and HSPB8, respectively (Figures 2C–E). However, pan-cancer data from GEPIA showed significant differences in terms of HSPB8 expression between normal prostatic tissues and prostate cancer samples while this was not the case for HSPBP1 and HSPA13 (Supplementary Figures S1A–C). As a result, HSPB8 was considered to be the gene of interest.

### 3.2 HSPB8 expression patterns within the prostate gland

First, we employed the downloaded gene matrix to compare the expression of HSPB8, HSPBP1 and HSPA13 levels between normal and prostate cancer samples for validation purposes. HSPBP1 expression was significantly higher in prostate cancer groups than in normal controls (Supplementary Figure S1D), while HSPA13 and HSPB8 showed lower expression levels among prostate cancer tissues (Supplementary Figures S1E,F).

**FIGURE 3:**
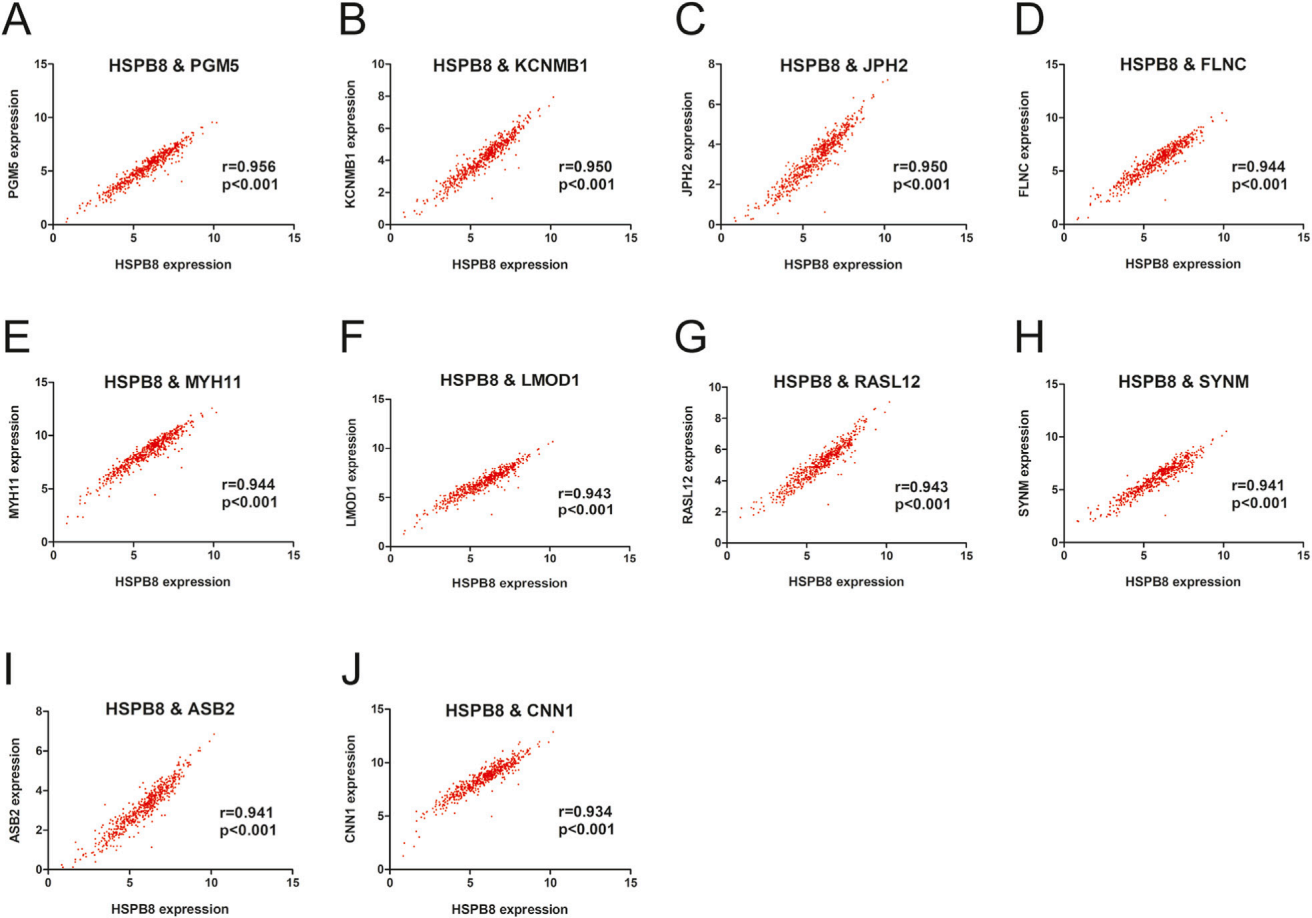
Gene expression correlation within the prostate gland. **(A–J)** Pearson correlation between HSPB8 and top ten genes in terms of gene expression levels.

There were a total of 31 genes (excluding HSPB8) with GS. HSPB8 > 0.9. We focused on the top ten genes ranked by GS. HSPB8 in this section and investigated whether their expression correlated with HSPB8. The focused genes included PGM5, KCNMB1, JPH2, FLNC, MYH11, LMOD1, RASL12, SYNM, ASB2 and CNN1. Our data demonstrated that these genes showed significant positive correlation with HSPB8 in terms of expression patterns, with r ranging from 0.934 to 0.956 and p < 0.001 (Figures 3A–J).

### 3.3 Functional enrichment analysis for HSPB8 and HSPB8-related genes

**FIGURE 4:**
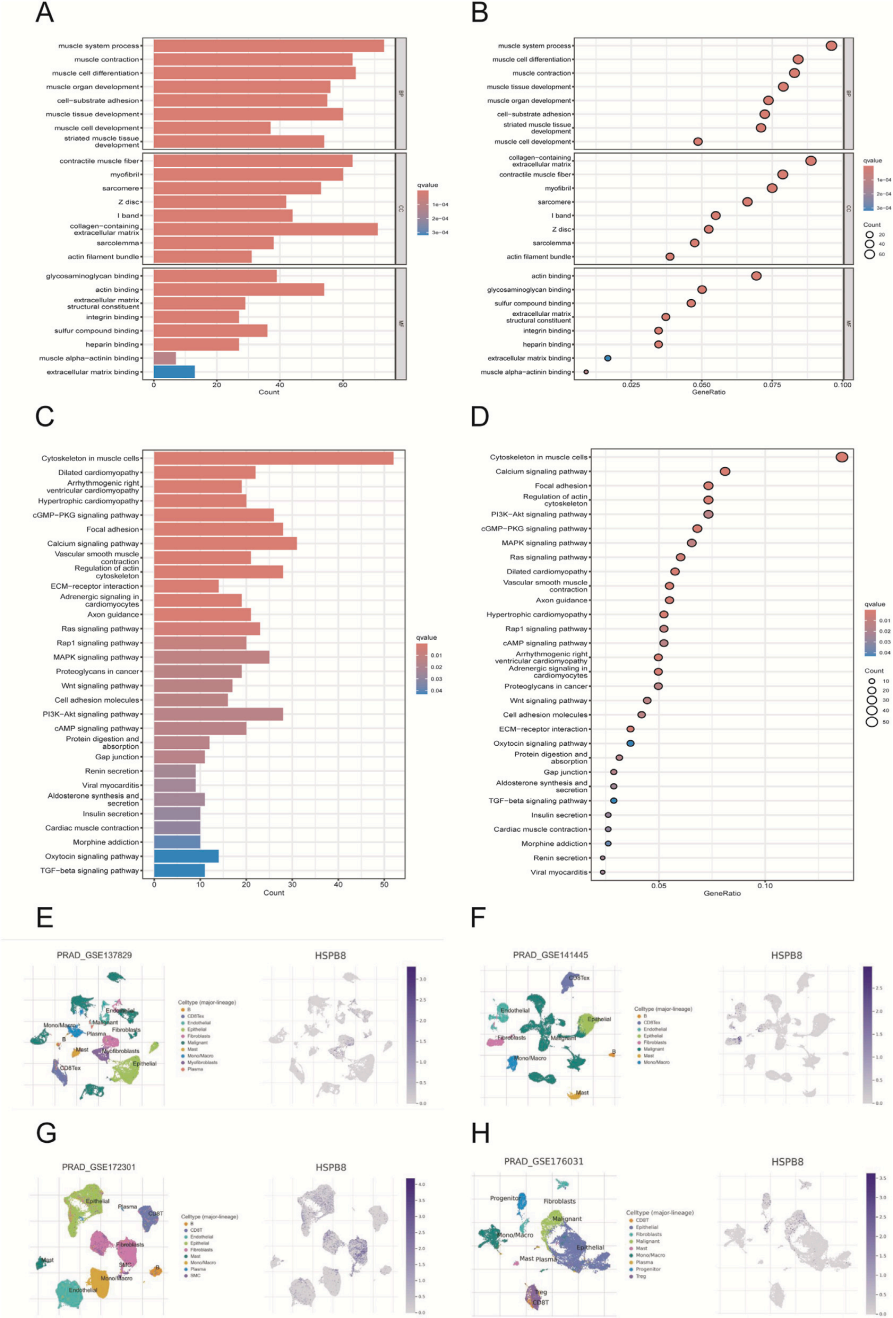
Functional enrichment and single-cell analysis. **(A,B)** GO enrichment analysis; **(C,D)** KEGG enrichment analysis; **(E–H)** single-cell analysis for gene distribution.

HSPB8-related genes was defined as genes in ME black with GS. HSPB8 > 0.6. Using the R package ClusterProfilter, we performed GO and KEGG enrichment analyses on HSPB8 and HSPB8-related genes (911 genes in total). Our results demonstrated that those selected genes were mainly enriched in muscle system process, collagen−containing extracellular matrix, muscle cell differentiation and muscle contraction (Figures 4A,B). And in KEGG analysis, cytoskeleton in muscle cells, calcium signaling pathway, and PI3K−AKT signaling pathway were the top three enriched KEGG pathways (Figures 4C,D). Further, we used online tool TISCH2 (http://tisch.compbio.cn/home) (Accessed date: 2 June 2025) and attached single-cell datasets of prostate cancer (GSE_137829, GSE_141445, GSE_172301 and GSE_176031) to detect HSPB8 distribution within the prostate gland. We found that HSPB8 was primarily enriched in fibroblasts (Figures 4E–H). Epithelial cells and endothelial cells also exhibited relatively high HSPB8 expression (Figures 4E–G).

### 3.4 Survival analysis for HSPB8

**FIGURE 5:**
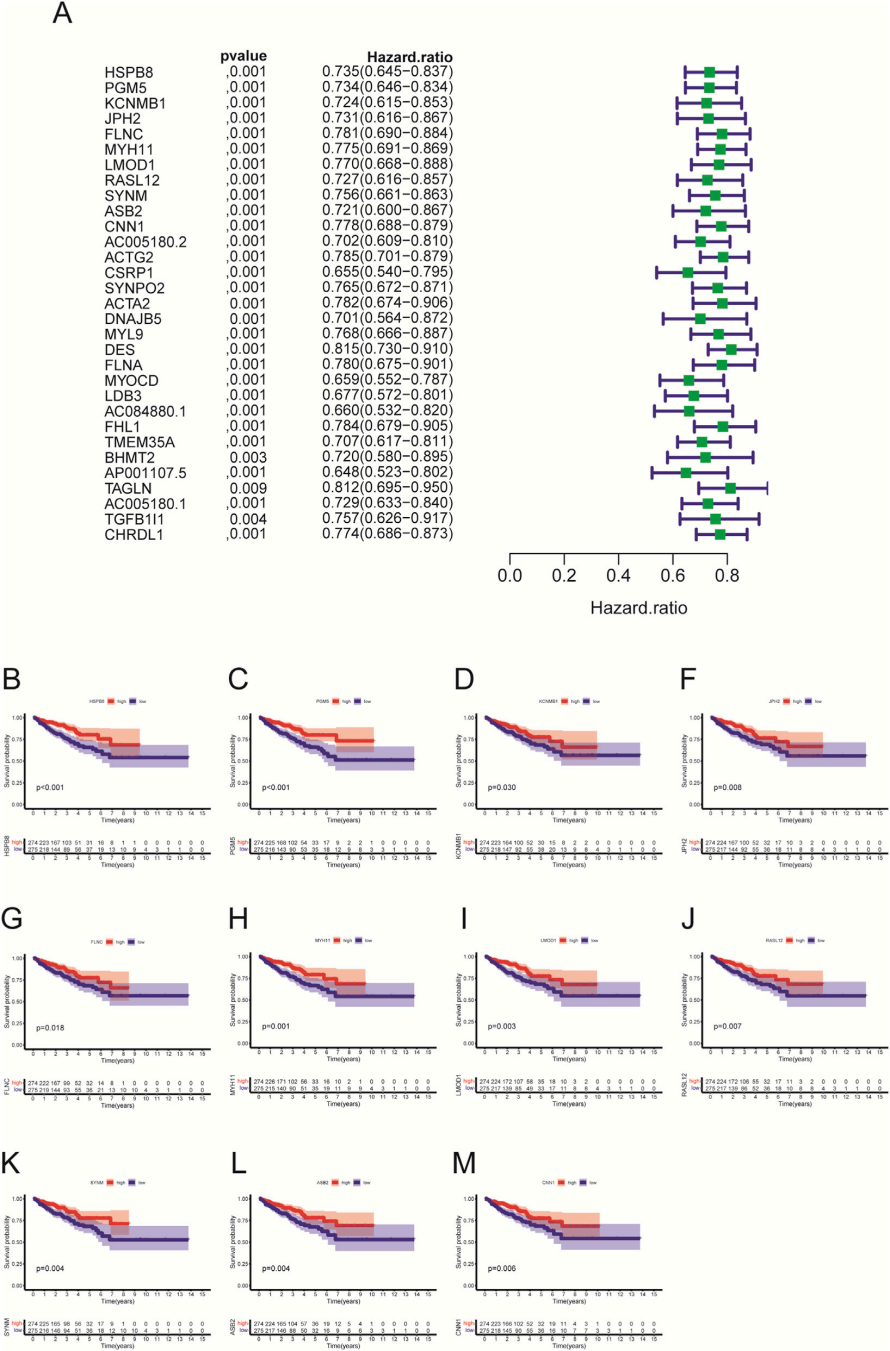
Univariate cox regression and survival analysis. **(A)** univariate cox regression; **(B–M)** survival analysis.

Using the R package “survival”, we identified whether HSPB8 was survival-related genes. First, we performed cox regression analysis and found that hazard ratio (HR) for HSPB8 was less than 1 (0.735, 95% CI: 0.645–0.837) (Figure 5A). Survival analysis was then implemented and survival curves demonstrated that low survival probability was observed among patients with low HSPB8 levels (p < 0.001) (Figure 5B). This was also the case for the top ten genes ranked by GS. HSPB8, i.e., PGM5, KCNMB1, JPH2, FLNC, MYH11, LMOD1, RASL12, SYNM, ASB2 and CNN1 (excluding HSPB8 itself). They all had HR < 1 and their low expression levels were observed among patient groups with low survival probability (p < 0.05) (Figures 5A,C–M).

### 3.5 Correlation of HSPB8 with different clinical phenotypes

**FIGURE 6:**
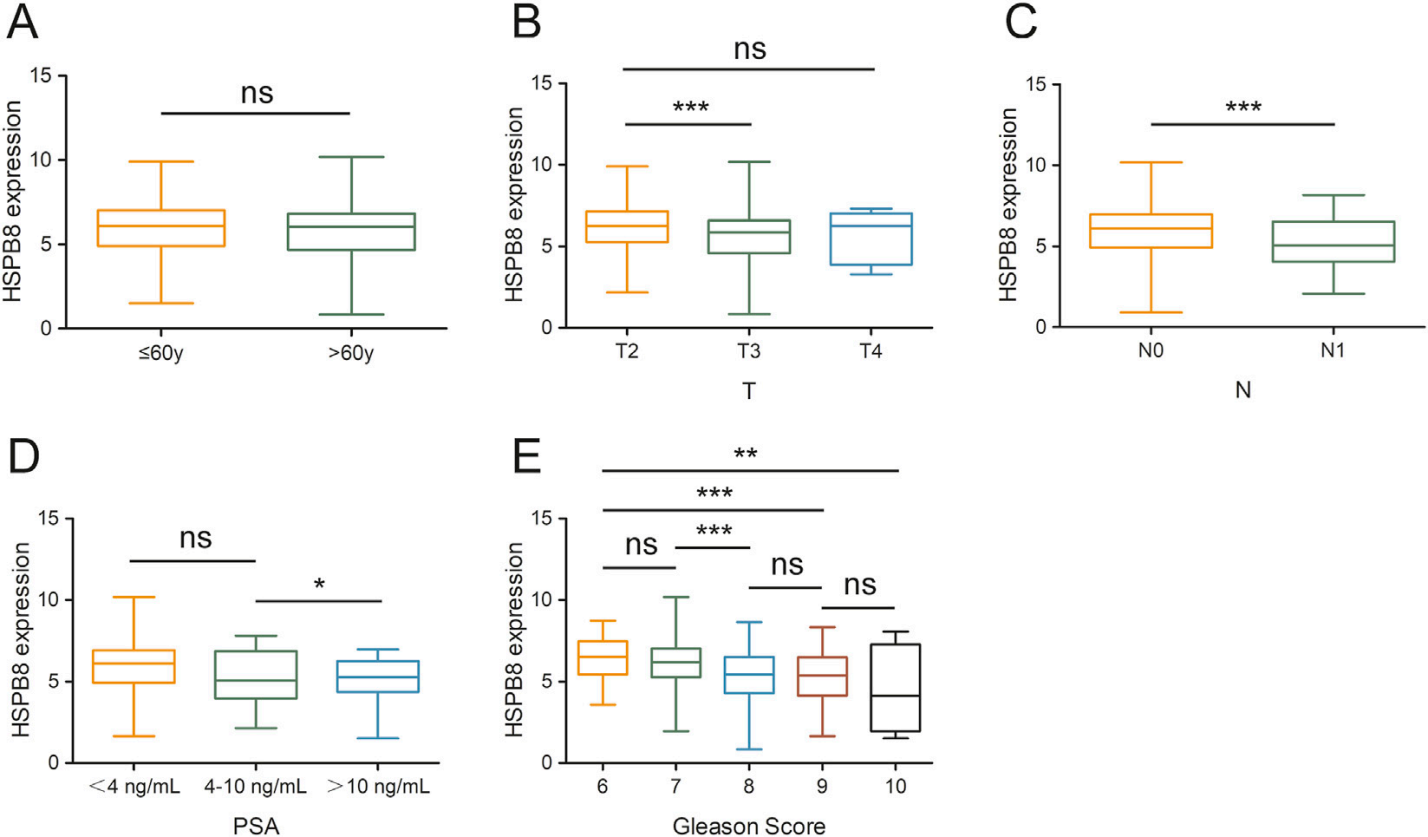
Gene expression differences across different patient groups. **(A)** age stratification: ≦ 60years and >60years; **(B)** tumor T stage stratification: T2, T3 and T4; **(C)** tumor N stage stratification: N0 and N1; **(D)** PSA level stratification: <4 ng/mL, 4–10 ng/mL and >10 ng/mL; **(E)** Gleason score stratification: 6, 7, 8, 9, 10. ns: p > 0.05, *: p < 0.05, **: p < 0.01, ***: p < 0.001.

In this section, we compared expression differences of HSPB8 among different patient groups. Clinical phenotypes we studied here included age, tumor T and N stage, Gleason score and PSA. Our data showed that there were no significant differences in HSPB8 expression between patients aged ≦ 60 years and >60 years (p = 0.201) (Figure 6A). HSPB8 expression was significantly higher in T2 groups than in T3 group (p < 0.001), but no significant differences were observed among groups T2 vs. T4 and T3 vs. T4 (Figure 6B). For tumor N stage, patients in N1 stage exhibited lower HSPB8 expression levels compared to those in N0 stage (p < 0.001) (Figure 6C). To investigate the correlation of HSBP8 with PSA, patients were divided into three groups based on their PSA levels: <4 ng/mL, 4–10 ng/mL and >10 ng/mL. HSPB8 expression was lower in patient groups with PSA ranging from 4 to 10 ng/mL than in those with PSA <4 ng/mL, but not significantly (p = 0.132), while patients with PSA >10 ng/mL had significantly lower HSPB8 expression compared to those with PSA <4 ng/mL (p < 0.05) (Figure 6D). For patients’ Gleason score, patient groups with Gleason score = 6 exhibited significantly higher HSPB8 levels compared to those with Gleason score = 9 and = 10 (p < 0.001 and p < 0.01, respectively), while no significant differences were observed among groups Gleason score 6 vs. 7, 8 vs. 9 and 9 vs. 10 (Figure 6E).

### 3.6 Immune microenvironment analysis

**FIGURE 7:**
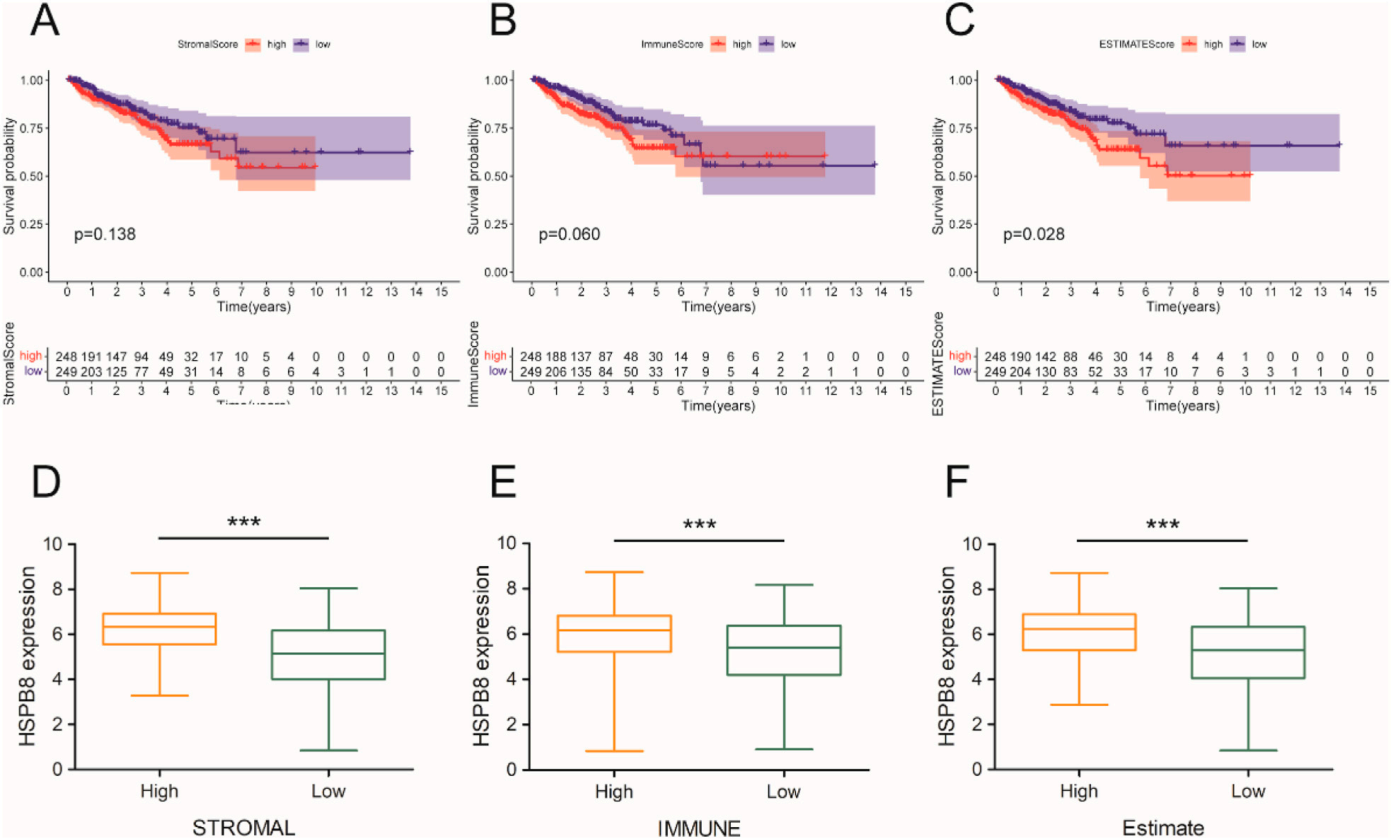
Immune microenvironment analysis. **(A–C)** survival analysis; **(D–F)** gene expression differences between the two groups. ***: p < 0.001.

Using the ESTIMATE algorism, we got the stromal score, immune score and ESTIMATE score of prostate cancer samples (normal samples had been excluded). Based on the score median, included specimens were divided into two groups: high-score group and low-score group. Then, we compared survival outcomes and HSPB8 expression levels between the two groups. The survival curve illustrated that patients with high stromal score, immune score and ESTIMATE score tended to have poorer survival outcomes (p = 0.138, p = 0.060 and p = 0.028, respectively) (Figures 7A–C). Across all three score metrics, HSPB8 was significantly higher in high-score group than in low-score group (p < 0.001) (Figures 7D–F).

### 3.7 Prognostic model construction

**FIGURE 8:**
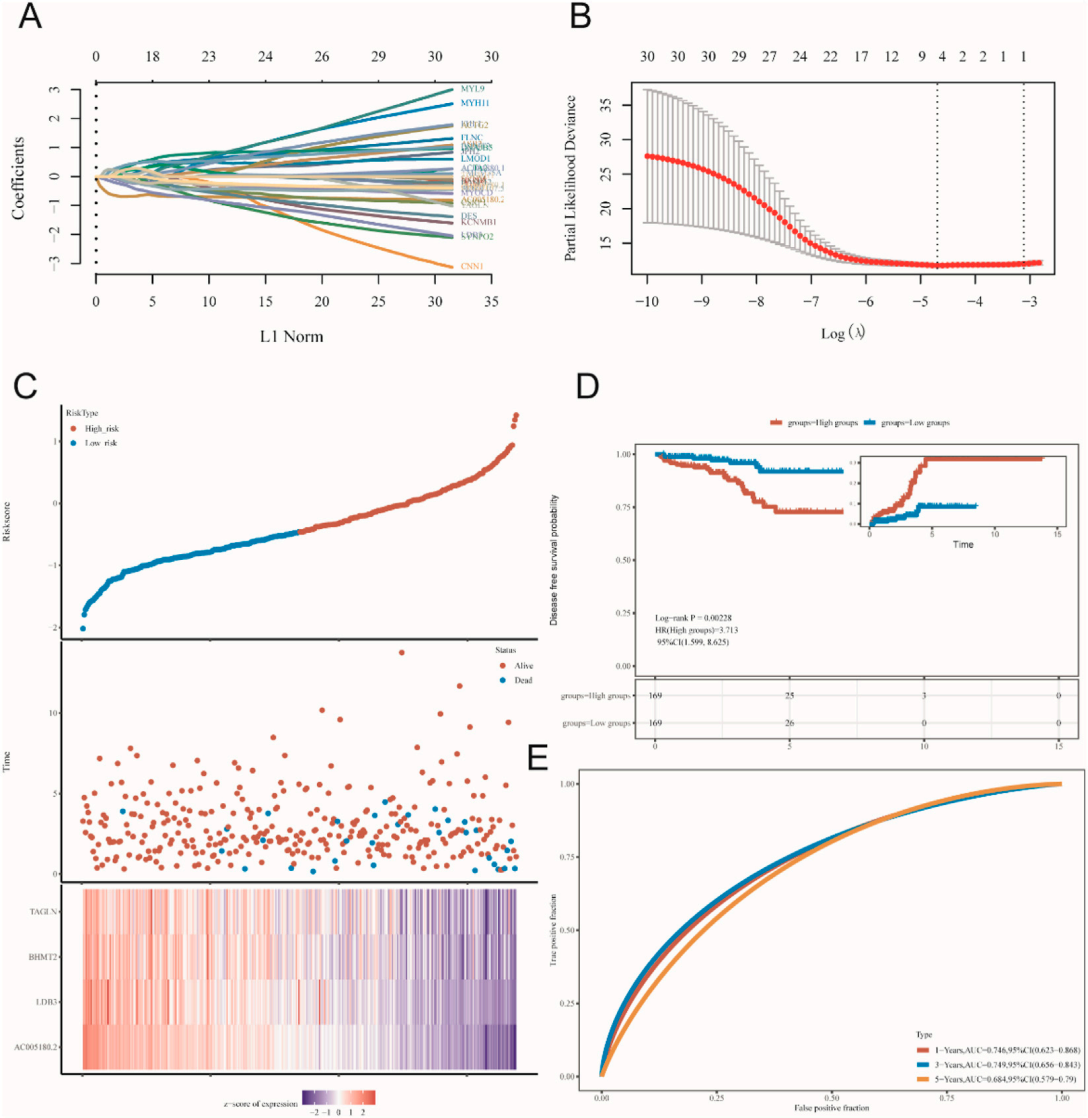
Prognostic model construction. **(A,B)** lambda. min determination in LASSO regression. **(C–E)** preliminary validation of established model.

Genes with GS. HSPB8 > 0.9 were employed to establish prognostic model (31 genes in total). A total of 498 prostate cancer samples were applied for model construction. The risk score could be calculated using the following equation: Risk score = (−0.5882)*AC005180.2 + (−0.1387)*LDB3 + (0.1349)*BHMT2 + (0.2432)*TAGLN, where lambda. min = 0.0092 (Figures 8A,B). Based on the median value of risk scores, prostate cancer samples were divided into two groups: high-risk group and low-risk group (Figure 8C). Our results showed that HR for the high-risk group was 3.713 (95% CI: 1.599–8.625) and they had poorer survival prospect compared to the low-risk group (p = 0.002) (Figure 8D). The areas under the receiver operating characteristic curve (AUC) for the model at three different time point: 1 year, 3 years and 5 years were 0.746 (95% CI: 0.623–0.868), 0.749 (95% CI: 0.656–0.843) and 0.684 (95% CI: 0.579–0.790), respectively.

### 3.8 Expression and function validation of HSPB8 in prostate cell lines

**FIGURE 9:**
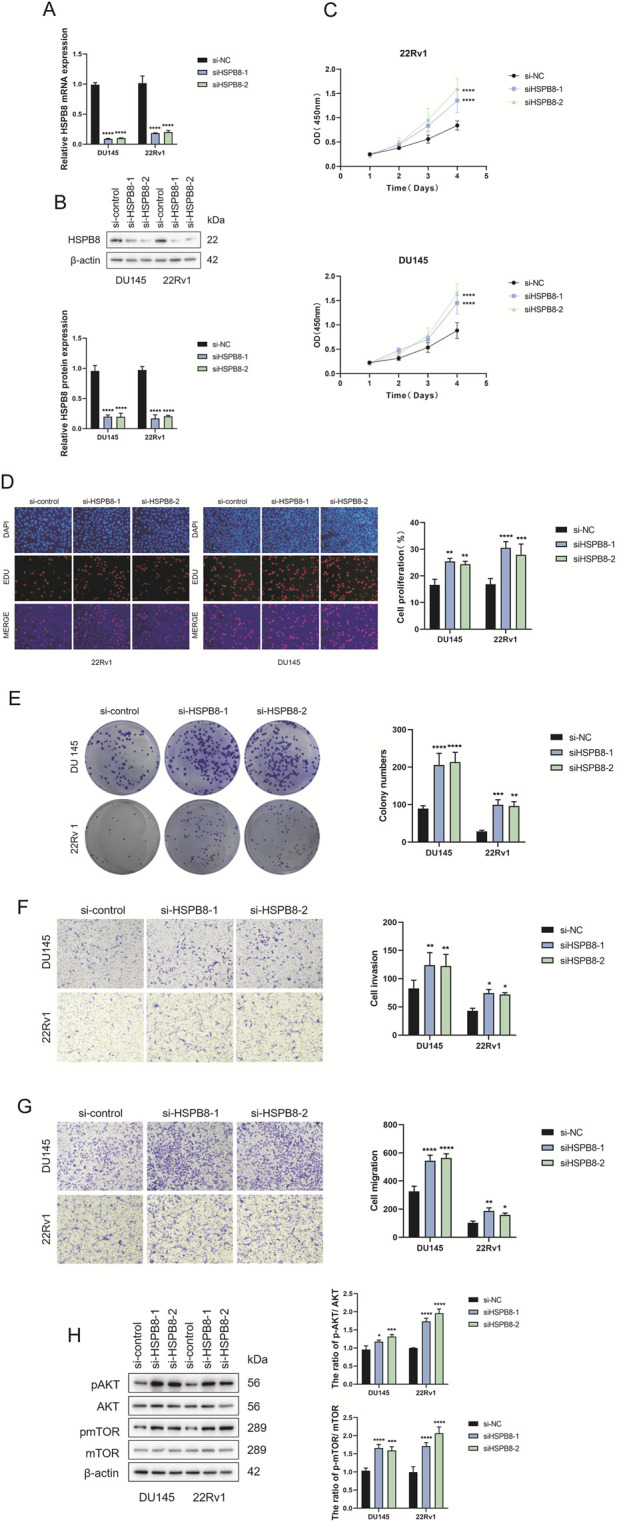
Expression and functional verification of HSPB8 using siRNA **(A,B)** Transfection efficacy validation at both transcriptional and translational levels. **(C)** Proliferative rates (OD value (450 mm)) of DU145 and 22Rv1 cell lines following HSPB8 knockdown at different time points. **(D)** Edu staining of HSPB8-silencing prostate cell lines. **(G)** Colony formation of two prostatic cell lines following HSPB8 silencing. **(E,F)** Invasion and migration ability of HSPB8-silencing cell lines. **(H)** Immunoblot assay and relative densitometric quantification of AKT, pAKT, mTOR and pmTOR expression in two lines after HSPB8 knockdown. β-actin is used as loading control. ^*^: p < 0.05; ^**^: p < 0.01; ^***^: p < 0.001; ^****^: p < 0.0001.

For gene expression verification in prostate cells, we knocked down HSPB8 using pre-designed siRNA and investigated the impact of HSPB8 silencing on cell proliferation, invasion and migration. After siHSPB8 transfection two prostate cancer cell lines showed significantly reduced HSPB8 expression levels (Figures 9A,B). As expected, HSPB8 knockdown substantially increased proliferative rates of two prostate cancer cell lines DU145 and 22Rv1 (Figures 9C,D). Following HSPB8 silencing enhanced invasion and migration abilities were observed in DU145 and 22Rv1 cells (Figures 9E–G). Furthermore, knocking down HSPB8 promoted phosphorylation of AKT and mTOR (Figure 9H).

## 4 Discussion

Identifying molecular markers with potential diagnostic, therapeutic and prognostic values has always been a hotspot in cancer research. In this study we screened out HSPB8 as a potential biomarker from the HSP family using bioinformatic methods, and further investigated its biological role as well as its prognostic significance in prostate cancer.

WGCNA is a broadly-used biological tool for describing interaction patterns between genes among microarray samples by constructing a weighted co-expression network (Langfelder and Horvath, 2008). Compared to unweighted networks, weighted networks could better capture the continuous nature of underlying co-expression information and might therefore avoid an information loss (Langfelder and Horvath, 2008). Using the R package “WGCNA”, we established a co-expression network with a total of 1,485 genes and 550 samples and then implemented hierarchical clustering to group genes with similar expression patterns into the same module. Of the sixteen constructed gene modules, three modules (ME green, ME skyblue and ME black) had HSP genes showing correlation coefficient >0.8 with the corresponding module (HSPBP1 = 0.87 with ME green, HSPA13 = 0.84 with ME skyblue, HSPB8 = 0.81 with ME black). Considering the relatively slight differences in HSPBP1 and HSPA13 expression between normal prostatic tissues and prostate cancer tissues shown in the online tool GEPIA (http://gepia.cancer-pku.cn/) (Accessed date: 16 May 2025), HSPB8 was considered to be the gene of interest for further analysis. Following gene significance (with HSPB8) calculation, the top ten genes ranked by GS. HSPB8 in black module were PGM5, KCNMB1, JPH2, FLNC, MYH11, LMOD1, RASL12, SYNM, ASB2 and CNN1 (excluding HSPB8 itself).

HSPB8, as its name indicates, is a member of HSPBs, which primarily defines whether a HSPBs client will be refolded or degraded (Haslbeck and Vierling, 2015). During tumor development, HSPB8 plays an opposite role—pro-tumoral or anti-tumoral—depending on the tumor type (Cristofani et al., 2021). For example, HSPB8 promotes tumor growth in breast cancer (Piccolella et al., 2017), lung cancer (Yu et al., 2021), ovarian cancer (Suzuki et al., 2015), and gastric cancer (Li et al., 2014), while in other tumor types, such as hepatocarcinoma (Matsushima-Nishiwaki et al., 2017) and prostate cancer (Gober et al., 2003), HSPB8 could repress tumorigenesis. Our findings—based on analyses using TCGA database and the R package “limma”, as well as the online tool GEPIA (http://gepia.cancer-pku.cn/) (Accessed date: 16 May 2025) — showed that compared to normal prostatic samples, lower HSPB8 expression was observed in prostate cancer tissues. This is in line with existing data (Gober et al., 2003; Yao et al., 2019; Kim et al., 2020) and indicates that HSPB8 functions as a tumor suppressor gene during the initiation and progression of prostate cancer. Ten genes mentioned in the last paragraph, i.e., PGM5, KCNMB1, JPH2, FLNC, MYH11, LMOD1, RASL12, SYNM, ASB2 and CNN1, had relatively higher GS. HSPB8 values compared with others. As expected, these genes positively correlated with HSPB8 expression (p < 0.001), suggesting that they might be the downstream target or upstream regulator of HSPB8. This speculation needed to be supported by more mechanistic studies.

We further performed GO and KEGG functional enrichment analyses on HSPB8 and related genes. The criteria for HSPB8-related genes was GS. HSPB8 > 0.6, and a total of 911 genes were selected as our target subjects. In GO functional annotation, these genes were primarily enriched in muscle system process, collagen−containing extracellular matrix, muscle cell differentiation and muscle contraction, while in KEGG analysis cytoskeleton in muscle cells, calcium signaling pathway, and PI3K−AKT signaling pathway were top three enriched KEGG pathways. This indicated that our targets probably had close relationship with smooth muscle contraction. Further validation through single-cell analysis also supported this finding. We used online tool TISCH2 (http://tisch.compbio.cn/home) (Accessed date: 15 June 2025) and attached single-cell datasets of prostate cancer (GSE_137829, GSE_141445, GSE_172301 and GSE_176031) to detect the gene distribution of HSPB8 within the prostate gland. Our data demonstrated that HSPB8 was primarily expressed in fibroblasts and epithelial cells. This finding aligned with gene functional annotations, providing evidence for HSPB8’s important role in tumor stroma.

To verify whether HSPB8 and top genes PGM5, KCNMB1, JPH2, FLNC, MYH11, LMOD1, RASL12, SYNM, ASB2 and CNN1 were survival-related genes, we performed cox regression and survival analysis on them using the R package “survival”. Their hazard ratios were less than 1 (p < 0.001) and lower expression levels were observed among patient groups with lower survival probability (p < 0.05), indicating that lack of those genes often implied poor survival and they were probably tumor suppressor genes on the other hand. We further associated HSPB8 with patients’ survival outcomes from the immunological prospective. By employing the ESTIMATE algorism, we investigated the association of HSPB8 with stromal and immune components in TIME based on obtained stromal score, immune score and ESTIMATE score of prostate cancer samples. Stromal score and immune score, as their names indicate, are powerful tools for assessing the infiltration of stromal and immune cells (Yoshihara et al., 2013). Our survival data illustrated that poorer survival was observed among patients with high stromal, immune and ESTIMATE scores. This was in accordance with a previously-reported finding that high stromal scores were associated with higher Gleason scores, an increased risk of tumor metastasis, and poorer clinical outcomes (Mahal et al., 2020). High immune scores in patients with prostate cancer might result from abundant infiltration of immunosuppressive cells, such as T regulatory cells (Tregs) and M2-polarized macrophages (Shen et al., 2021), which was linked to unfavorable survival outcomes (Andersen et al., 2021). It was interesting to note that across all three score metrics HSPB8 was significantly higher in high-score group than in low-score group, with groups stratified by the median threshold. This finding stands in contrast to our previous results reporting that HSPB8 is a tumor suppressor gene and, to the best of our knowledge, no studies to date have reported such a contradiction. Further immunological experiments are required to validate this observation.

Then, we established a prognostic model using genes in black module with GS. HSPB8 > 0.9 using LASSO regression method. The risk score could be generated via the following formula: Risk score = (−0.5882)*AC005180.2 + (−0.1387)*LDB3 + (0.1349)*BHMT2 + (0.2432)*TAGLN. Upon the division of patients into high-risk and low-risk patient groups, we found that the high-risk patient group had poorer survival prospect compared to the low-risk group (p = 0.002), demonstrating this is a risk model. In addition, the AUC values for this model at the time point 1 year, 3 years and 5 years were 0.746 (95% CI: 0.623–0.868), 0.749 (95% CI: 0.656–0.843) and 0.684 (95% CI: 0.579–0.790), respectively. All the evidence indicated the validity of this model.

Characterizing the correlation between HSPB8 and various clinical phenotypes is also an important objective of this research. We compared expression differences of HSPB8 among different patient groups and clinical phenotypes we focused on included age, tumor T and N stage, Gleason score and PSA. In this study, no statistically significant differences in HSPB8 expression were observed between patients aged ≦ 60 years and >60 years. Regarding tumor T and N stages, HSPB8 expression was significantly higher in T2 groups than in T3 group, and lower in N1 groups than in N0 groups. Patients had been divided into three groups based on their PSA levels: <4 ng/mL, 4–10 ng/mL and >10 ng/mL, and HSPB8 expression was lower in patients of >10 ng/mL than in those of <4 ng/mL. For patients’ Gleason score, the group of Gleason score = 6 showed significantly higher HSPB8 expression levels than those of Gleason score = 9 and = 10. All such results supported a strong association between HSPB8 expression and the severity of prostate cancer, with lower HSPB8 expression generally observed among patients exhibiting more pronounced abnormalities in prostate cancer-related parameters.

As a final step, we carried out gene expression validation using prostate cancer cell lines DU145 and 22Rv1. After knocking down HSPB8 using pre-designed siRNA, we found that lack of HSPB8 stimulated cellular proliferation of DU145 and 22Rv1 cell lines. HSPB8-silencing prostate cell lines also showed enhanced invasion and migration ability. These results strongly supported the inhibitory role of HSPB8 in the development and progression of prostate cancer. Further, we carried out an in-depth investigation into the molecular mechanisms underlying this regulation. PI3K−AKT axis is a classical cancer-relevant signaling pathway and plays an oncogenic role in many cancer types, such as gastric cancer (Fattahi et al., 2020), breast cancer (Miricescu et al., 2020) and prostate cancer (Edlind and Hsieh, 2014). Since PI3K−AKT signaling was identified as the third most enriched KEGG pathway in gene enrichment analysis, we speculated that it might be linked to HSPB8’s tumor-suppressive role in prostate cancer. Our mechanistic studies revealed that HSPB8 knockdown promoted phosphorylation of AKT and mTOR, which aligned with our speculation, i.e., the inhibitory role of HSPB8 in prostate cancer was, at least in part, linked to the inactivation of PI3K−AKT signaling pathway.

## 5 Conclusion

In this study we identified HSPB8 as new biomarker with potential diagnostic, therapeutic and prognostic values in prostate cancer through WGCNA. HSPB8 was a tumor suppressor gene and had significant impact on patients’ survival and prognosis. On a molecular level, its functional role was probably mediated via inactivation of PI3K−AKT signaling. In aggregate, this study provided a novel insight into the pathogenesis of prostate cancer and targeting HSPB8 appeared to be an emerging area in prostate cancer treatment.

## Data Availability

The original contributions presented in the study are included in the article/supplementary material, further inquiries can be directed to the corresponding author.
