# Supplementary figures and images for: Recognition of HSPB8 as a potential therapeutic target for prostate cancer
Source: Front Genet. 2025 Oct 21;16:1680674. doi: 10.3389/fgene.2025.1680674 (PMC12582549; doi:10.3389/fgene.2025.1680674)

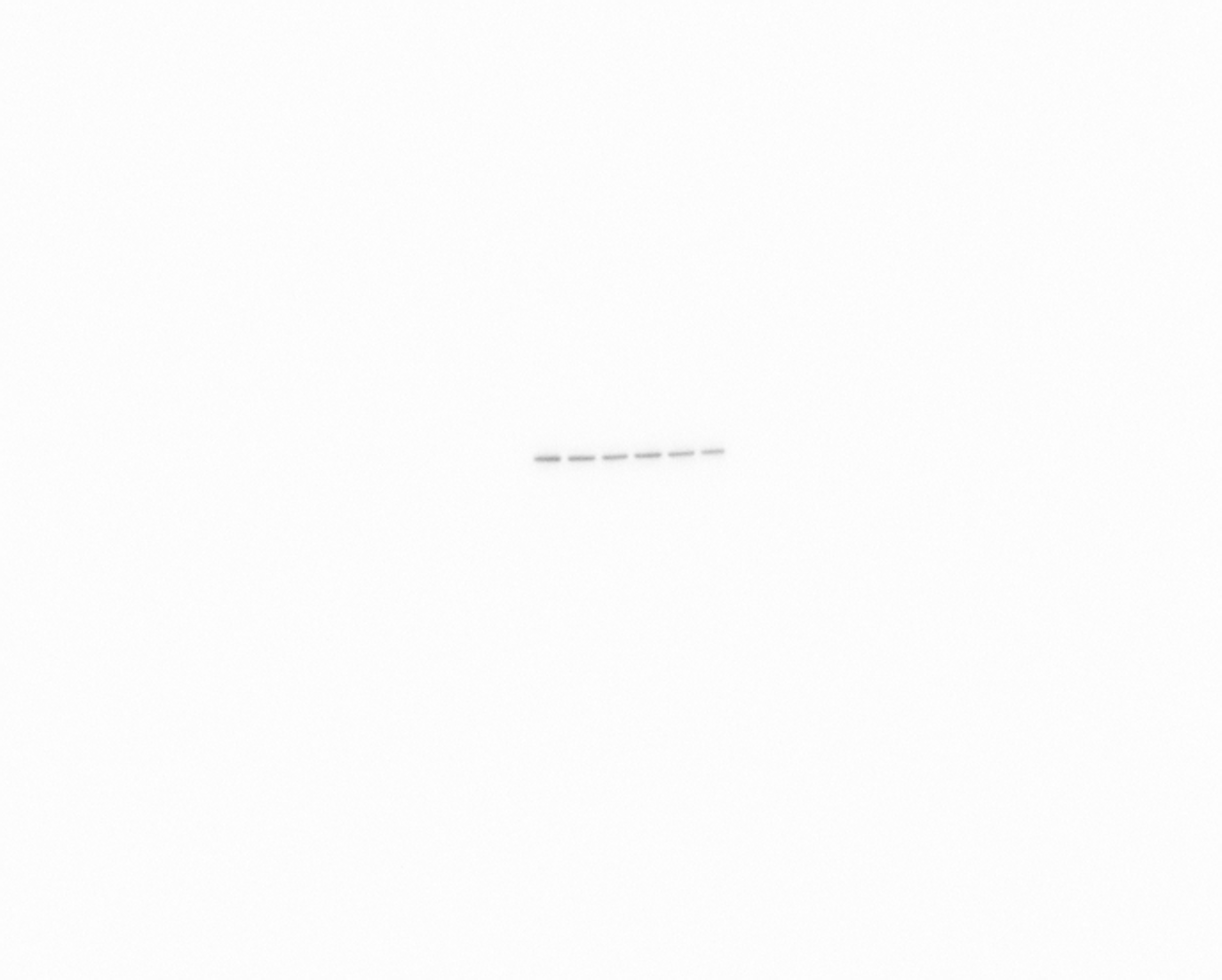

Supplement: Supplementary file 1 [file DataSheet1.zip › WB/ACTIN/1.Tif]

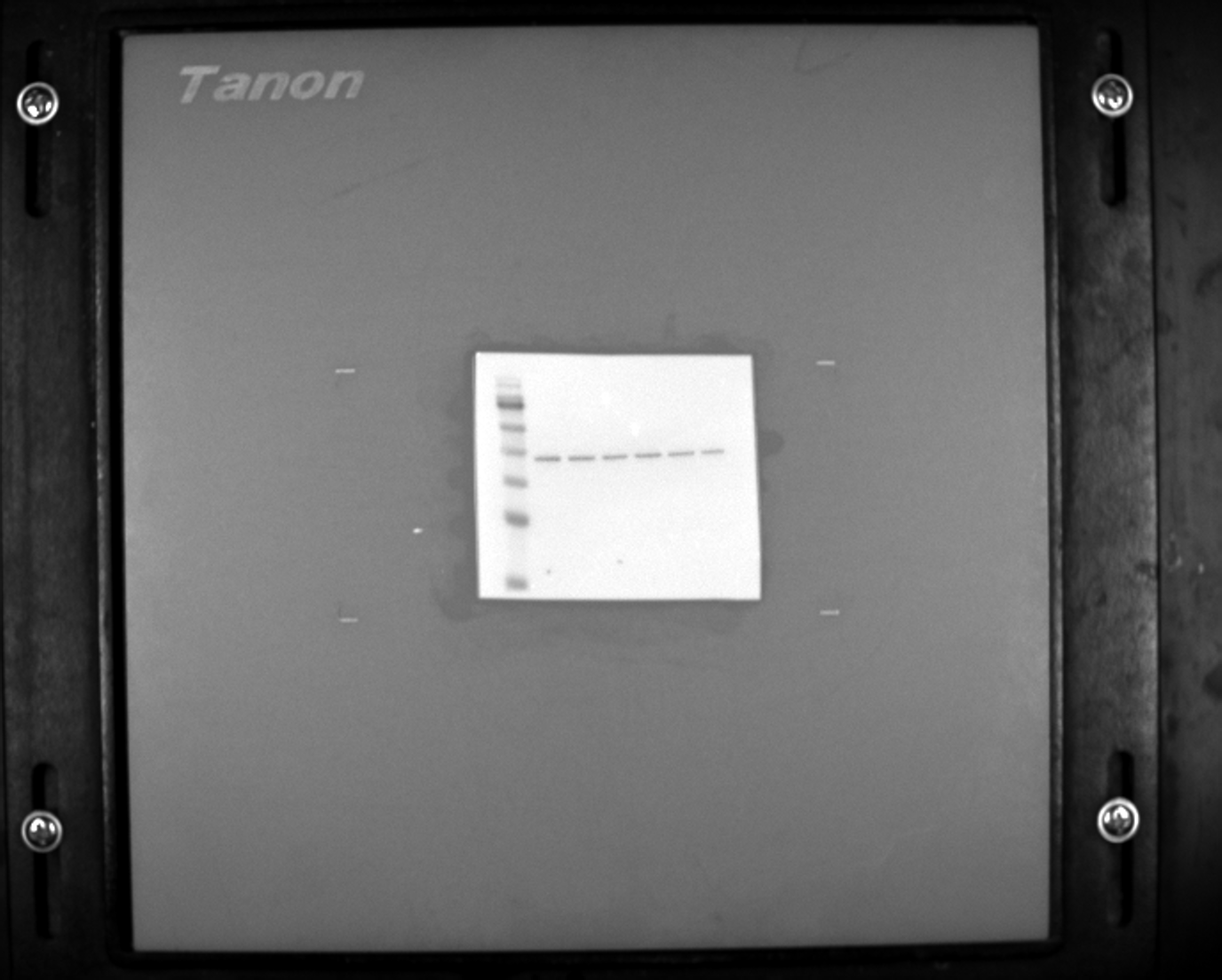

Supplement: Supplementary file 1 [file DataSheet1.zip › WB/ACTIN/1M.Tif]

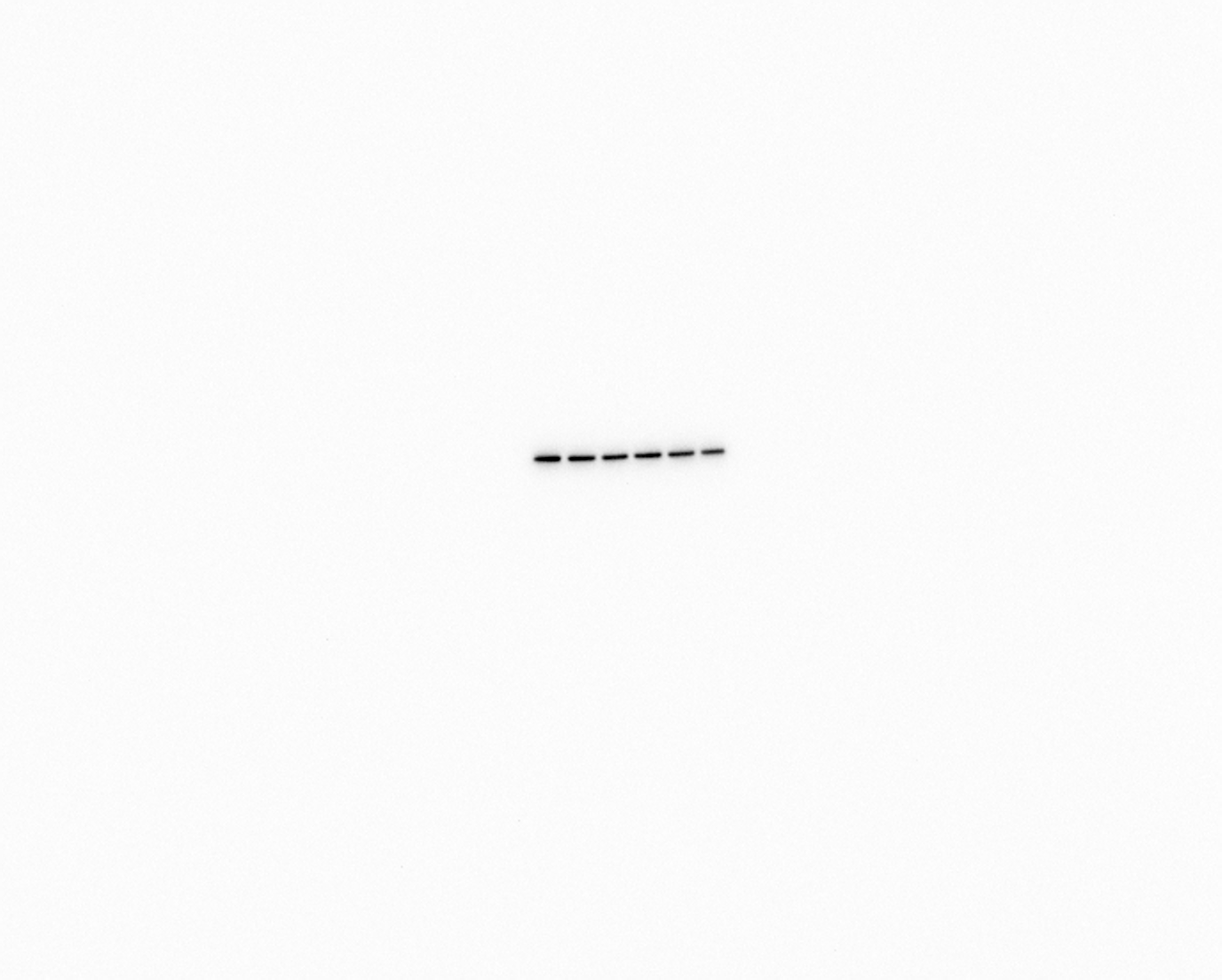

Supplement: Supplementary file 1 [file DataSheet1.zip › WB/ACTIN/2.Tif]

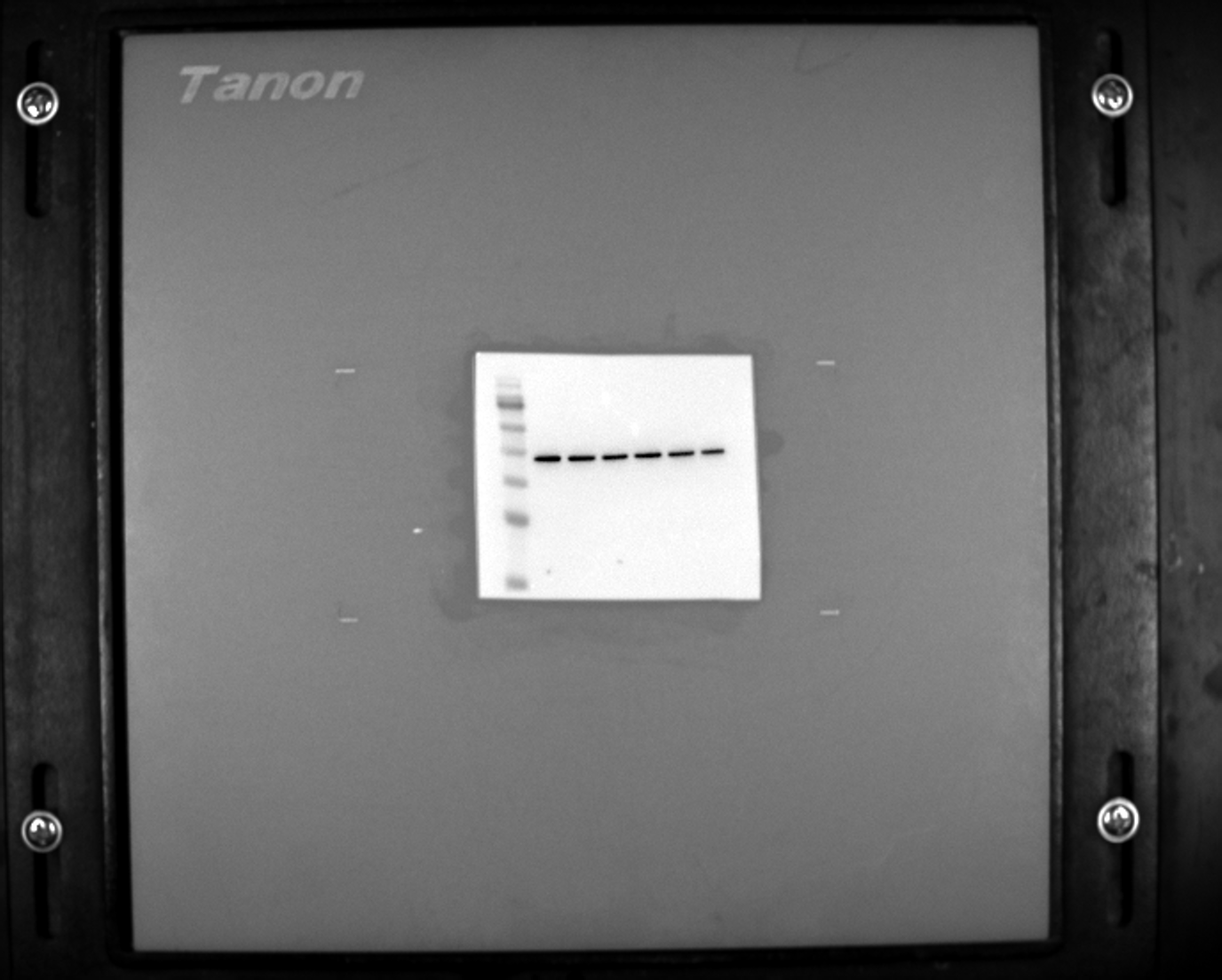

Supplement: Supplementary file 1 [file DataSheet1.zip › WB/ACTIN/2M.Tif]

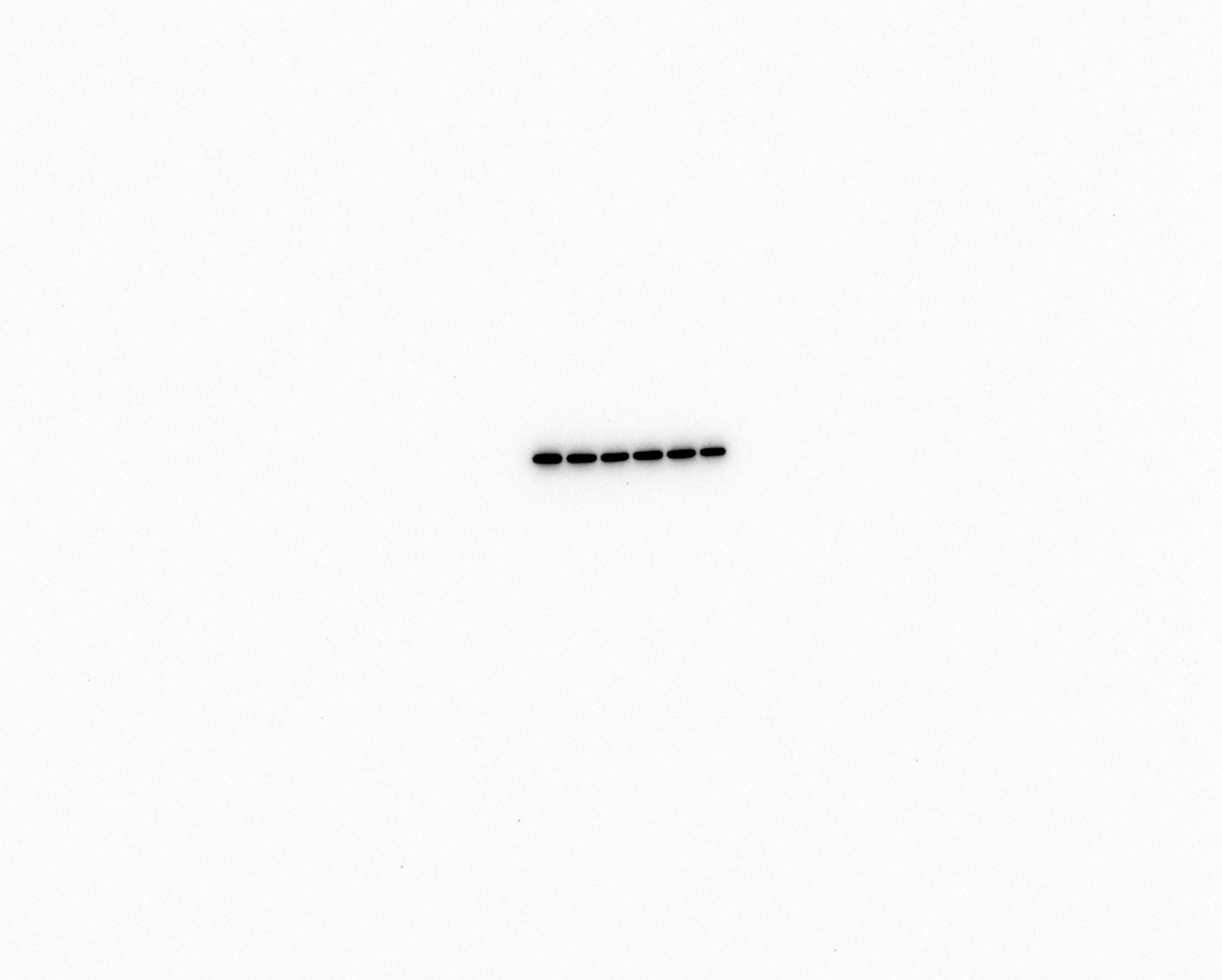

Supplement: Supplementary file 1 [file DataSheet1.zip › WB/ACTIN/3.Tif]

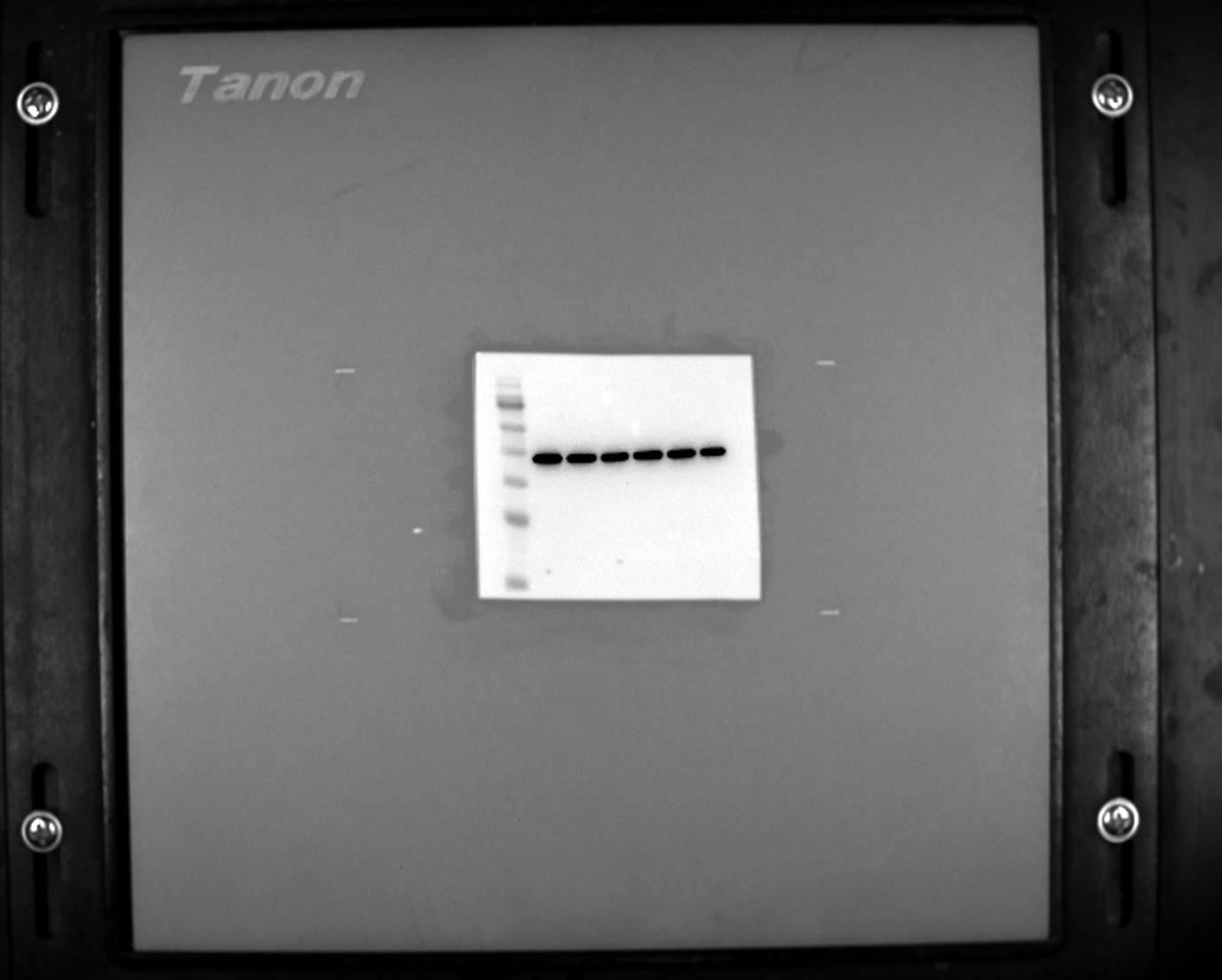

Supplement: Supplementary file 1 [file DataSheet1.zip › WB/ACTIN/3M.Tif]

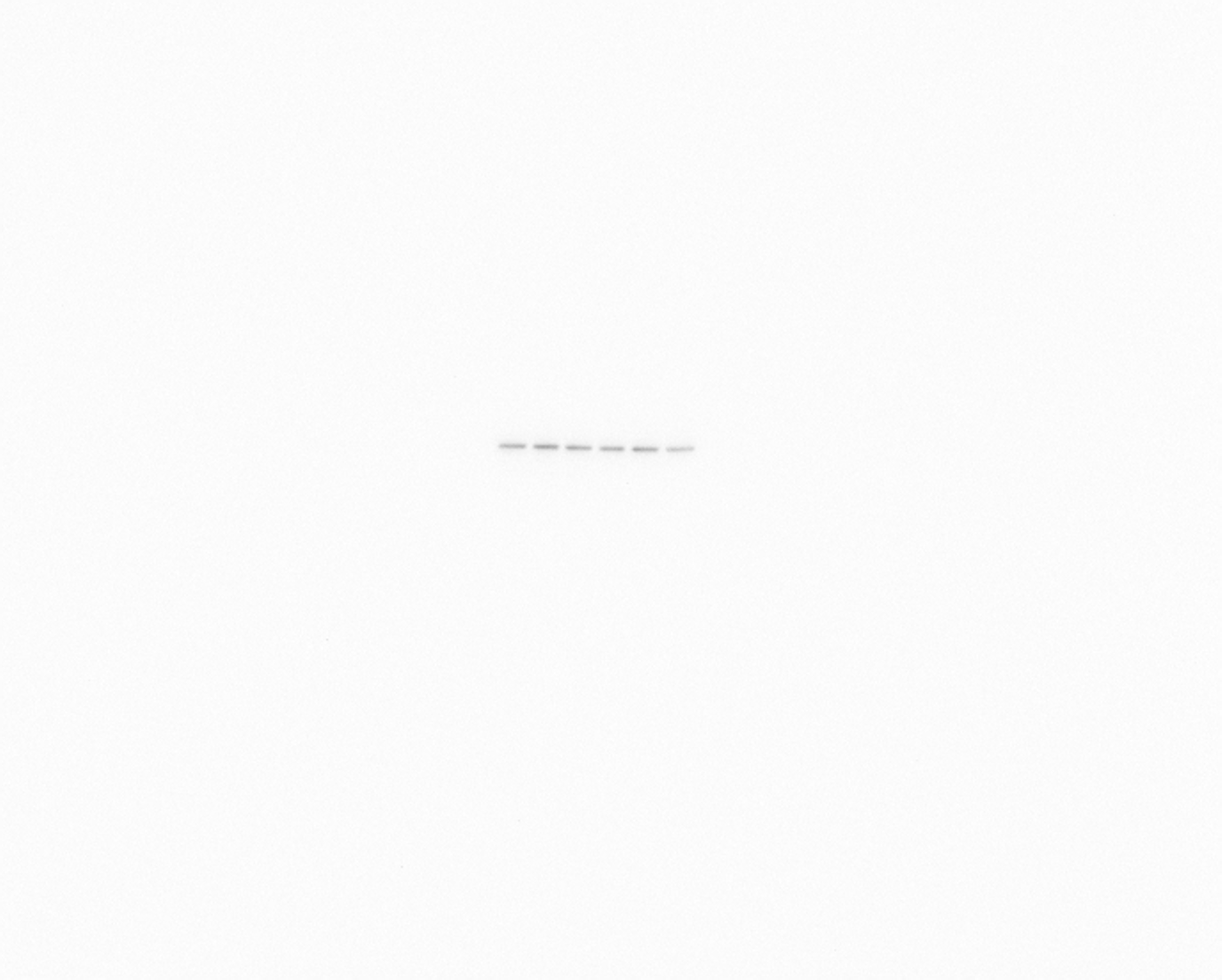

Supplement: Supplementary file 1 [file DataSheet1.zip › WB/AKT/1.Tif]

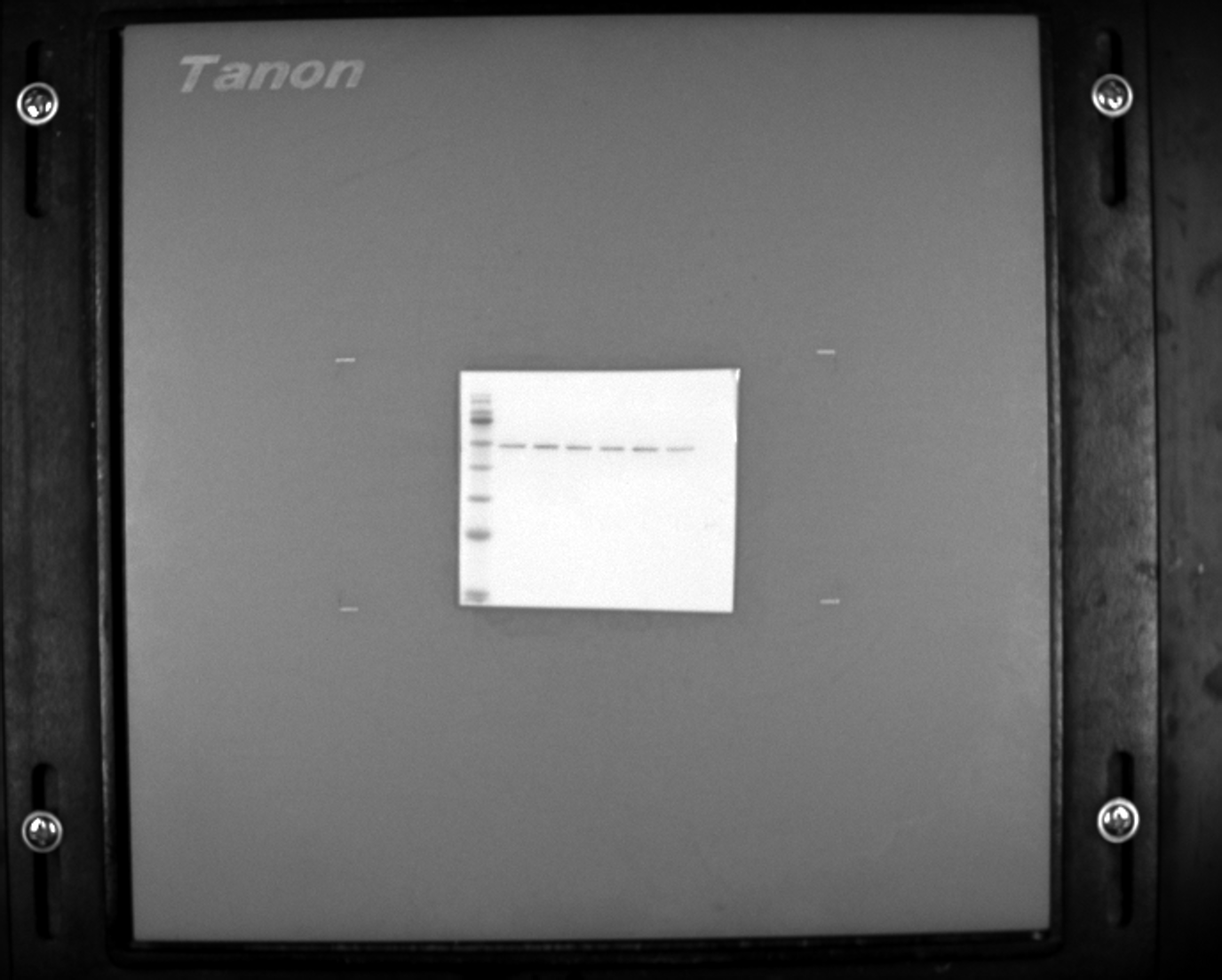

Supplement: Supplementary file 1 [file DataSheet1.zip › WB/AKT/1M.Tif]

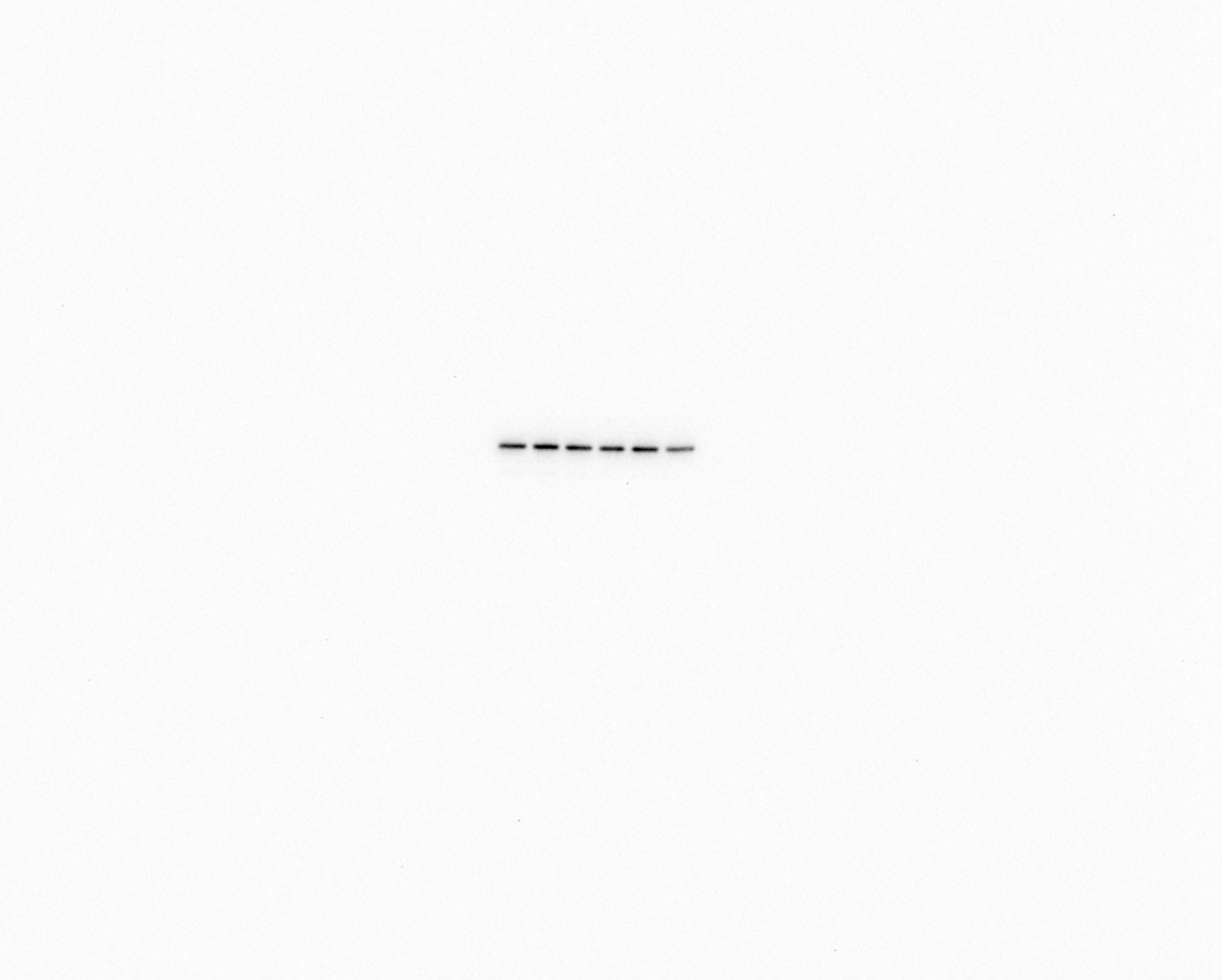

Supplement: Supplementary file 1 [file DataSheet1.zip › WB/AKT/2.Tif]

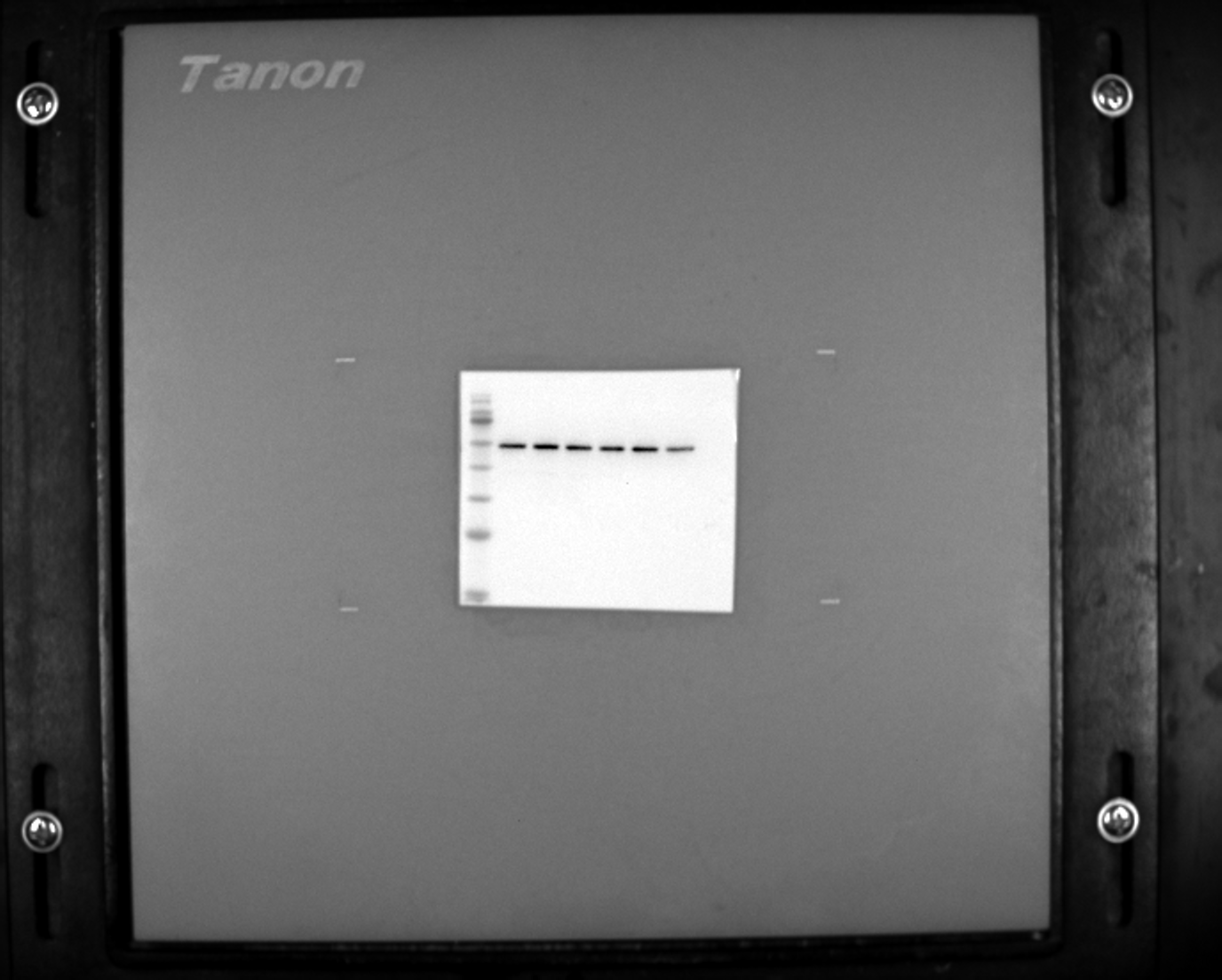

Supplement: Supplementary file 1 [file DataSheet1.zip › WB/AKT/2M.Tif]

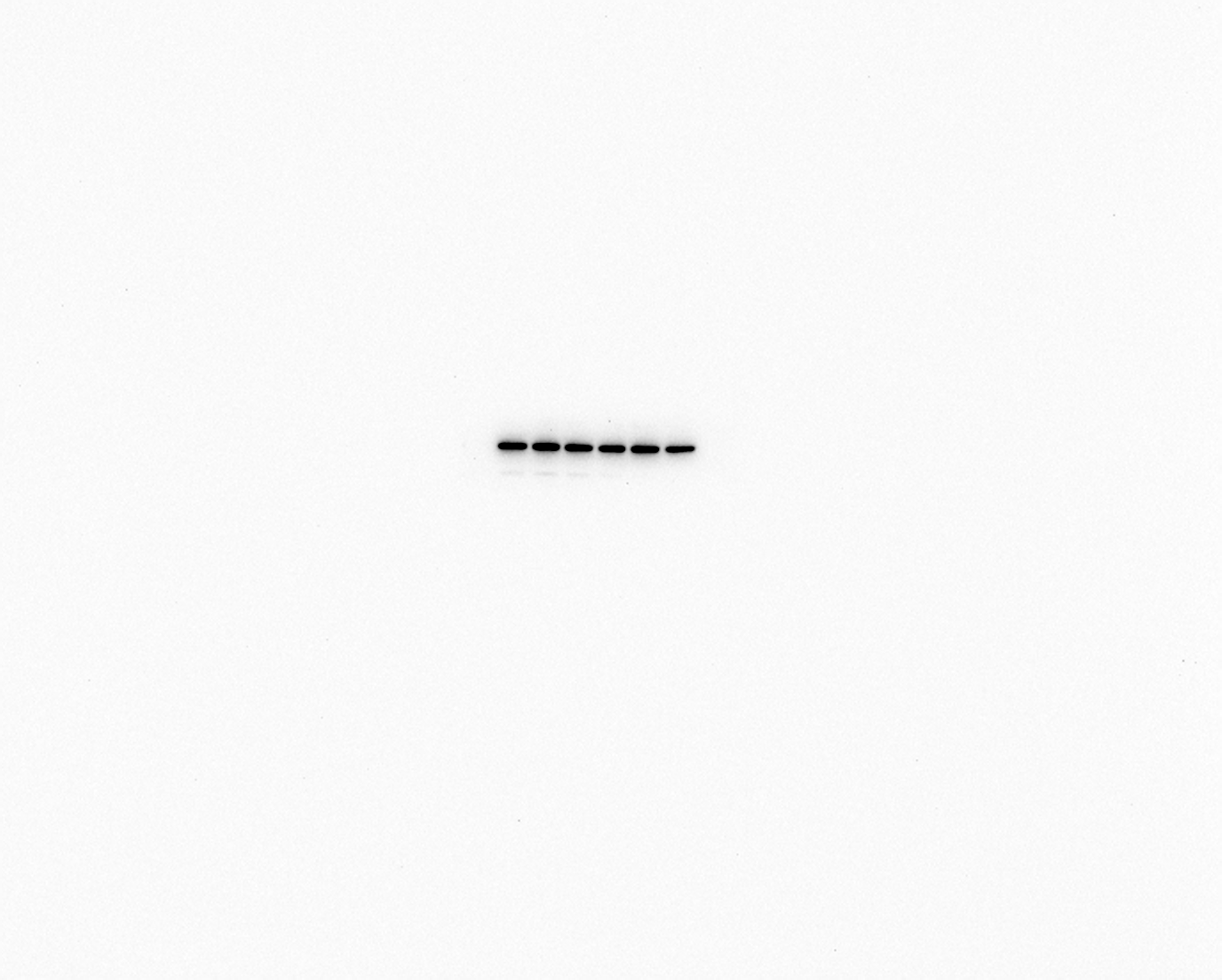

Supplement: Supplementary file 1 [file DataSheet1.zip › WB/AKT/3.Tif]

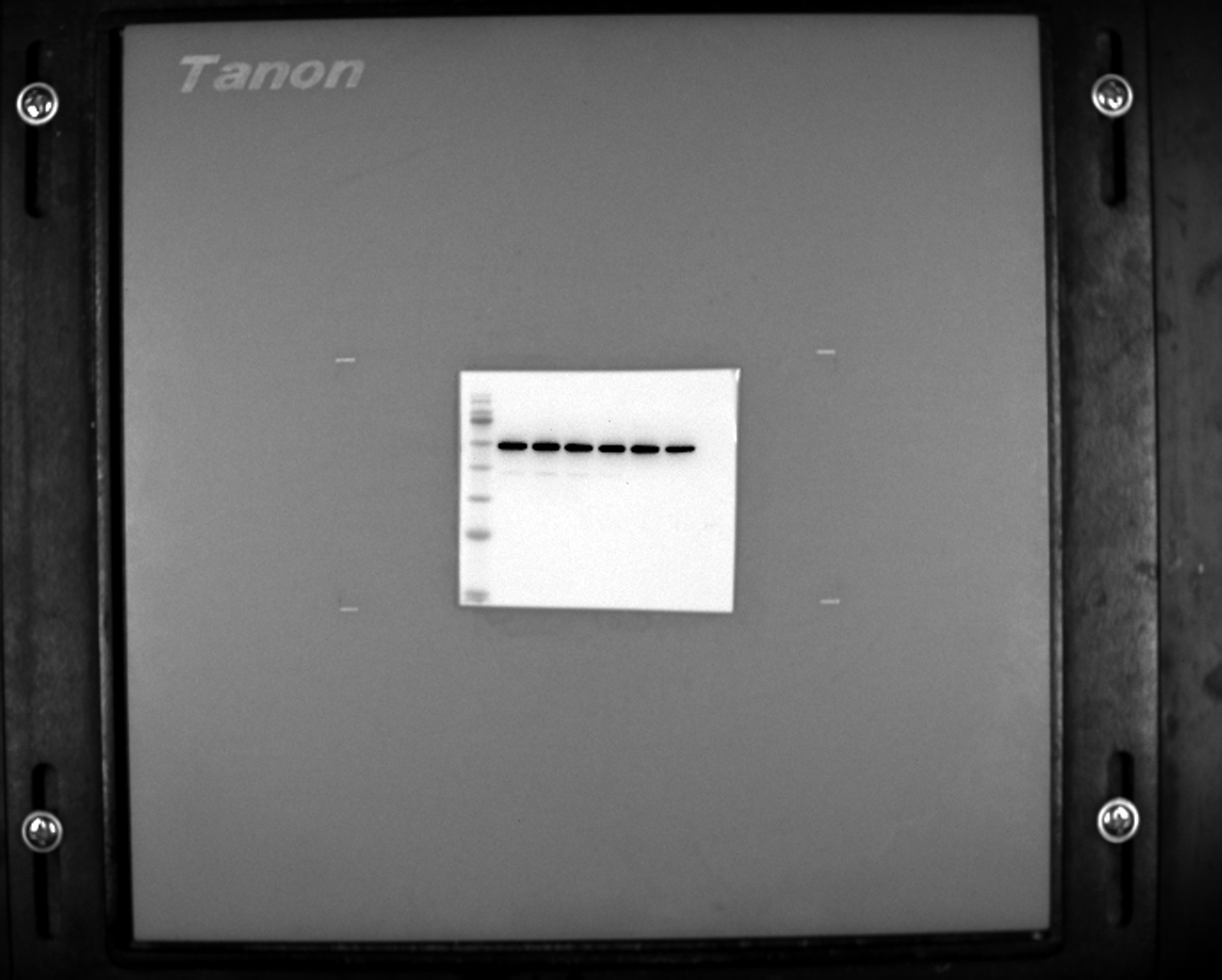

Supplement: Supplementary file 1 [file DataSheet1.zip › WB/AKT/3M.Tif]

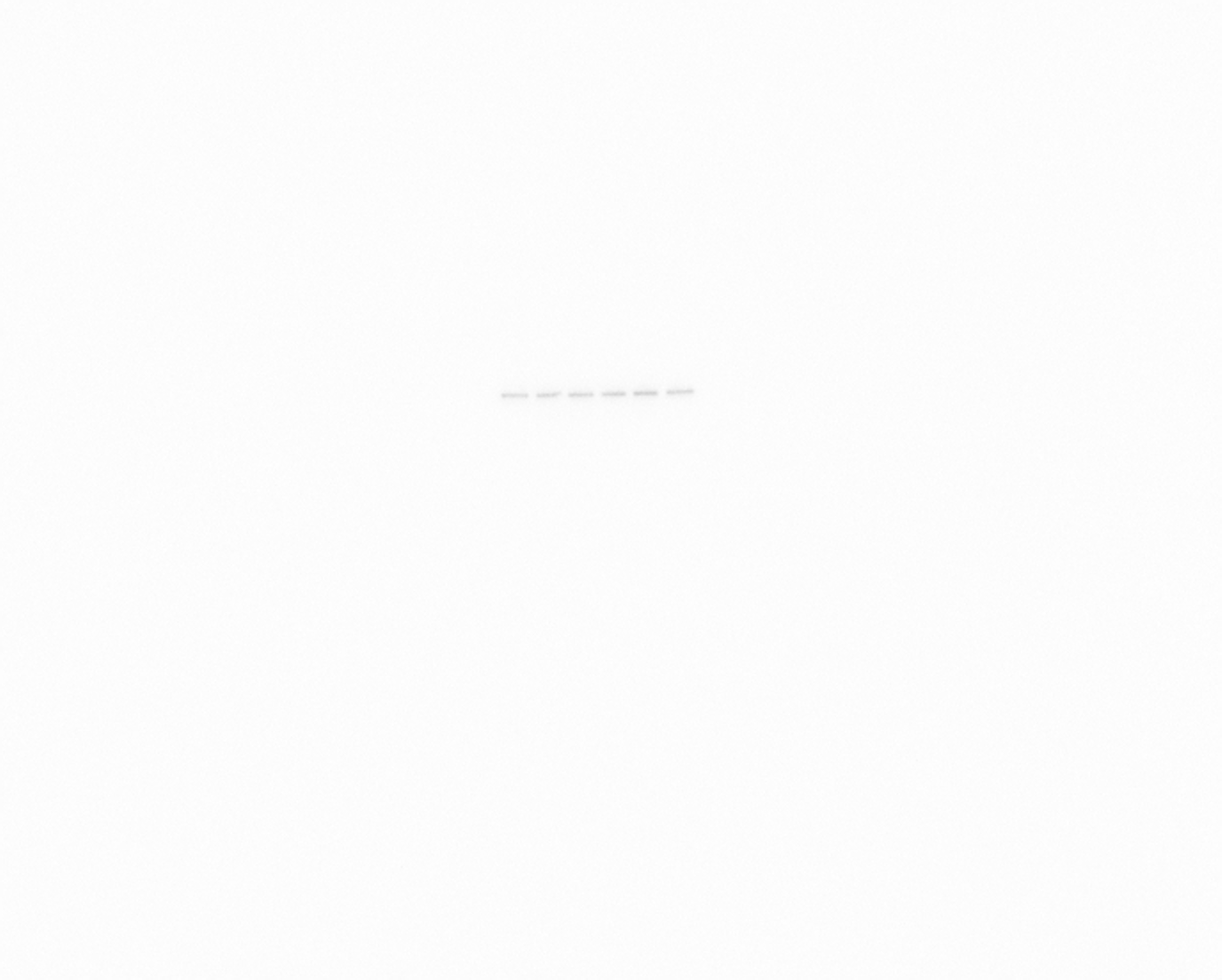

Supplement: Supplementary file 1 [file DataSheet1.zip › WB/mTOR/1.Tif]

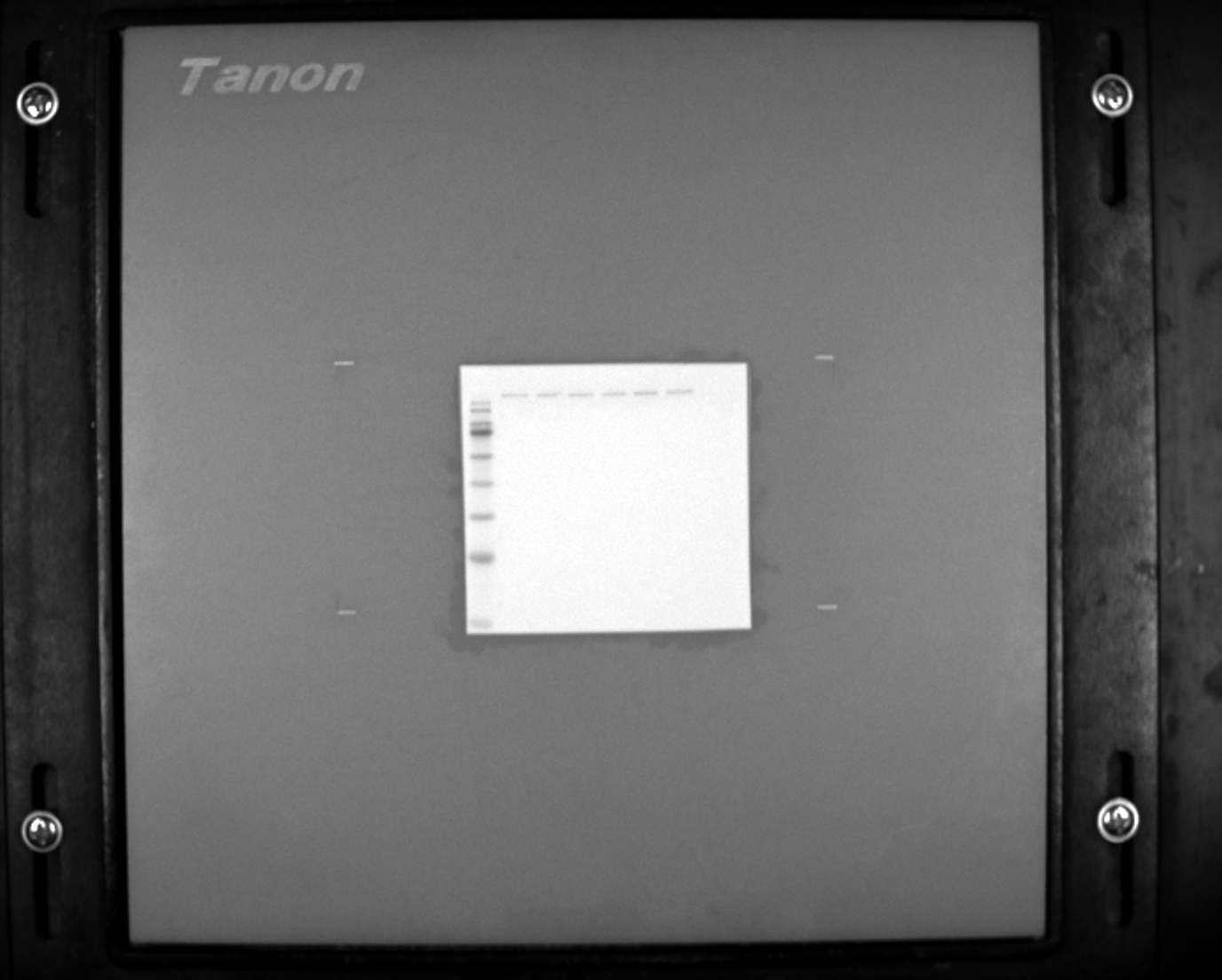

Supplement: Supplementary file 1 [file DataSheet1.zip › WB/mTOR/1M.Tif]

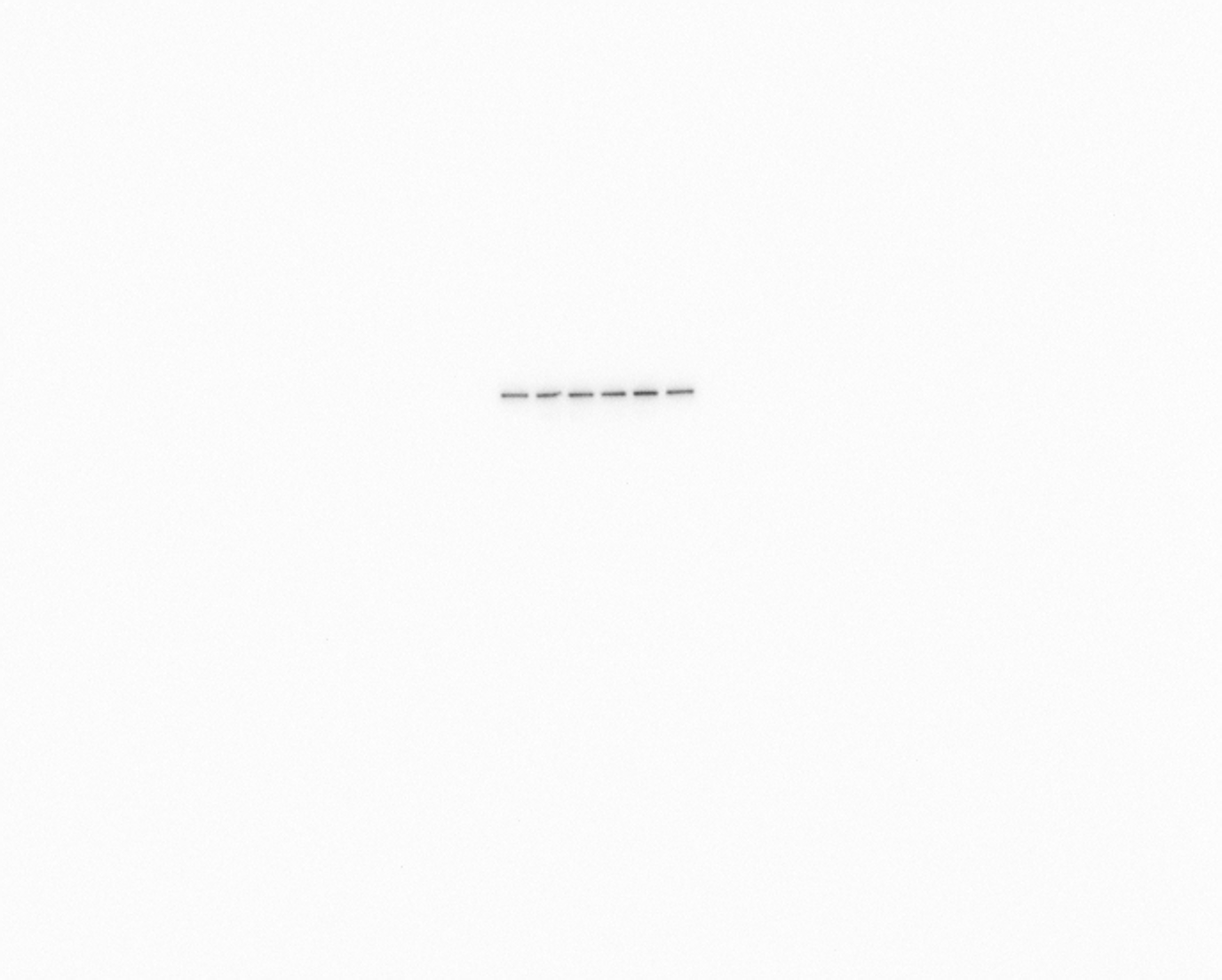

Supplement: Supplementary file 1 [file DataSheet1.zip › WB/mTOR/2.Tif]

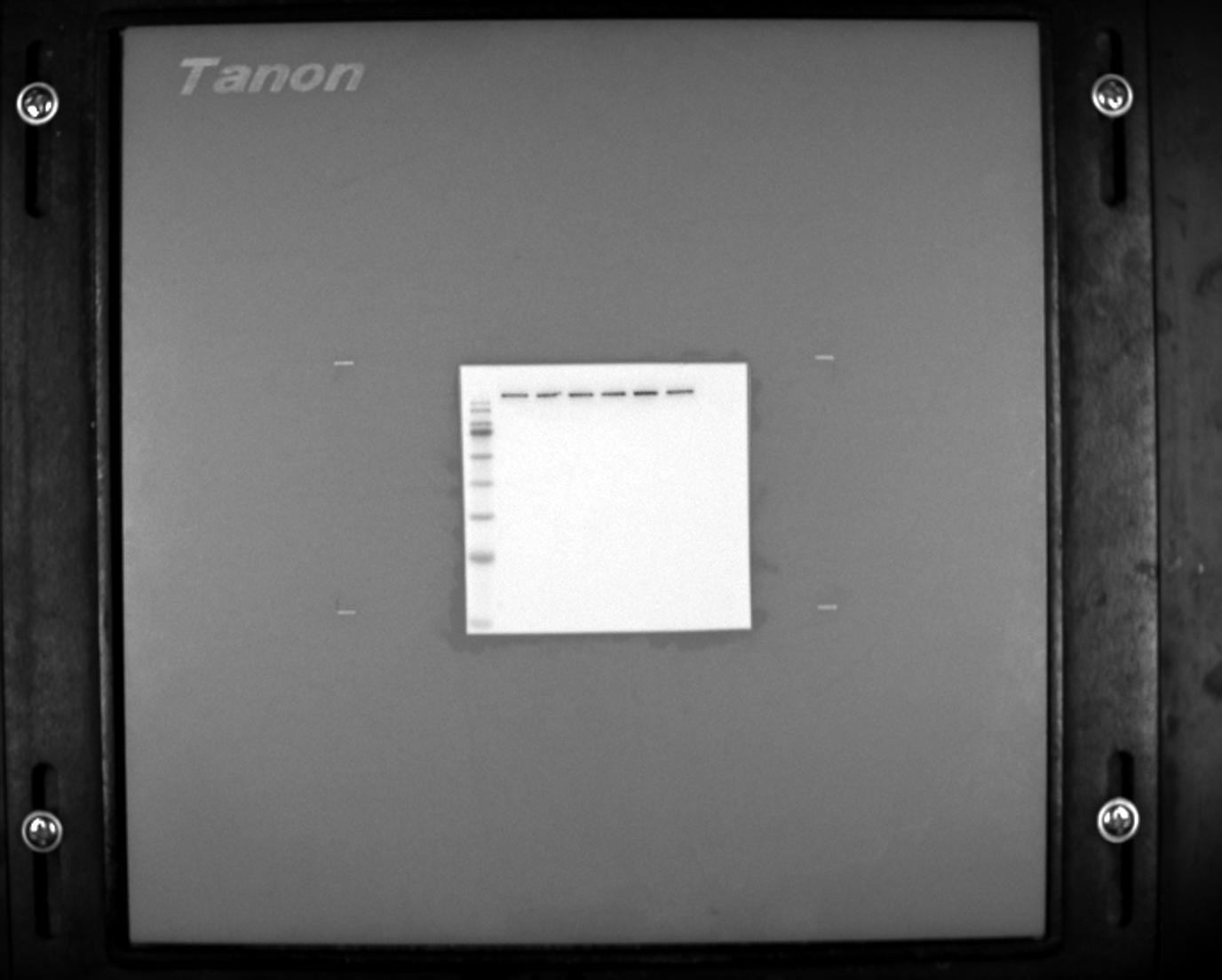

Supplement: Supplementary file 1 [file DataSheet1.zip › WB/mTOR/2M.Tif]

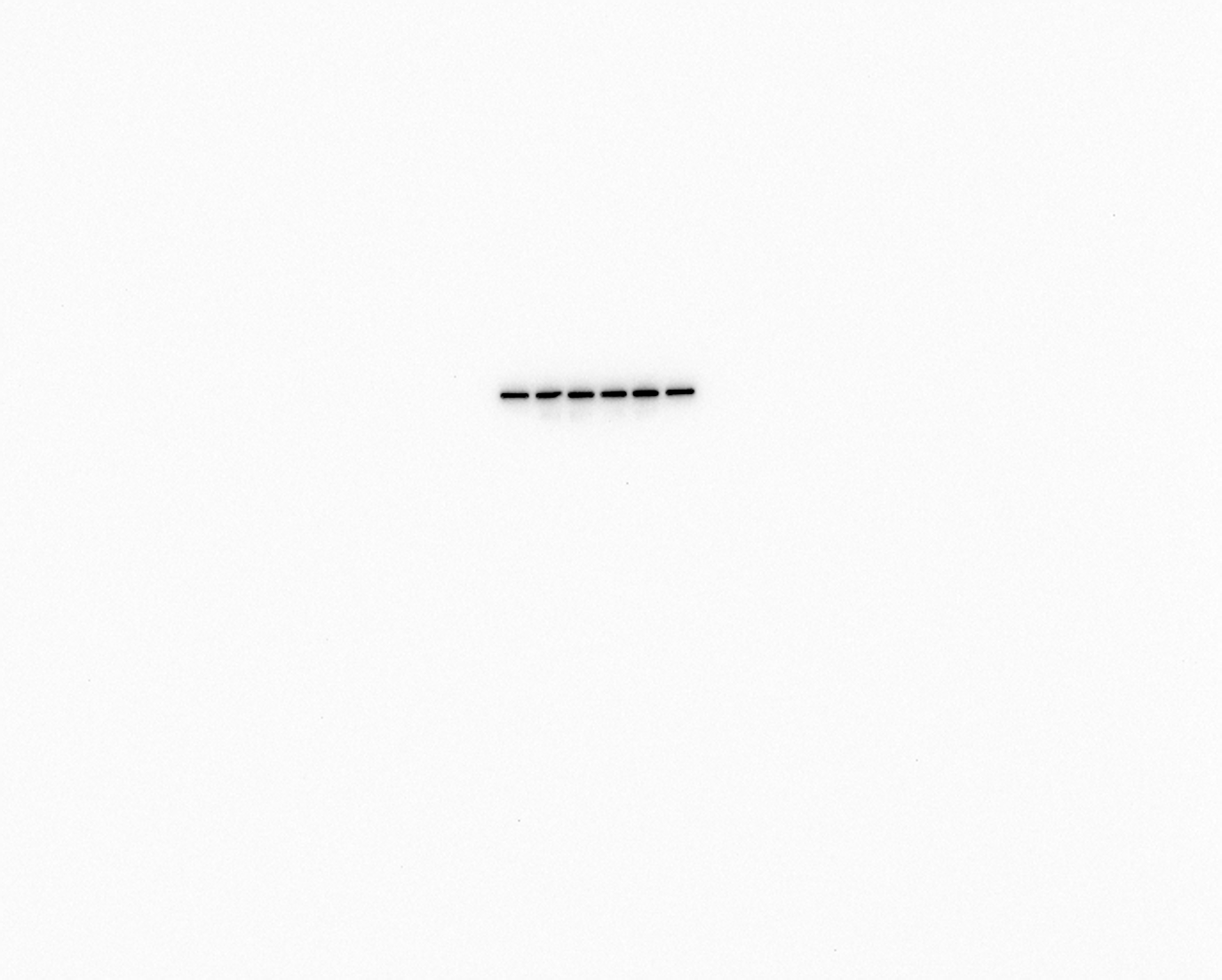

Supplement: Supplementary file 1 [file DataSheet1.zip › WB/mTOR/3.Tif]

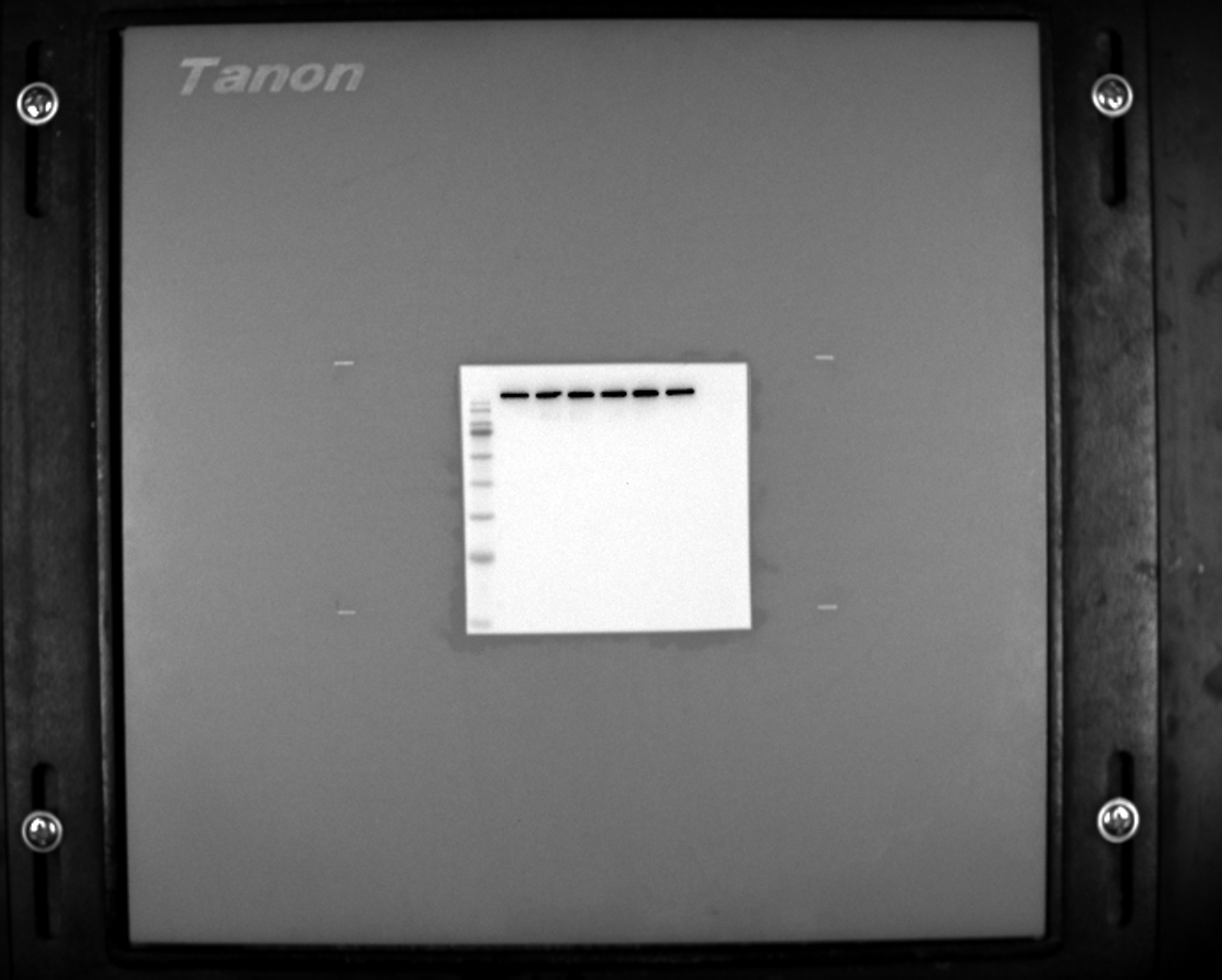

Supplement: Supplementary file 1 [file DataSheet1.zip › WB/mTOR/3M.Tif]

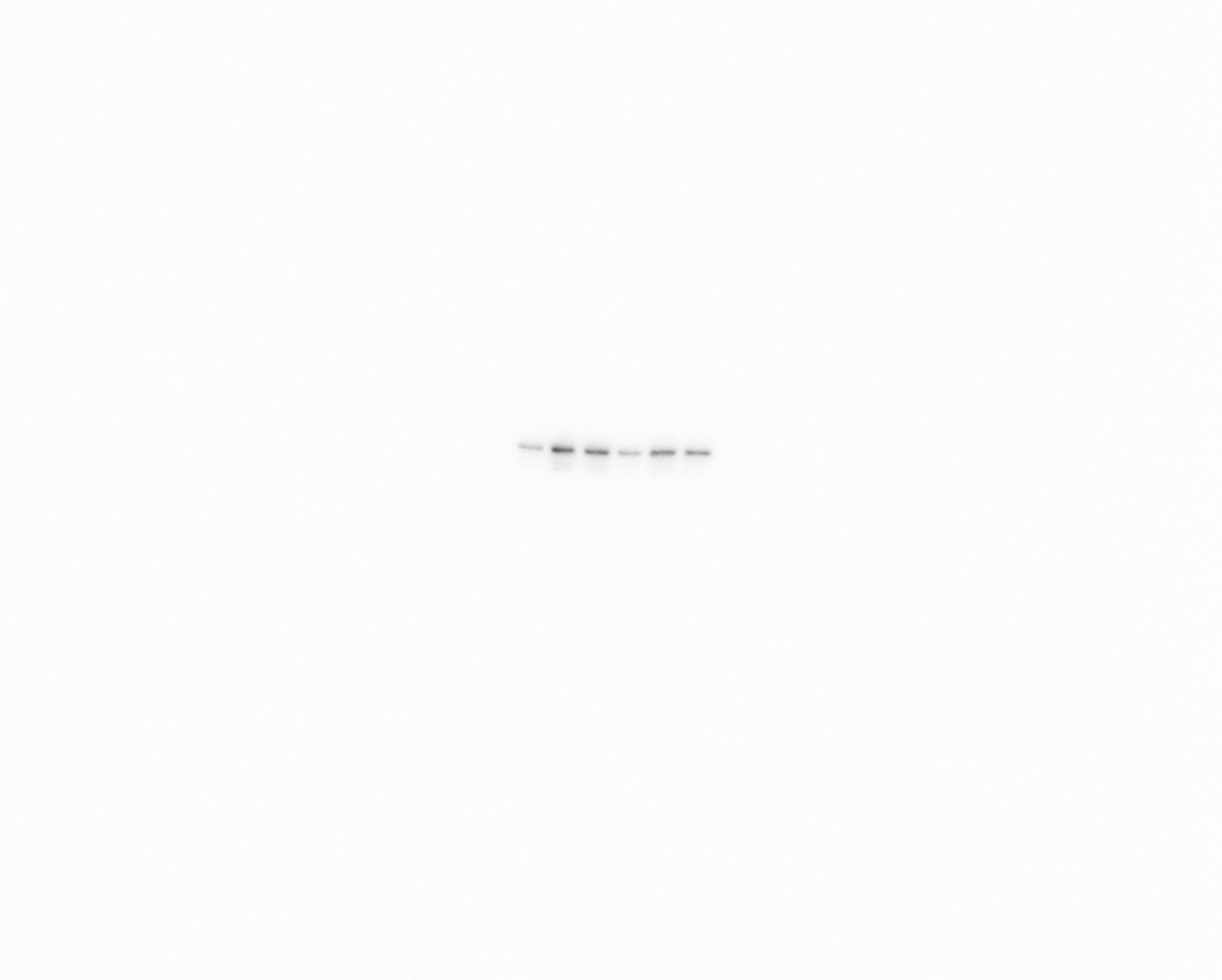

Supplement: Supplementary file 1 [file DataSheet1.zip › WB/p-AKT/1.Tif]

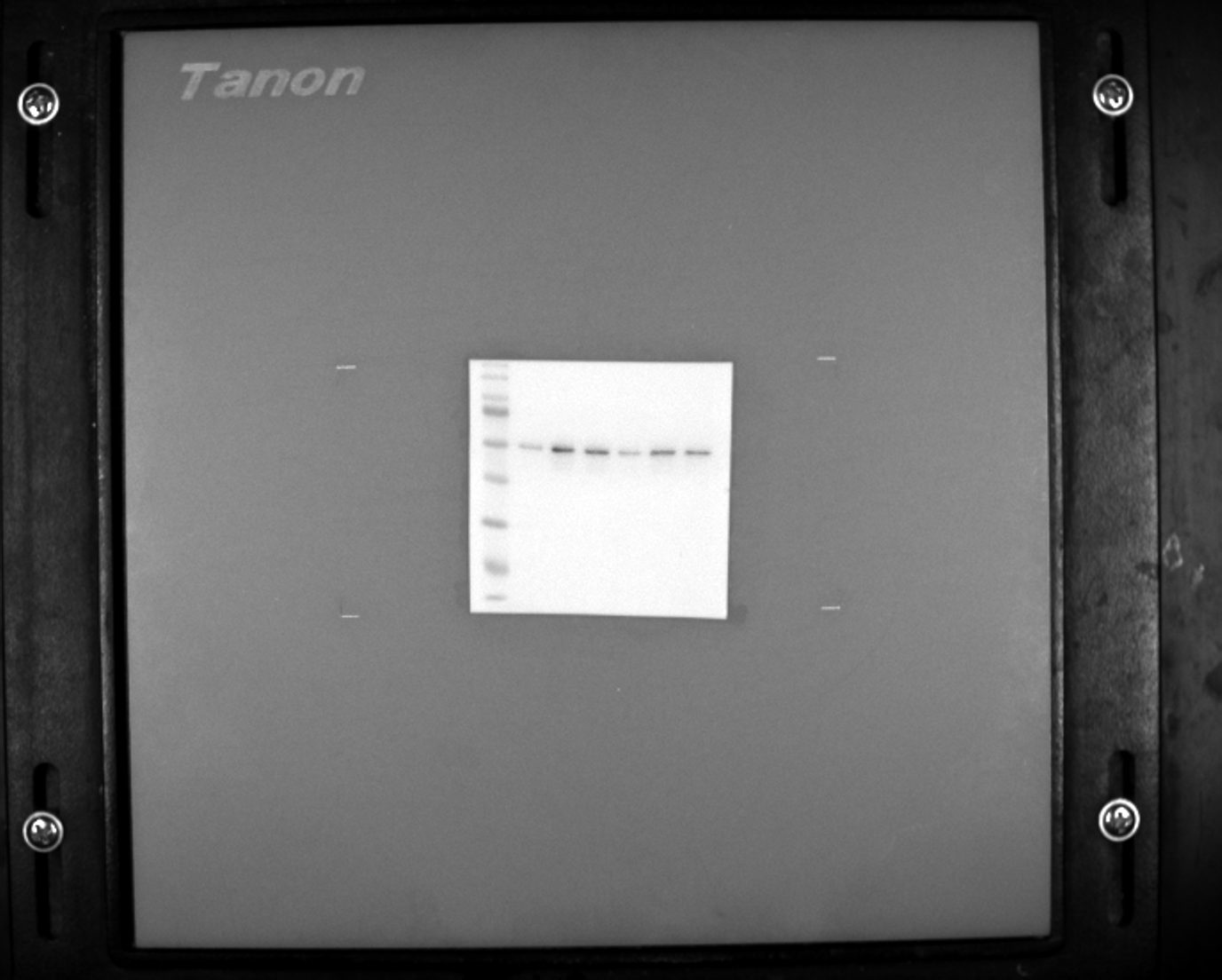

Supplement: Supplementary file 1 [file DataSheet1.zip › WB/p-AKT/1M.Tif]

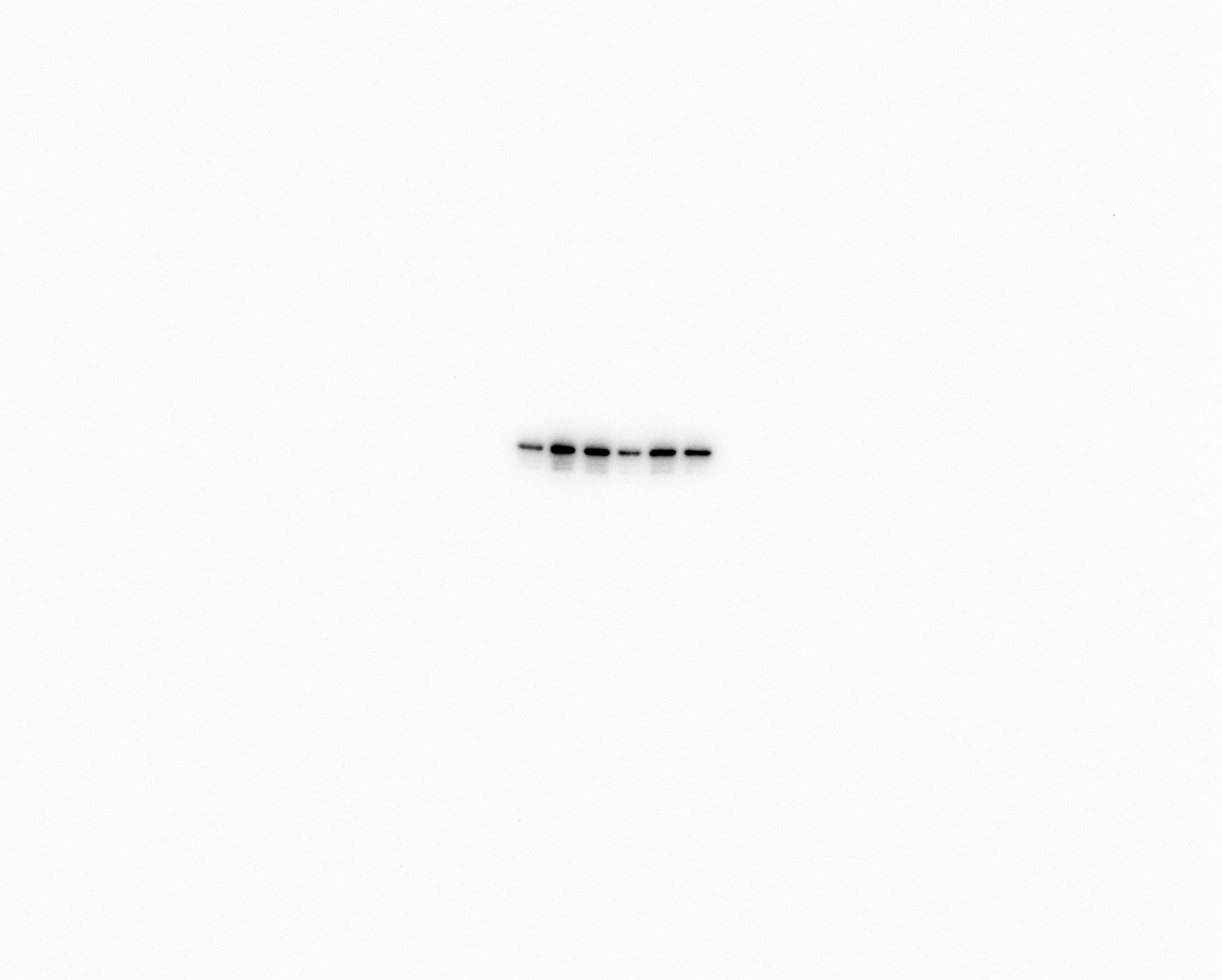

Supplement: Supplementary file 1 [file DataSheet1.zip › WB/p-AKT/2.Tif]

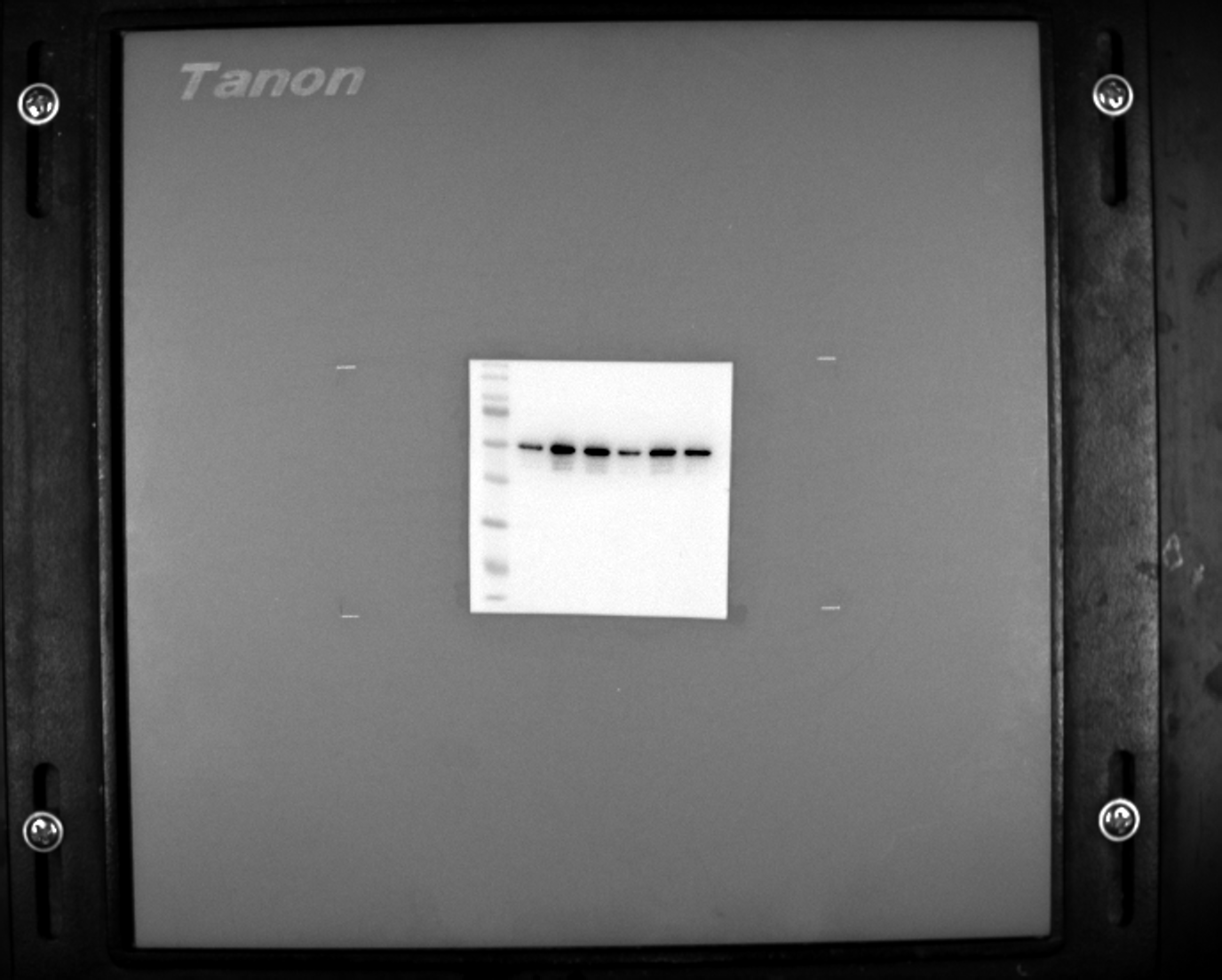

Supplement: Supplementary file 1 [file DataSheet1.zip › WB/p-AKT/2M.Tif]

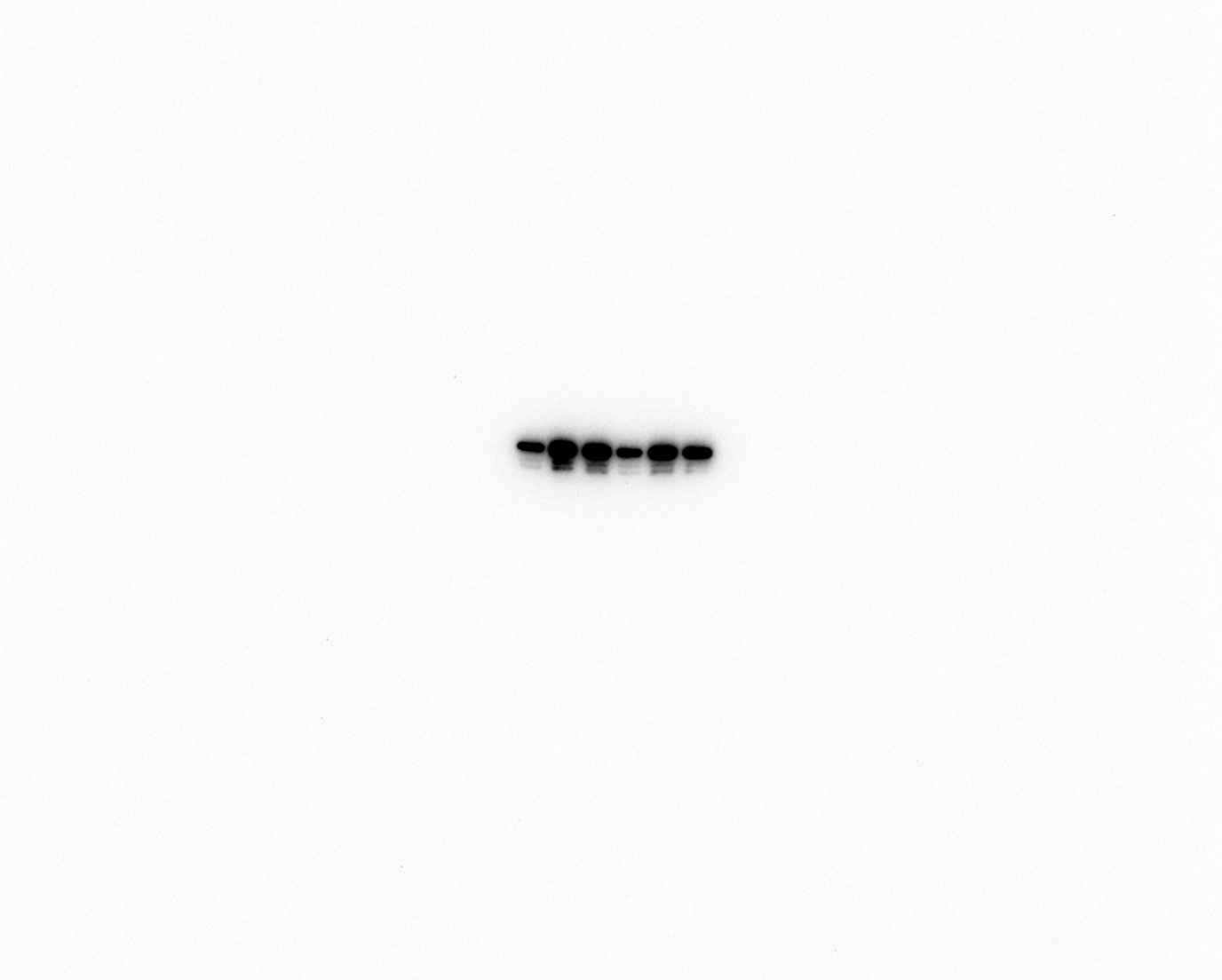

Supplement: Supplementary file 1 [file DataSheet1.zip › WB/p-AKT/3.Tif]

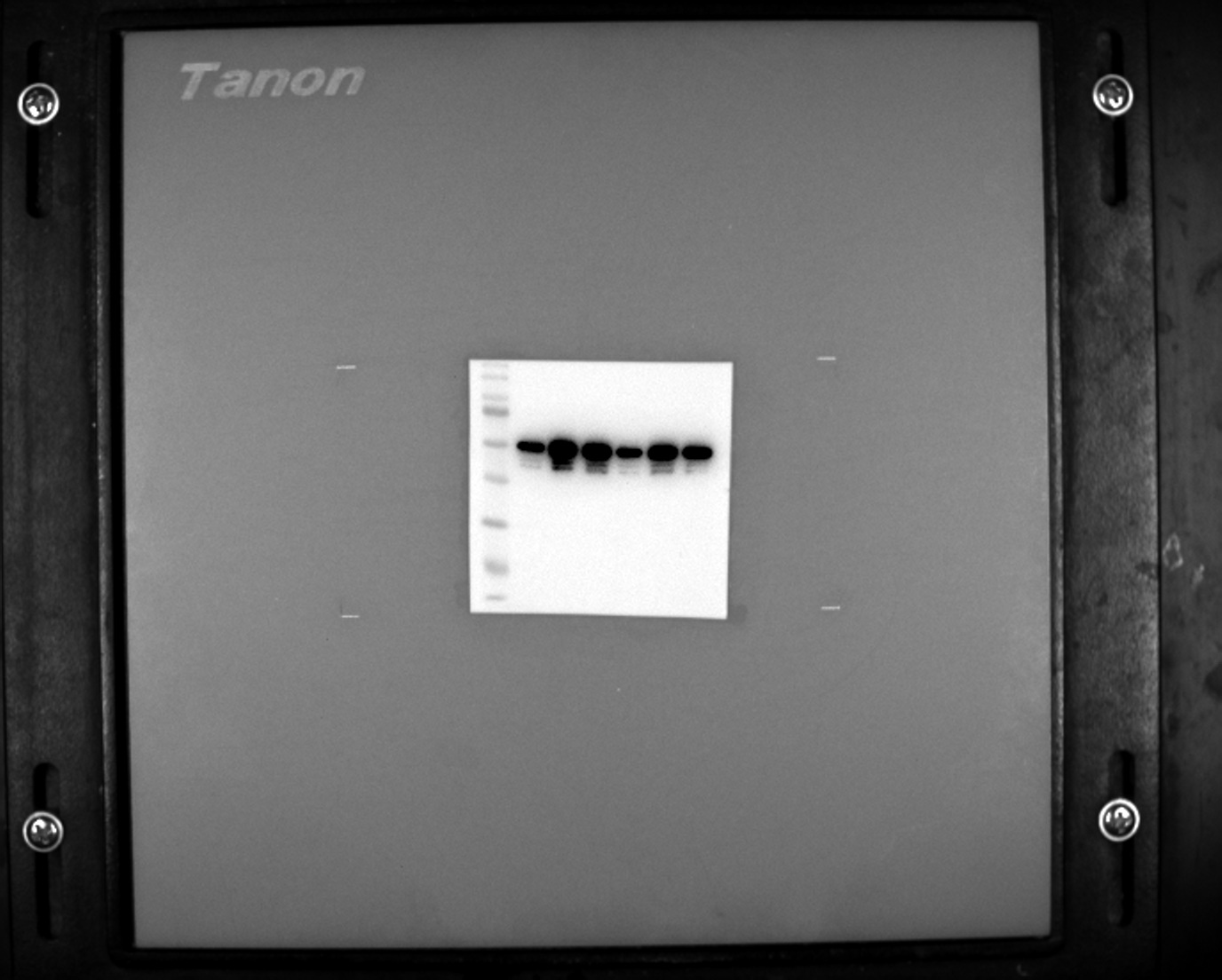

Supplement: Supplementary file 1 [file DataSheet1.zip › WB/p-AKT/3M.Tif]

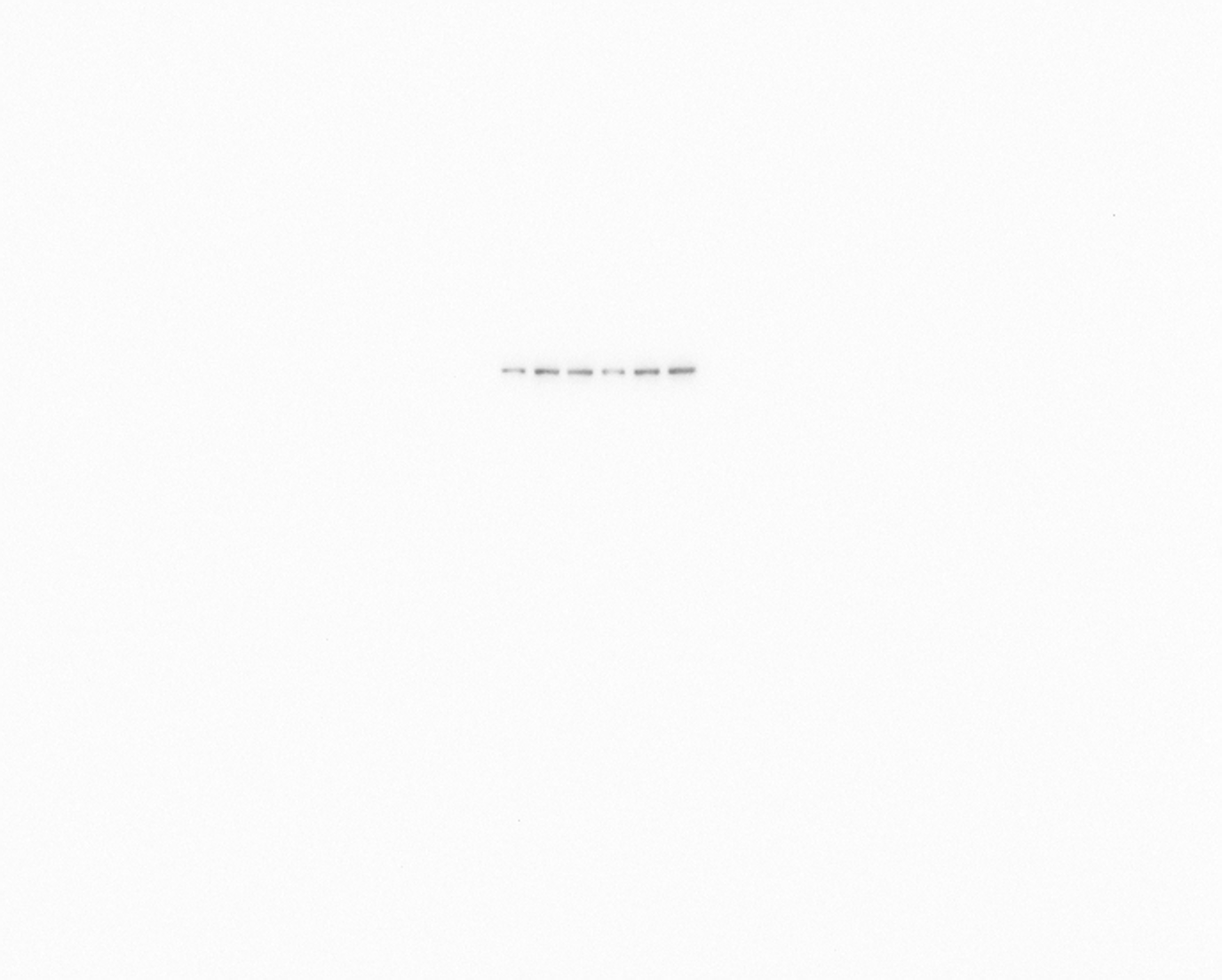

Supplement: Supplementary file 1 [file DataSheet1.zip › WB/p-mTOR/1.Tif]

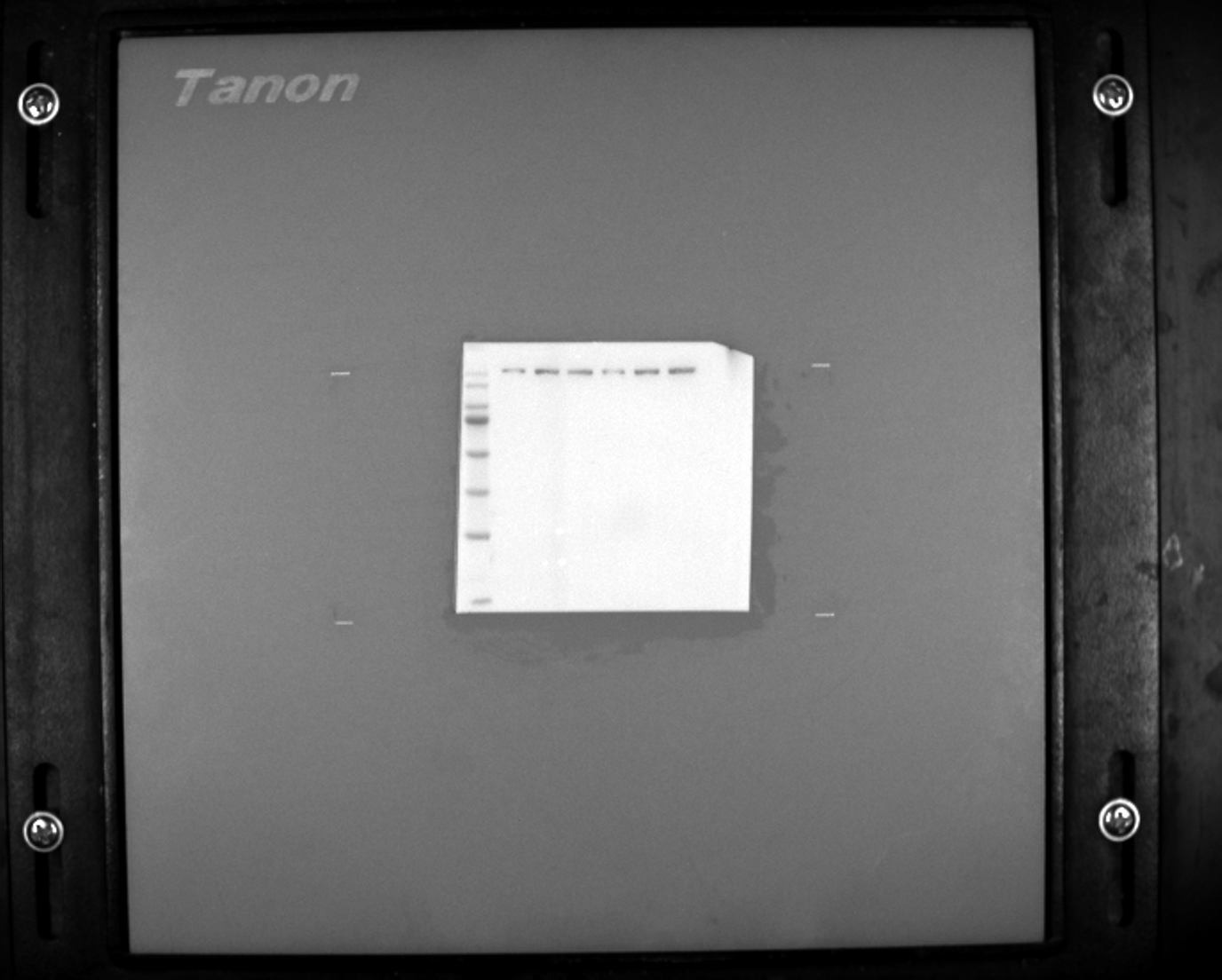

Supplement: Supplementary file 1 [file DataSheet1.zip › WB/p-mTOR/1M.Tif]

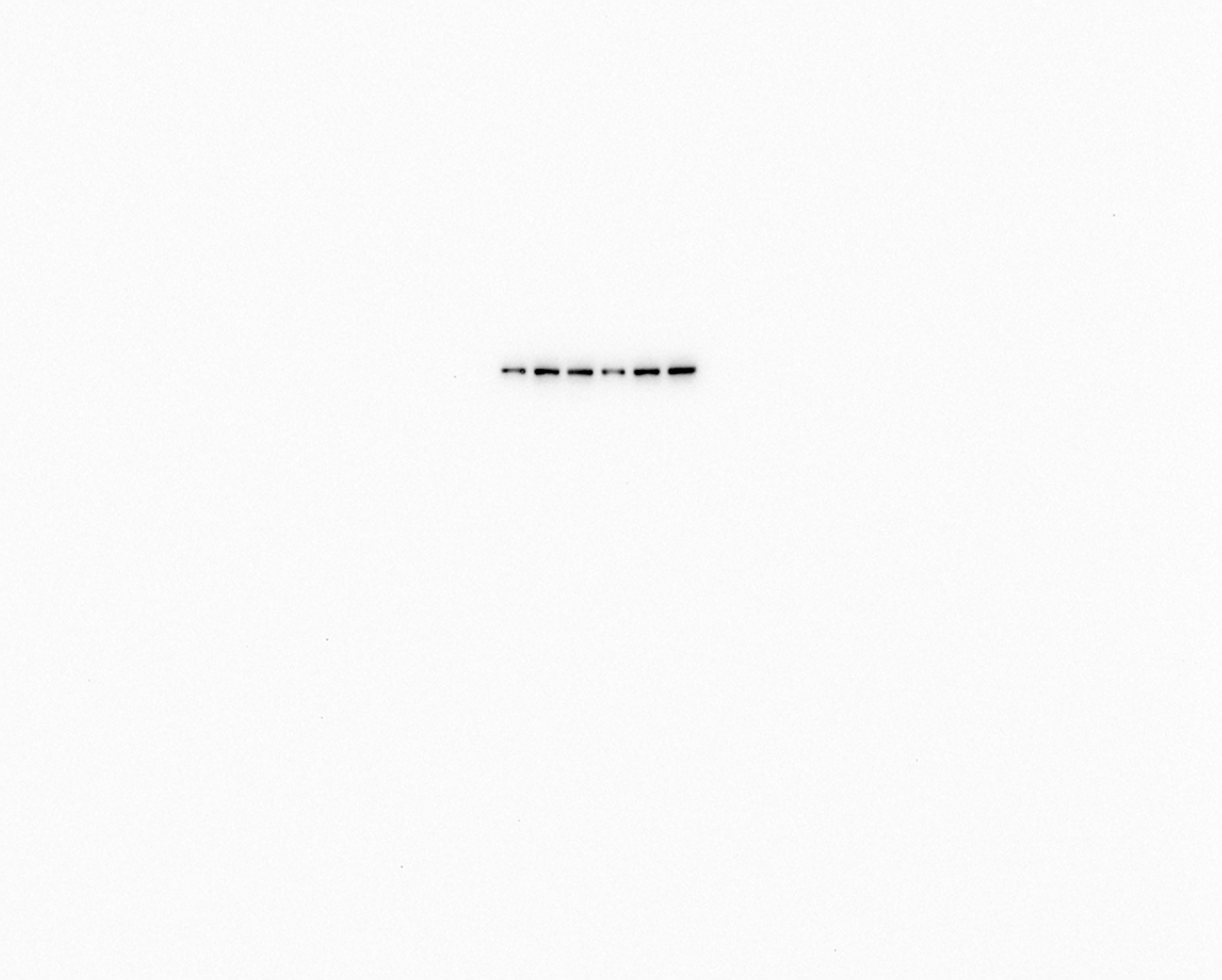

Supplement: Supplementary file 1 [file DataSheet1.zip › WB/p-mTOR/2.Tif]

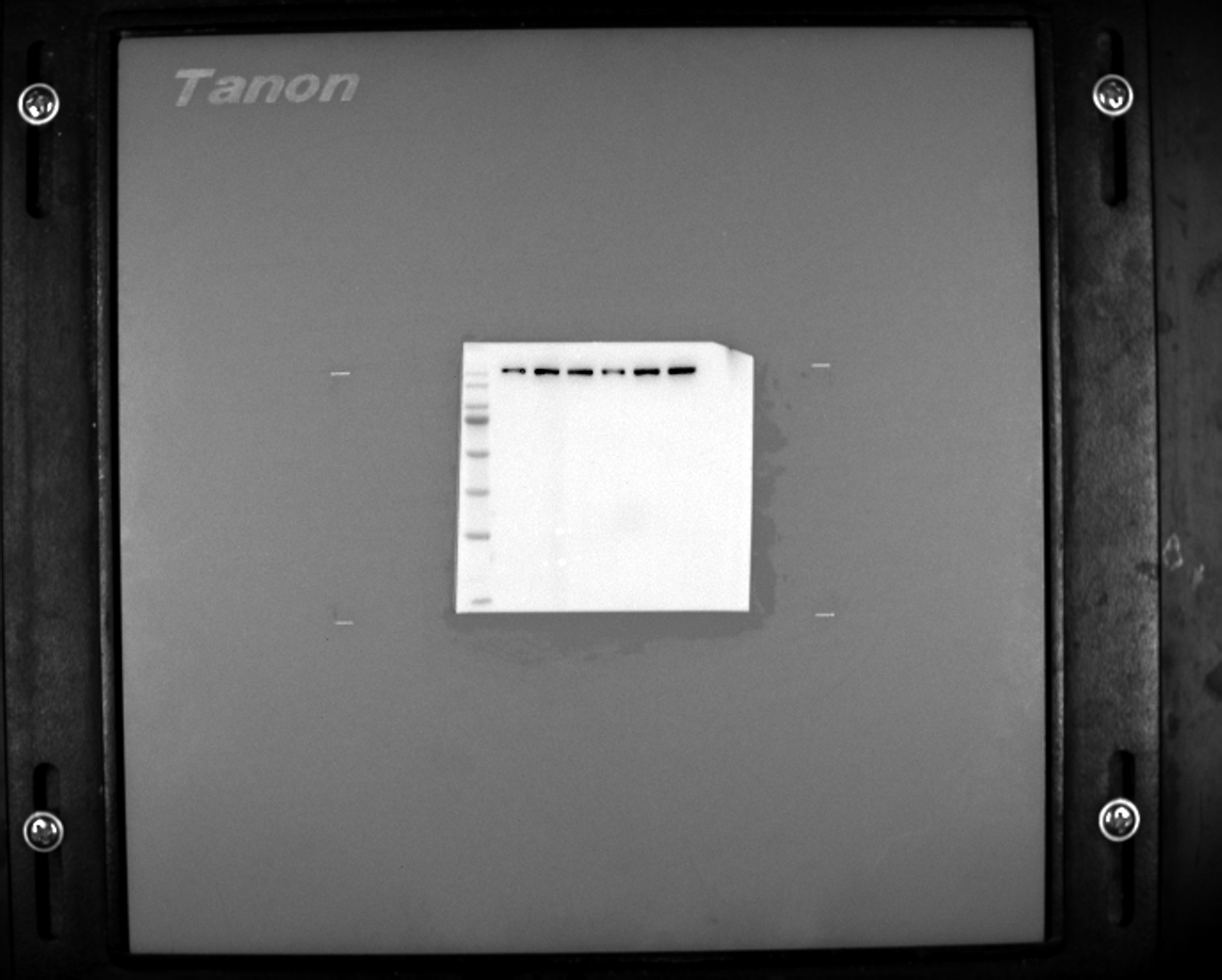

Supplement: Supplementary file 1 [file DataSheet1.zip › WB/p-mTOR/2M.Tif]

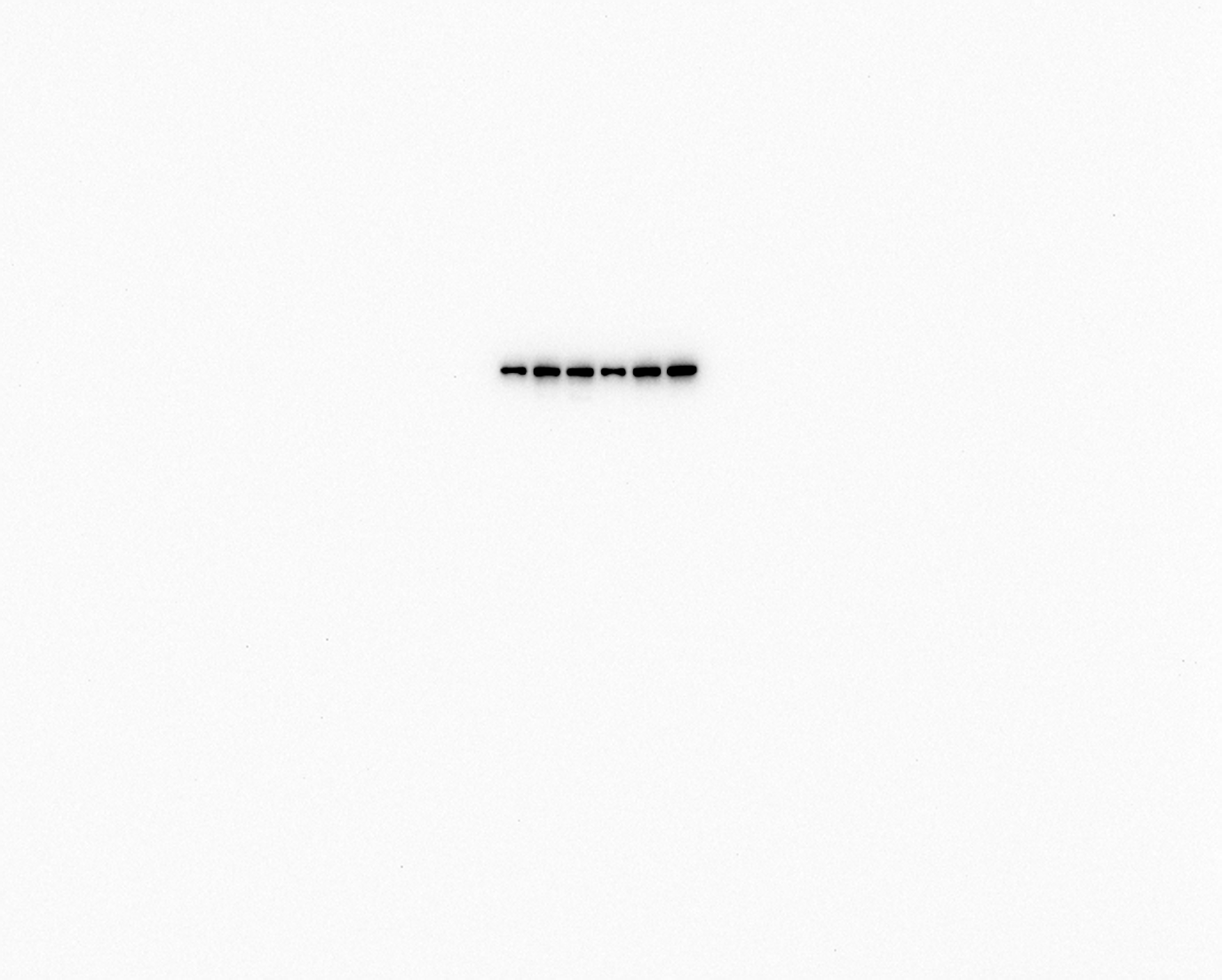

Supplement: Supplementary file 1 [file DataSheet1.zip › WB/p-mTOR/3.Tif]

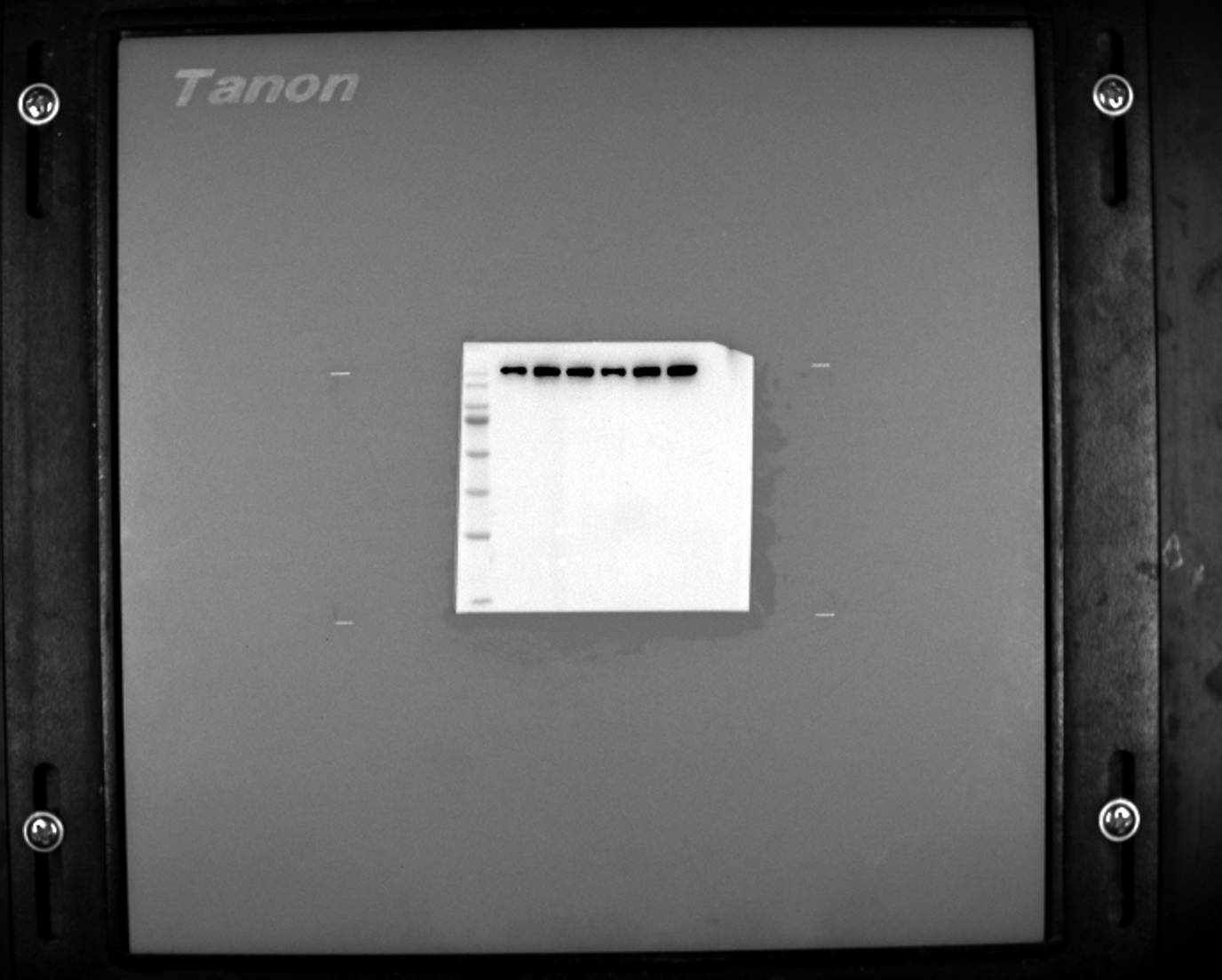

Supplement: Supplementary file 1 [file DataSheet1.zip › WB/p-mTOR/3M.Tif]

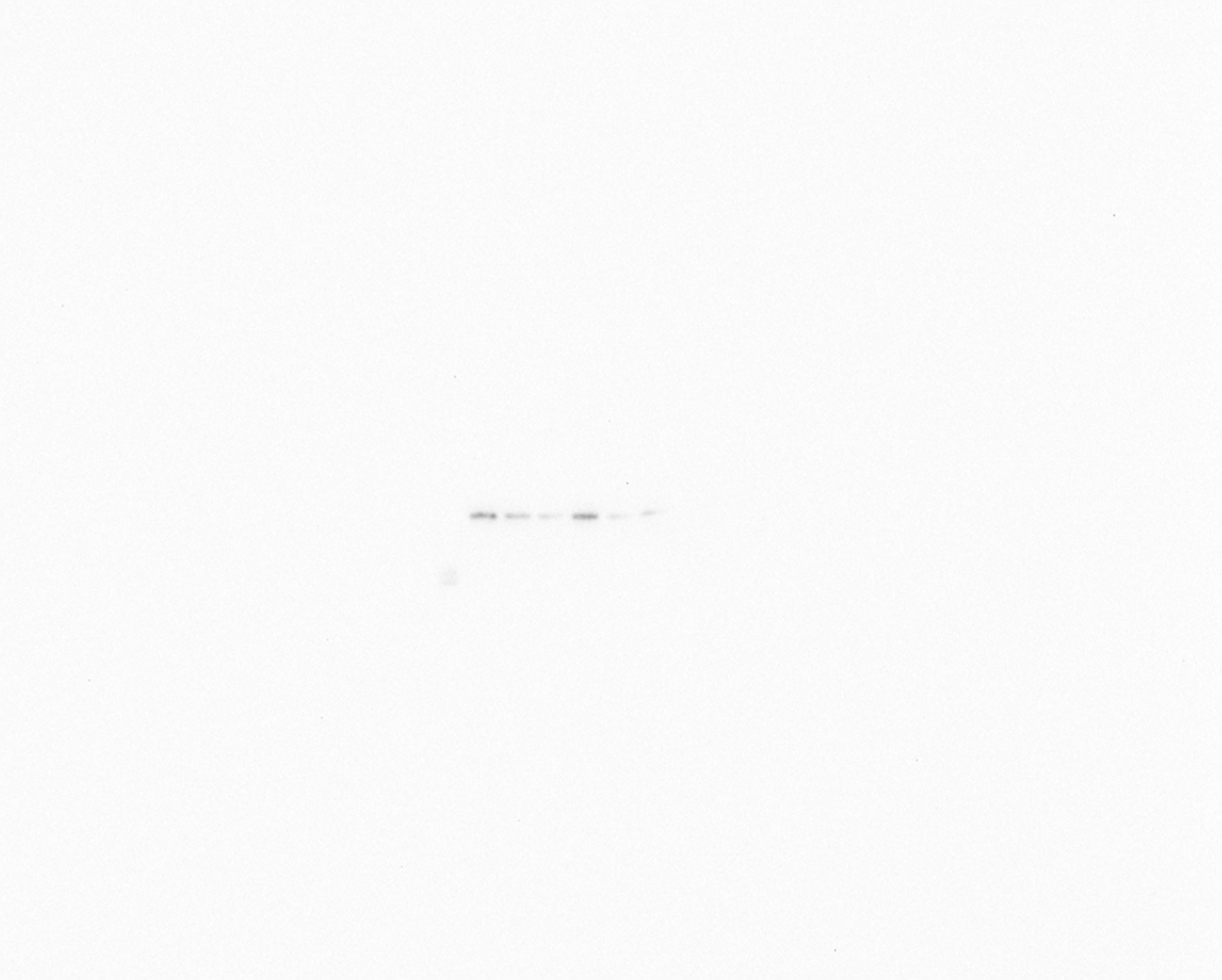

Supplement: Supplementary file 1 [file DataSheet1.zip › WB/siRNA/1.Tif]

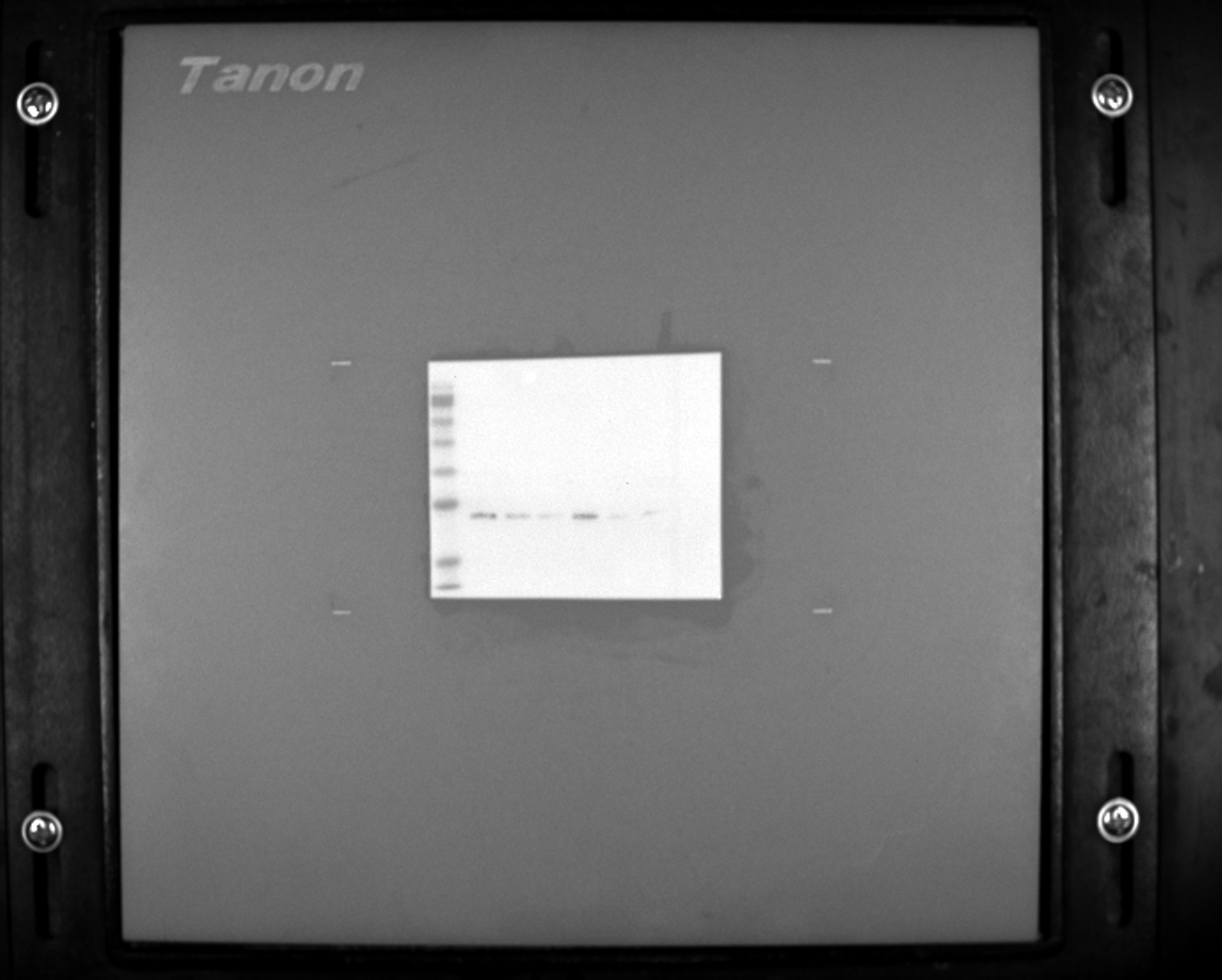

Supplement: Supplementary file 1 [file DataSheet1.zip › WB/siRNA/1M.Tif]

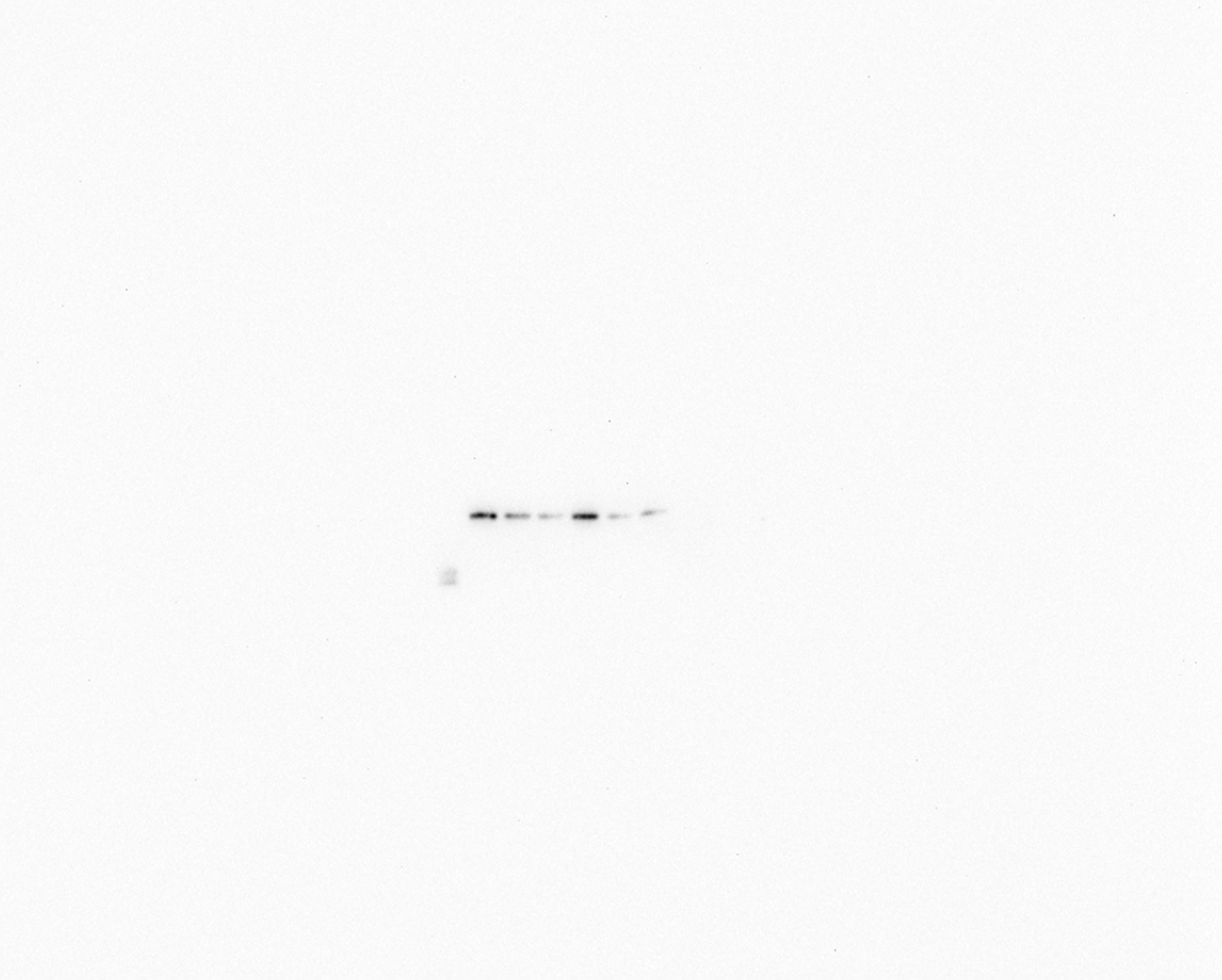

Supplement: Supplementary file 1 [file DataSheet1.zip › WB/siRNA/2.Tif]

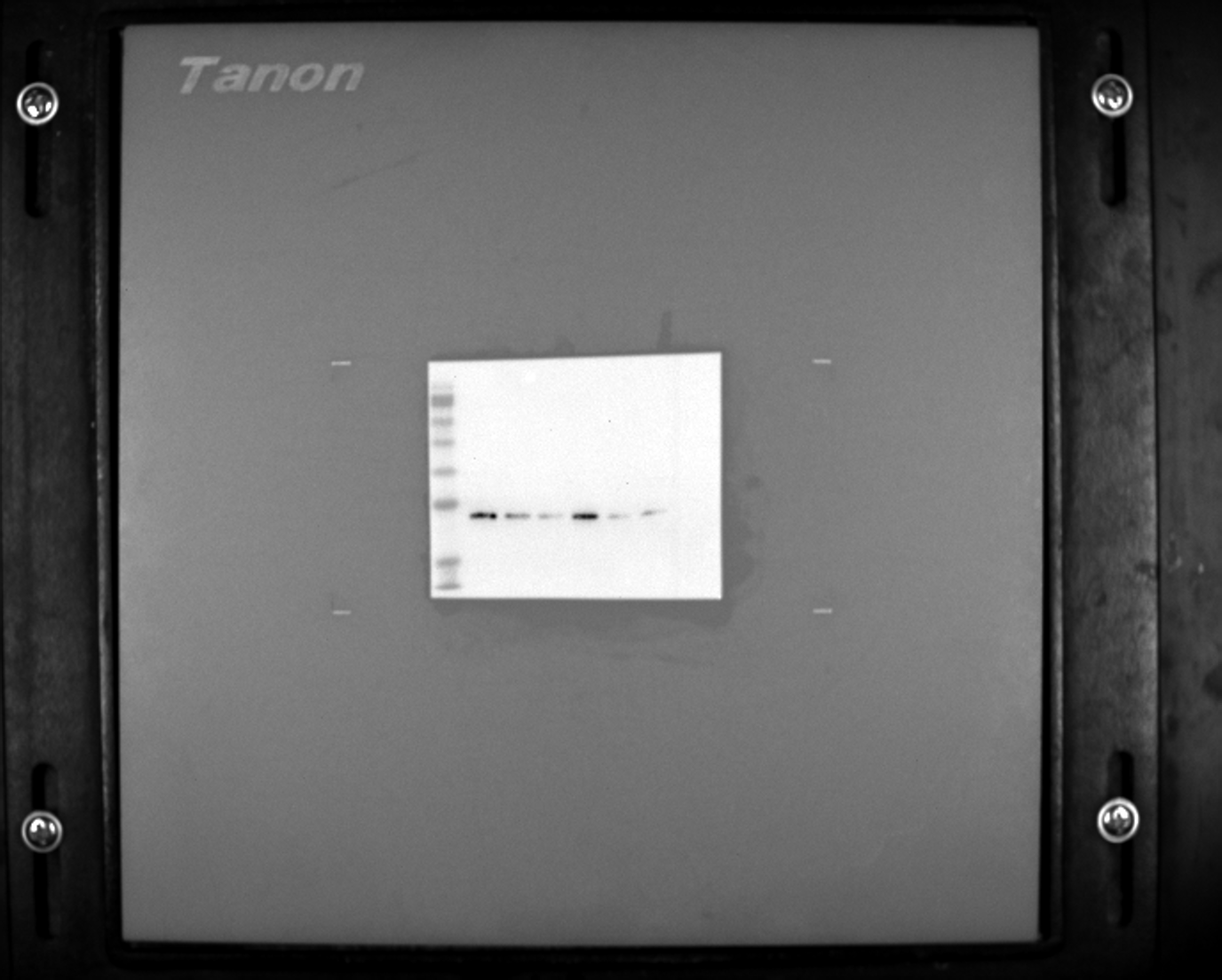

Supplement: Supplementary file 1 [file DataSheet1.zip › WB/siRNA/2M.Tif]

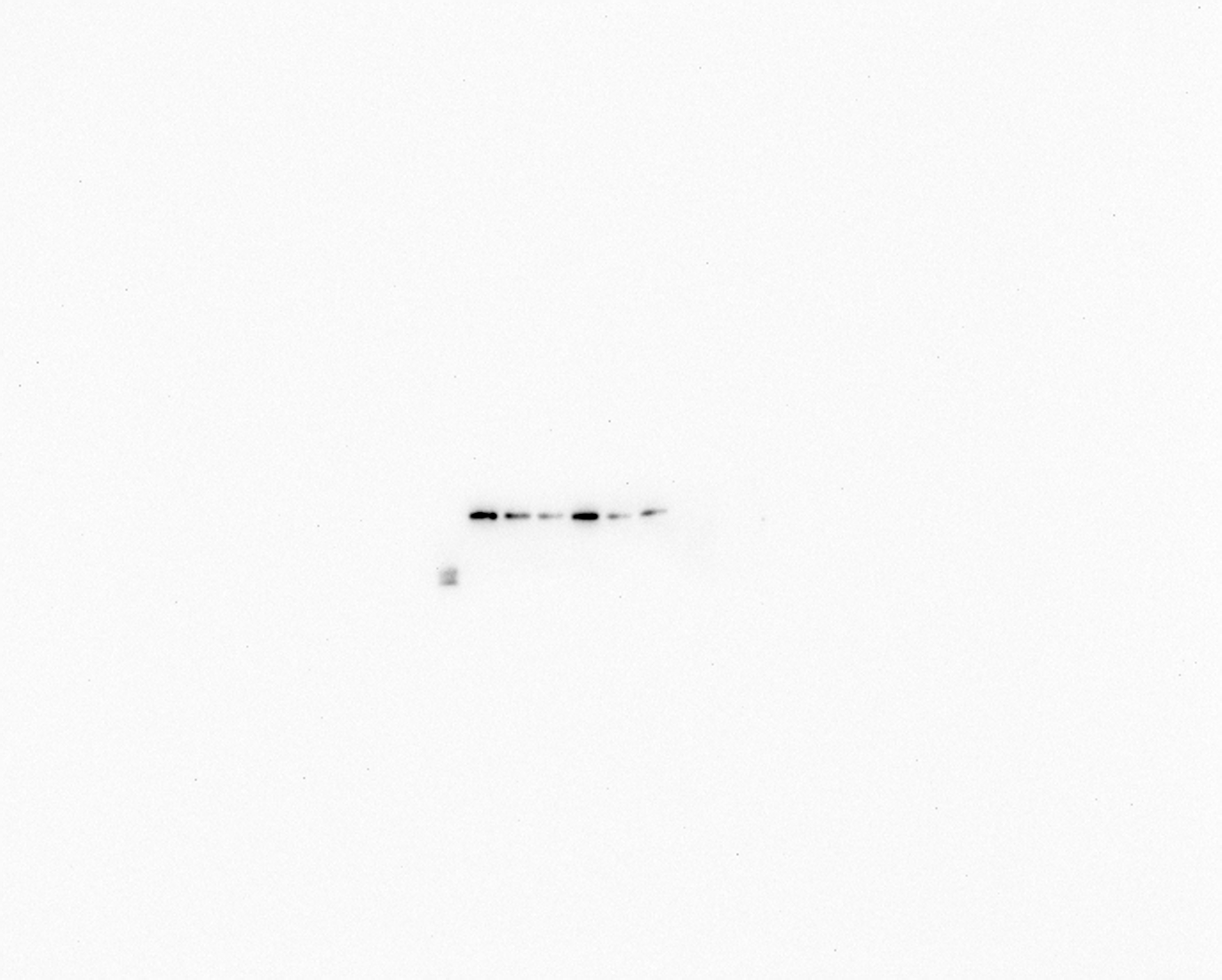

Supplement: Supplementary file 1 [file DataSheet1.zip › WB/siRNA/3.Tif]

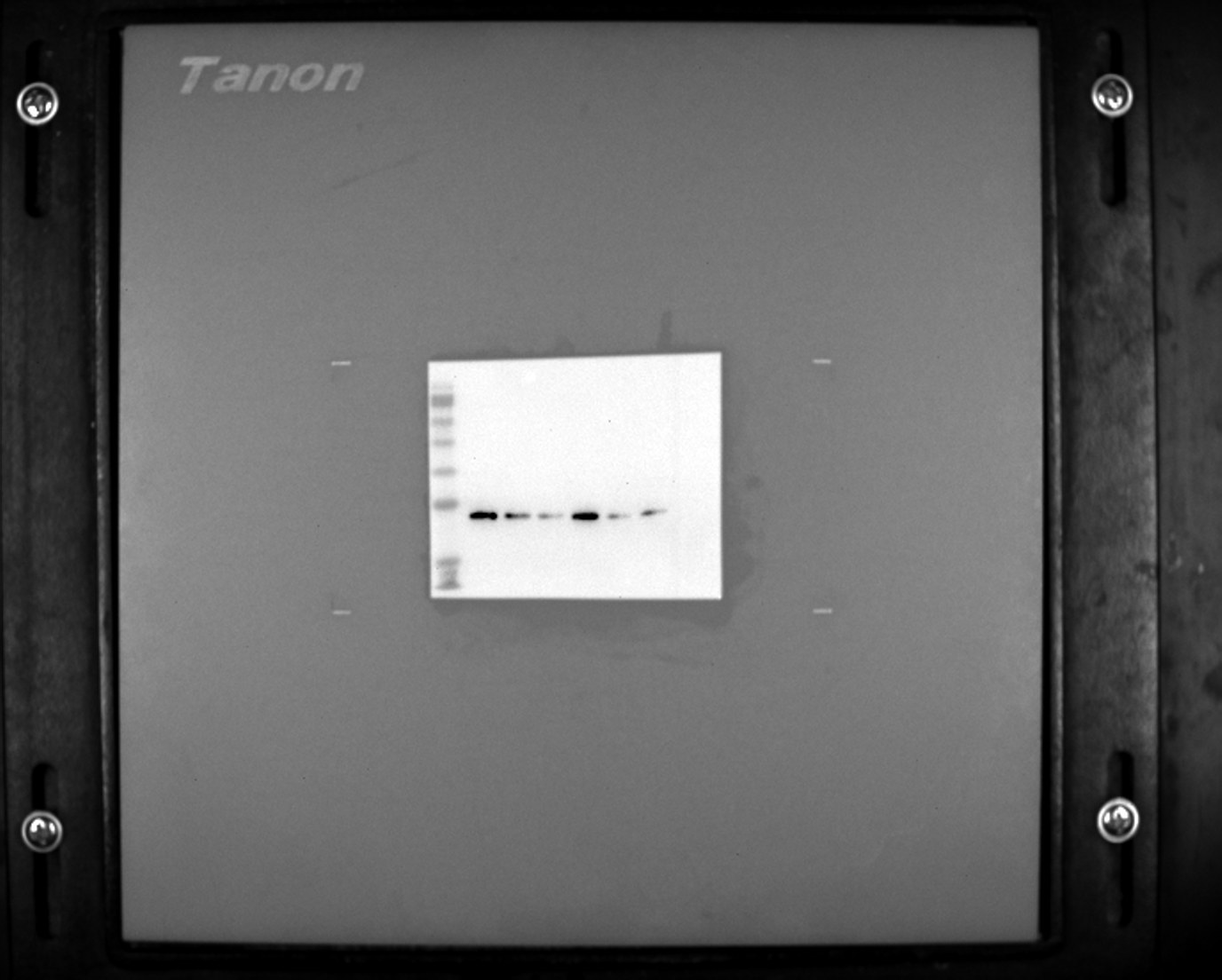

Supplement: Supplementary file 1 [file DataSheet1.zip › WB/siRNA/3M.Tif]

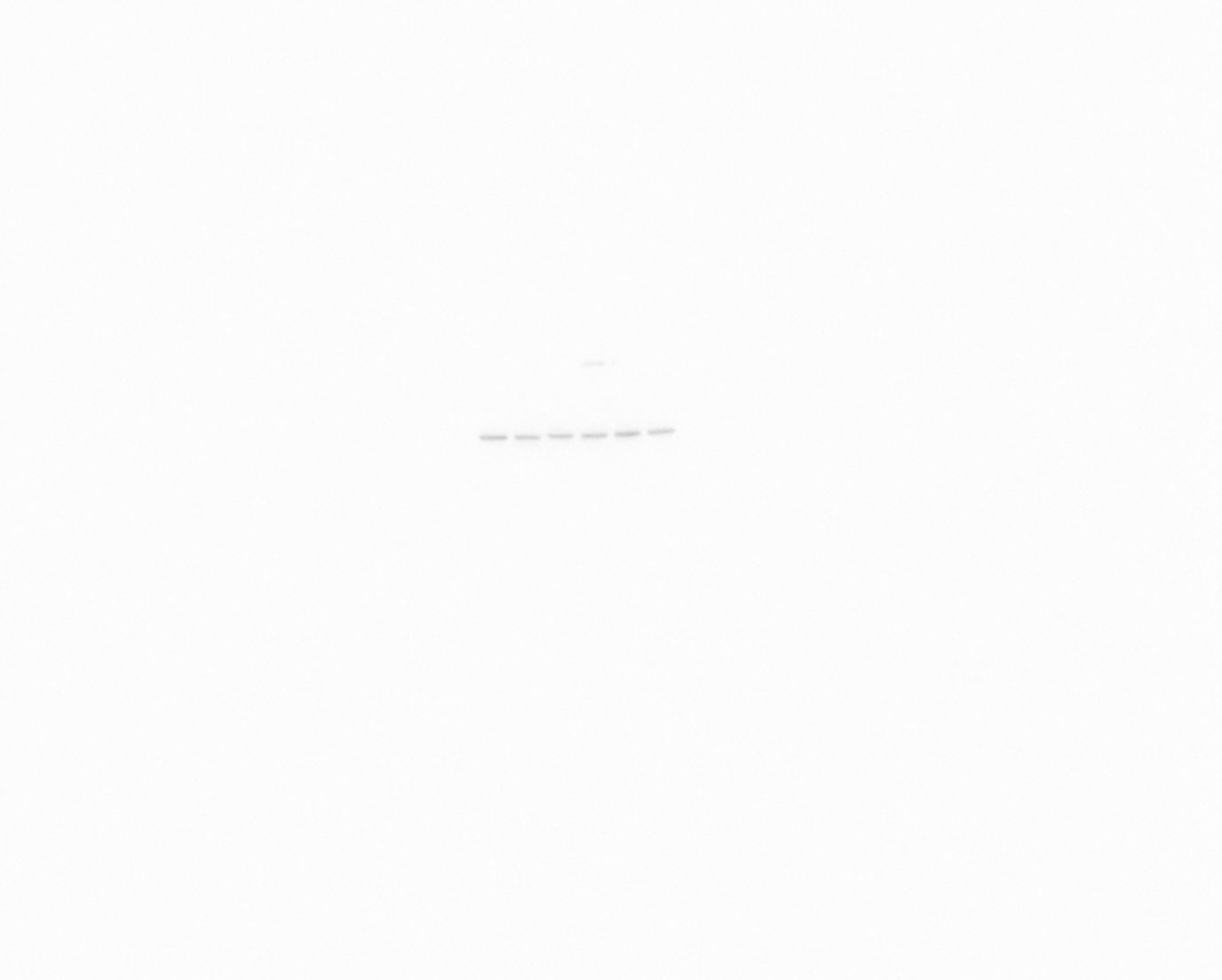

Supplement: Supplementary file 1 [file DataSheet1.zip › WB/siRNA/4.Tif]

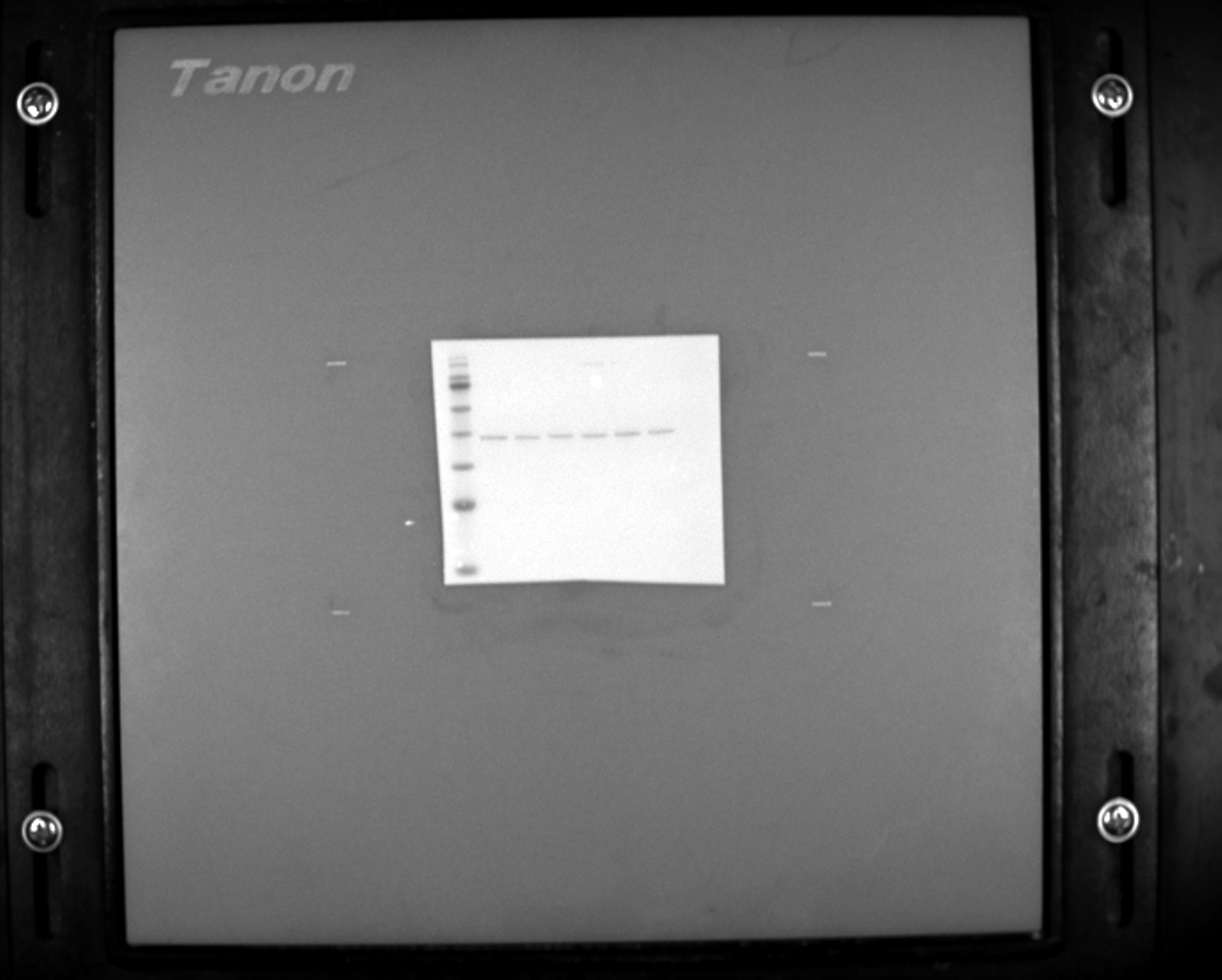

Supplement: Supplementary file 1 [file DataSheet1.zip › WB/siRNA/4M.Tif]

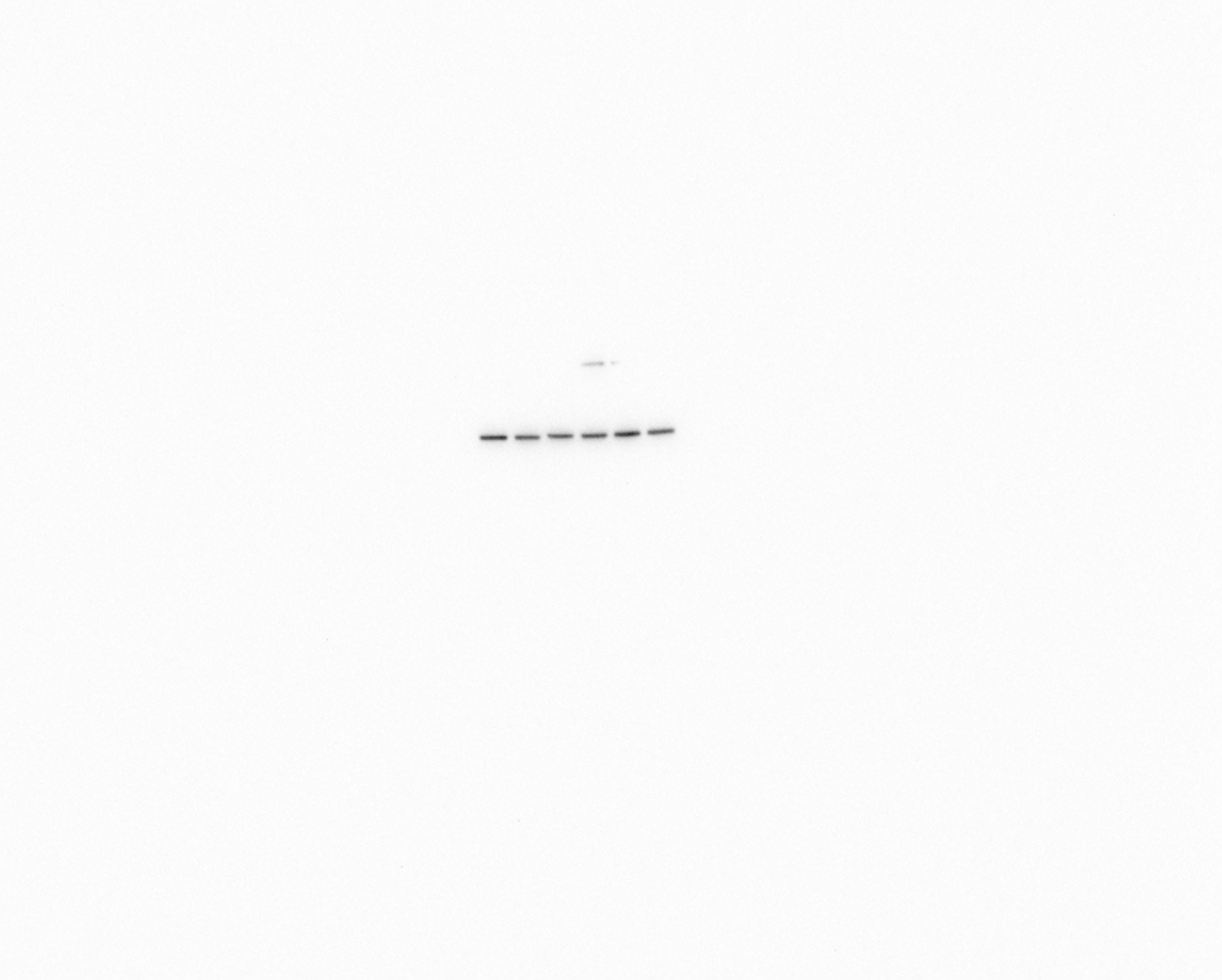

Supplement: Supplementary file 1 [file DataSheet1.zip › WB/siRNA/5.Tif]

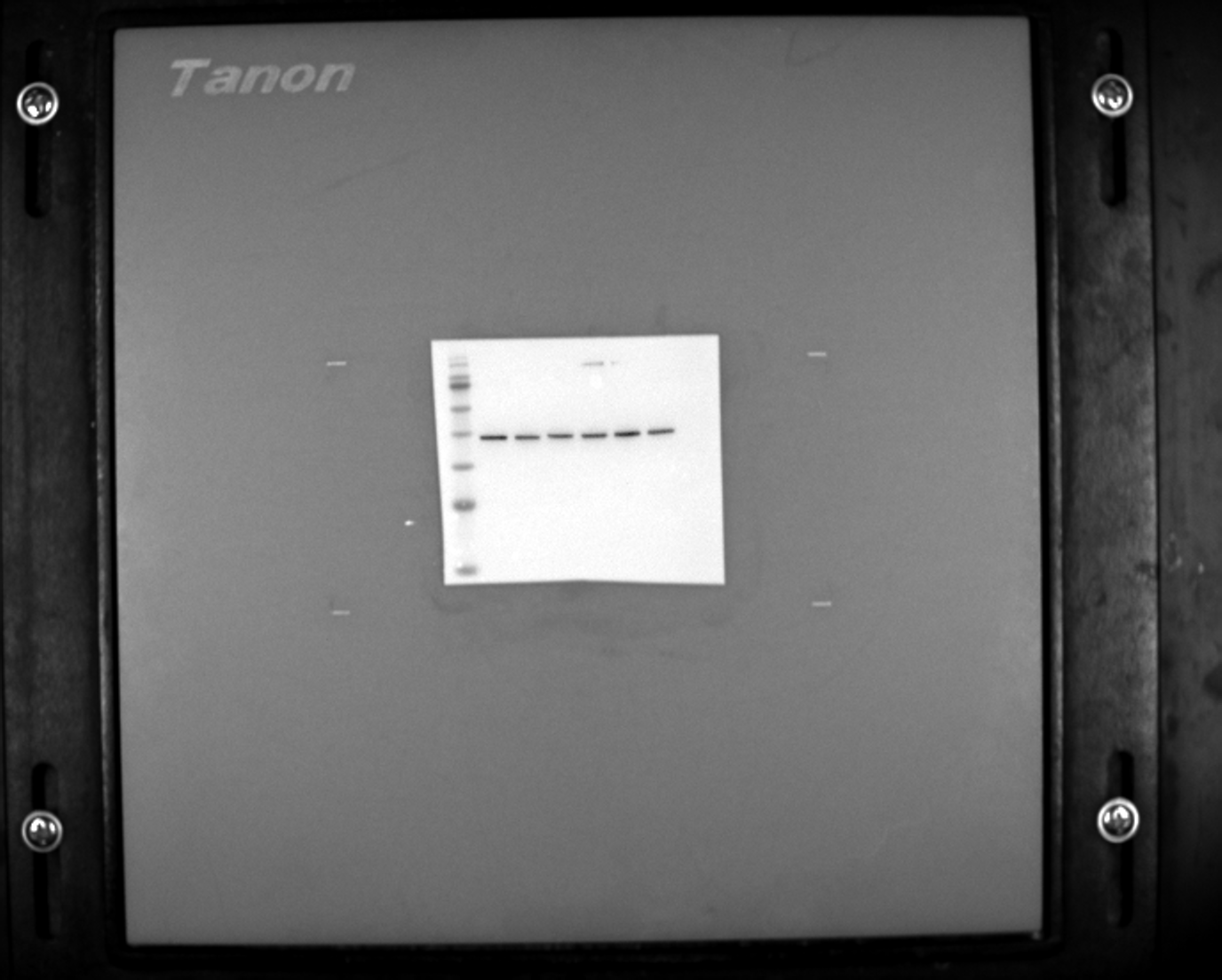

Supplement: Supplementary file 1 [file DataSheet1.zip › WB/siRNA/5M.Tif]

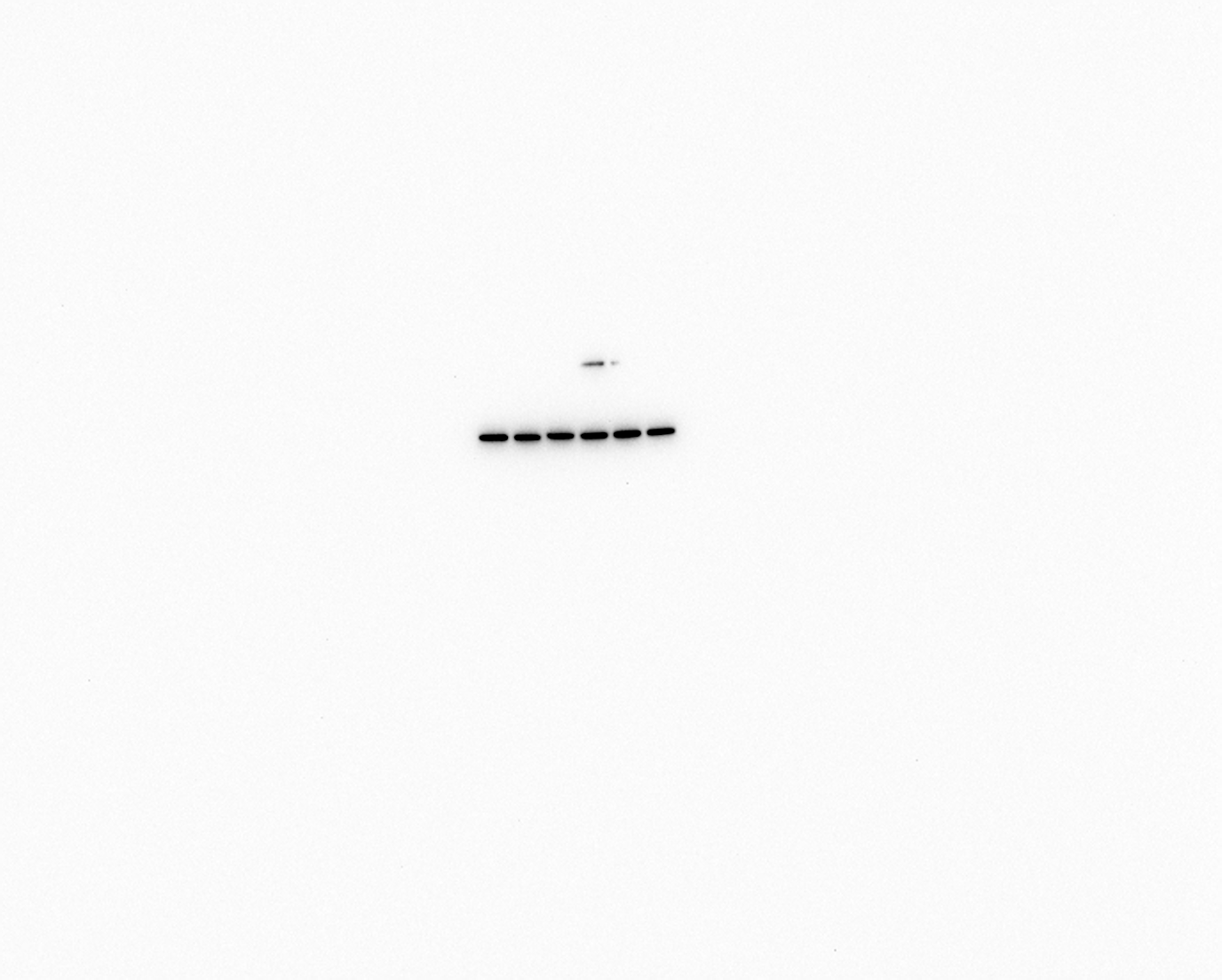

Supplement: Supplementary file 1 [file DataSheet1.zip › WB/siRNA/6.Tif]

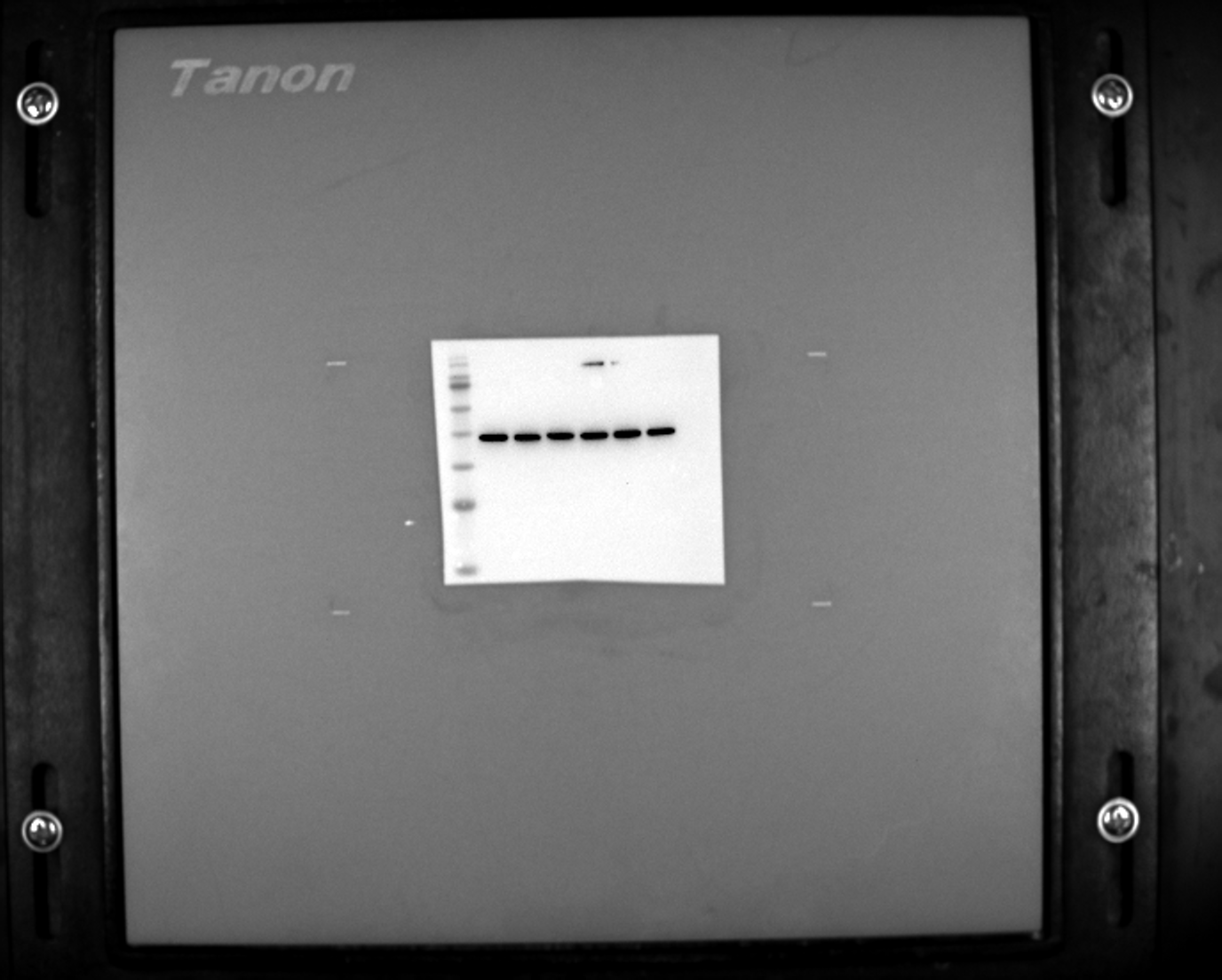

Supplement: Supplementary file 1 [file DataSheet1.zip › WB/siRNA/6M.Tif]
